# A multisystem, cardio-renal investigation of post-COVID-19 illness

**DOI:** 10.1038/s41591-022-01837-9

**Published:** 2022-05-23

**Authors:** Andrew J. Morrow, Robert Sykes, Alasdair McIntosh, Anna Kamdar, Catherine Bagot, Hannah K. Bayes, Kevin G. Blyth, Michael Briscoe, Heerajnarain Bulluck, David Carrick, Colin Church, David Corcoran, Iain Findlay, Vivienne B. Gibson, Lynsey Gillespie, Douglas Grieve, Pauline Hall Barrientos, Antonia Ho, Ninian N. Lang, Vera Lennie, David J. Lowe, Peter W. Macfarlane, Patrick B. Mark, Kaitlin J. Mayne, Alex McConnachie, Ross McGeoch, Christopher McGinley, Connor McKee, Sabrina Nordin, Alexander Payne, Alastair J. Rankin, Keith E. Robertson, Giles Roditi, Nicola Ryan, Naveed Sattar, Sarah Allwood-Spiers, David Stobo, Rhian M. Touyz, Gruschen Veldtman, Stuart Watkins, Sarah Weeden, Robin A. Weir, Paul Welsh, Ryan Wereski, CISCO-19 Consortium, Neil Basu, Ammani Brown, Elaine Butler, Stephen J. H. Dobbin, Andrew Dougherty, Laura Dymock, Kirsty Fallon, Lesley Gilmour, Tracey Hopkins, Jennifer S. Lees, Iain B McInnes, Evonne McLennan, Fiona Savage, Stefan Siebert, Nicola Tynan, Rosemary Woodward, Kenneth Mangion, Colin Berry

**Affiliations:** 1 British Heart Foundation Glasgow Cardiovascular Research Centre, University of Glasgow, Glasgow, UK; 2 Department of Cardiology, Queen Elizabeth University Hospital, Glasgow, UK; 3 Robertson Centre for Biostatistics, Institute of Health and Wellbeing, University of Glasgow, Glasgow, UK; 4 Department of Haemostasis and Thrombosis, Glasgow Royal Infirmary, NHS Greater Glasgow and Clyde Health Board, Glasgow, UK; 5 Department of Respiratory Medicine, Glasgow Royal Infirmary, NHS Greater Glasgow and Clyde Health Board, Glasgow, UK; 6 Department of Respiratory Medicine, Queen Elizabeth University Hospital, NHS Greater Glasgow and Clyde Health Board, Glasgow, UK; 7 Institute of Cancer Sciences, University of Glasgow, Glasgow, UK; 8 Leeds General Infirmary, St. James’s University Hospital, Leeds, UK; 9 Department of Cardiology, University Hospital Hairmyres, East Kilbride, UK; 10 West of Scotland Heart and Lung Centre, NHS Golden Jubilee, Clydebank, UK; 11 Department of Cardiology, Royal Alexandra Hospital, Paisley, UK; 12 Project Management Unit, Glasgow Clinical Research Facility, Greater Glasgow and Clyde Health Board, Glasgow, UK; 13 Department of Respiratory Medicine, Royal Alexandra Hospital, Glasgow, UK; 14 Department of Medical Physics, NHS Greater Glasgow and Clyde Health Board, Glasgow, UK; 15 MRC-University of Glasgow Centre for Virus Research, Institute of Infection and Immunity, University of Glasgow, Glasgow, UK; 16 Department of Cardiology, Aberdeen Royal Infirmary, Aberdeen, UK; 17 Department of Emergency Medicine, Queen Elizabeth University Hospital, NHS Greater Glasgow and Clyde Health Board, Glasgow, UK; 18 Electrocardiology Core Laboratory, Institute of Health and Wellbeing, University of Glasgow, Glasgow, UK; 19 Glasgow Renal and Transplant Unit, Queen Elizabeth University Hospital, NHS Greater Glasgow and Clyde Health Board, Glasgow, UK; 20 Department of Cardiology, University Hospital Crosshouse, Kilmarnock, UK; 21 Department of Radiology, NHS Greater Glasgow and Clyde Health Board, Glasgow, UK; 22 Scottish Adult Congenital Cardiac Services, NHS Golden Jubilee, Clydebank, UK; 23 British Heart Foundation Centre for Cardiovascular Science, University of Edinburgh, Edinburgh, UK

**Keywords:** Diagnostic markers, Thrombosis

## Abstract

The pathophysiology and trajectory of post-Coronavirus Disease 2019 (COVID-19) syndrome is uncertain. To clarify multisystem involvement, we undertook a prospective cohort study including patients who had been hospitalized with COVID-19 (ClinicalTrials.gov ID NCT04403607). Serial blood biomarkers, digital electrocardiography and patient-reported outcome measures were obtained in-hospital and at 28–60 days post-discharge when multisystem imaging using chest computed tomography with pulmonary and coronary angiography and cardio-renal magnetic resonance imaging was also obtained. Longer-term clinical outcomes were assessed using electronic health records. Compared to controls (*n* = 29), at 28–60 days post-discharge, people with COVID-19 (*n* = 159; mean age, 55 years; 43% female) had persisting evidence of cardio-renal involvement and hemostasis pathway activation. The adjudicated likelihood of myocarditis was ‘very likely’ in 21 (13%) patients, ‘probable’ in 65 (41%) patients, ‘unlikely’ in 56 (35%) patients and ‘not present’ in 17 (11%) patients. At 28–60 days post-discharge, COVID-19 was associated with worse health-related quality of life (EQ-5D-5L score 0.77 (0.23) versus 0.87 (0.20)), anxiety and depression (PHQ-4 total score 3.59 (3.71) versus 1.28 (2.67)) and aerobic exercise capacity reflected by predicted maximal oxygen utilization (20.0 (7.6) versus 29.5 (8.0) ml/kg/min) (all *P* < 0.01). During follow-up (mean, 450 days), 24 (15%) patients and two (7%) controls died or were rehospitalized, and 108 (68%) patients and seven (26%) controls received outpatient secondary care (*P* = 0.017). The illness trajectory of patients after hospitalization with COVID-19 includes persisting multisystem abnormalities and health impairments that could lead to substantial demand on healthcare services in the future.

## Abstract (web summary)

Deep clinical phenotyping at 28–60 days post-discharge of patients who had been hospitalized with COVID-19 and subsequent long-term follow-up with electronic health records reveal evidence of persistent cardio-renal involvement.

## Main

Self-reporting^1–4^ and population studies^5–8^ of post-COVID-19 illness trajectory have found that residual symptoms, such as fatigue, breathlessness and exercise intolerance, are common, potentially leading to increased demands on healthcare services. At the outset of the COVID-19 pandemic, clinical studies lacked a prospective evaluation of disease pathogenesis and/or health status and selectively recalled patients, introducing selection bias^3,7^. Few detailed prospective studies have been reported^9–13^, and multisystem imaging with clinical outcomes and contemporary controls are lacking. Pre-existing disease complicates attribution of causal inferences in COVID-19, and the pathophysiology and clinical significance of post-COVID-19 syndromes remain uncertain^10^.

The pathogenesis of multi-organ inflammation in COVID-19 may involve direct virus invasion through binding angiotensin-converting enzyme 2 (ACE2)^14,15^, cardio-renal inflammation^16^, endothelial dysfunction^16^, thrombotic microvascular angiopathy^17^, stress cardiomyopathy^16^ and drug toxicity^16^. These distinct mechanisms define subgroups with multi-organ involvement in COVID-19. Myocarditis may cause longer-term morbidity and mortality in these patients^18^. Previous studies using cardiovascular magnetic resonance imaging (MRI) in COVID-19 have reported imaging features of myocardial inflammation in 27–60%^19,20^ of patients. These studies were unrepresentative as they involved case selection based on troponin elevation and retrospective recall^19,20^. Lack of coronary artery imaging is also a limitation for attributing the etiology of myocardial injury, which becomes susceptible to ascertainment bias.

Based on the cardiovascular tropism of severe acute respiratory syndrome coronavirus 2 (SARS-CoV-2)^16^, we hypothesized, first, that the illness trajectory of post-COVID-19 syndromes involves hemostatic pathway activation and systemic inflammation during convalescence; second, that cardio-renal involvement associates with pre-existing cardiovascular disease; and third, that myocarditis after COVID-19 associates with persisting impairments in health status, including physical and psychological well-being and clinical outcomes. We investigated disease mechanisms using multisystem imaging, biomarkers and their changes over the short (<3 months) and medium (12–18 months) term. Health status and physical and psychological function were serially recorded using validated patient-reported outcome measures, and clinical outcomes and healthcare use were assessed using electronic health records.

## Results

**Fig. 1:**
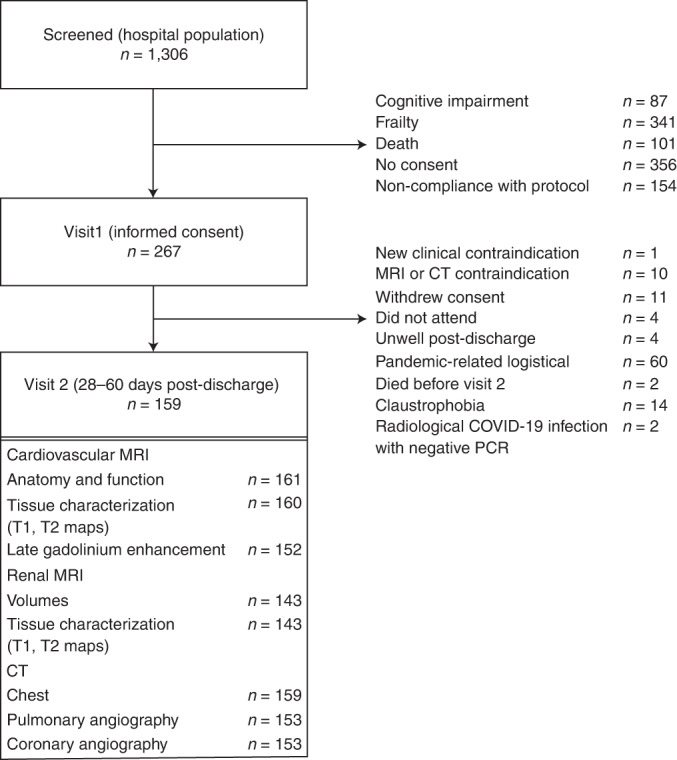
Flow diagram of the clinical study. The procedures involved screening hospitalized patients with COVID-19 defined by a PCR-positive result for SARS-CoV-2 in a nasopharyngeal swab and then obtaining written informed consent. The analysis population is defined by a PCR-positive result. Serial investigations were initiated in-hospital or early post-discharge (visit 1) and then repeated in association with multi-organ imaging at 28–60 days post-discharge (visit 2). Clinical follow-up continued for on average 450 days ± 88 s.d. (range, 290–627 days) post-discharge.

**Extended Data Fig. 1:**
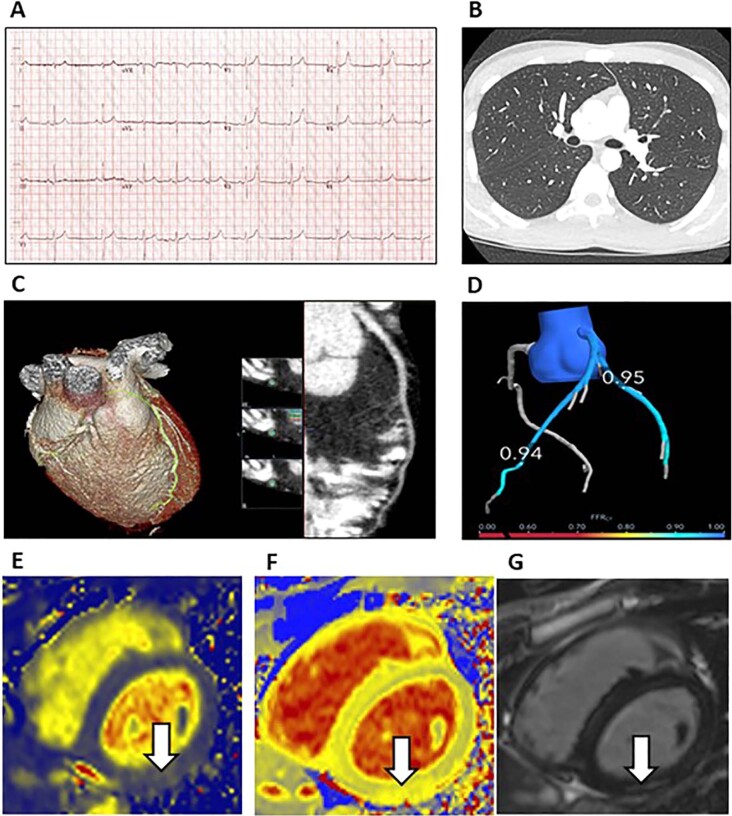
Myopericarditis associated with acute COVID-19 infection. A 19-year-old man with no past medical history presented with chest pain and dyspnoea. He had tested PCR-positive for SARS-CoV-2 in the community one week earlier. He experienced central burning chest pain which radiated to his jaw and left arm. The symptom lasted approximately 90 minutes. A 12-lead electrocardiogram revealed saddle-shaped ST-elevation in the precordial leads (**a**) and the peak concentration of high sensitivity troponin-I was 4,738 ng/L. No further episodes of chest pain occurred. A transthoracic echocardiogram revealed preserved biventricular function. Research-indicated chest computed tomography (CT) and pulmonary and coronary angiography (**b, c**) and cardio-renal magnetic resonance imaging (MRI) (**e, f, g**) were acquired in line with the protocol 28 days after discharge from hospital. There was no evidence of pulmonary embolism or COVID-19 pneumonitis (B). In the inferior wall (white arrow), localized, mid-wall elevations in myocardial native T2 (E, 47 ms) and T1 (F, 1270 ms) indicative of acute myocardial inflammation co-localized with sub-epicardial late gadolinium enhancement indicative of scar tissue (**g**). On coronary CT angiography, there was no angiographic evidence of atherosclerosis and the FFRct values derived in the left anterior descending (0.94) and left circumflex (0.95) coronary arteries were normal (FFRCT > 0.80) (**d**). The cardiac diagnosis adjudicated by the clinical event committee was myocarditis secondary to COVID-19.

**Extended Data Fig. 2:**
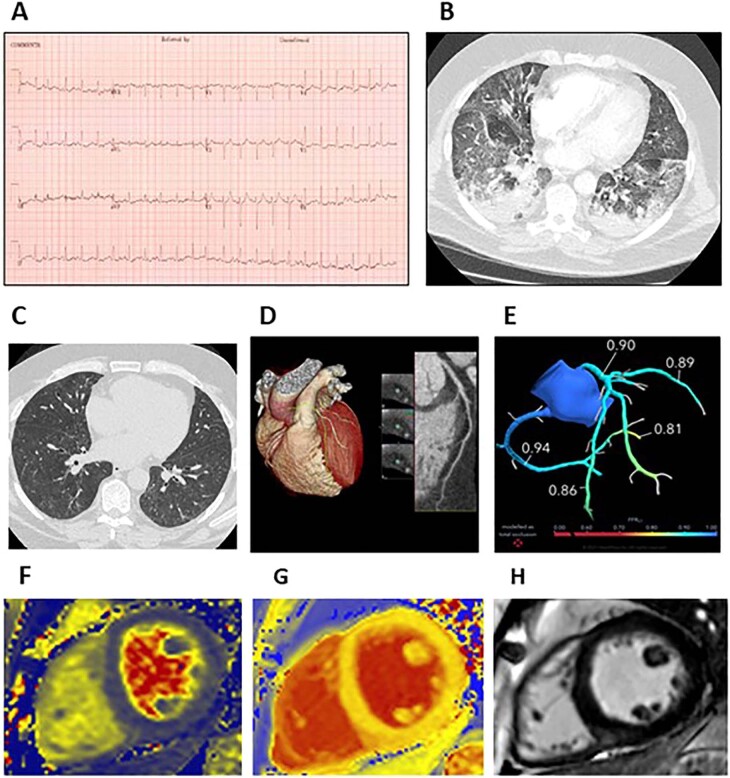
Myocardial injury in a patient treated in the Intensive Care Unit for COVID-19 pneumonitis and respiratory failure. A 58-year-old male healthcare worker was hospitalized with breathlessness, cough and pyrexia. There was no history of chest pain. Admission electrocardiogram (**a**) showed sinus tachycardia with premature atrial complexes and lateral ST-segment depression, and a peak troponin I concentration of 532 ng/L. The medical history included asthma and hypertension. A PCR test was positive for SARS-CoV-2. Due to respiratory distress and hypoxemia, the patient was intubated and admitted to the intensive care (ICU). A computed tomography (CT) pulmonary angiogram (8) revealed COVID pneumonitis and pulmonary thromboembolism was excluded. The ICU admission lasted one-month and the patient was discharged after a period of 52 days in hospital. The research CT scan revealed resolution of changes in the lung parenchyma (**c**). Coronary CT angiography revealed atherosclerosis in the left coronary artery (**d**) and FFRCT (**e**) excluded obstructive coronary artery disease. Protocol-directed cardio-renal magnetic resonance imaging (MRI) did not reveal features of myocardial inflammation. Specifically, the myocardial T2 (F, 41 ms) and T1 (G, 1218 ms) relaxation times were normal and there was no intra-myocardial late gadolinium enhancement other than at the right ventricular insertion point, which can be a normal finding (H). The adjudicated cardiac diagnosis was acute myocardial injury secondary to hypoxemia, not myocarditis.

**Extended Data Fig. 3:**
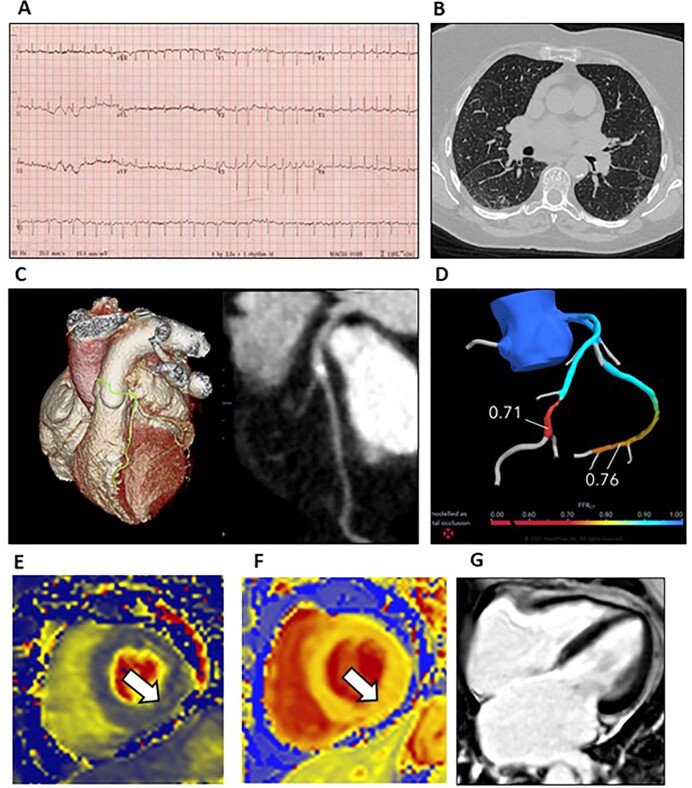
COVID-19 infection associated with type 2 myocardial infarction and atrial fibrillation. A 68-year-old woman presented with breathlessness, a five-day febrile illness and falls due to weakness but without loss of consciousness. The admission electrocardiogram (**a**) revealed atrial fibrillation of presumed recent onset and a rapid ventricular rate and there were clinical signs of heart failure. Following admission, a swab PCR test for SARS-CoV-2 infection was positive. Protocol-directed coronary computed tomograph (CT) angiography revealed a dominant left coronary artery. The Agatston calcium score was 156 (815 percentile for age, gender, ethnicity) and there was atherosclerosis in the left coronary artery (**c**). The FFRCT ratios in the mid-left anterior descending (FFRCT = 0.71) and distal circumflex (FFRCT = 0.76) coronary arteries were reduced (abnormal < 0.80) (**d**). Parametric mapping revealed increases in myocardial T2 (47 ms) and T1 (1269 ms) relaxation times consistent with myocardial inflammation (**e, f**). Late gadolinium contrast-enhanced imaging was normal. There was bi-atrial enlargement and the left and right ventricular ejection fractions were preserved (**g**). The adjudicated diagnosis was acute myocardial injury and type 2 myocardial infarction in association with pre-existing coronary artery disease and acute COVID-19.

**Extended Data Fig. 4:**
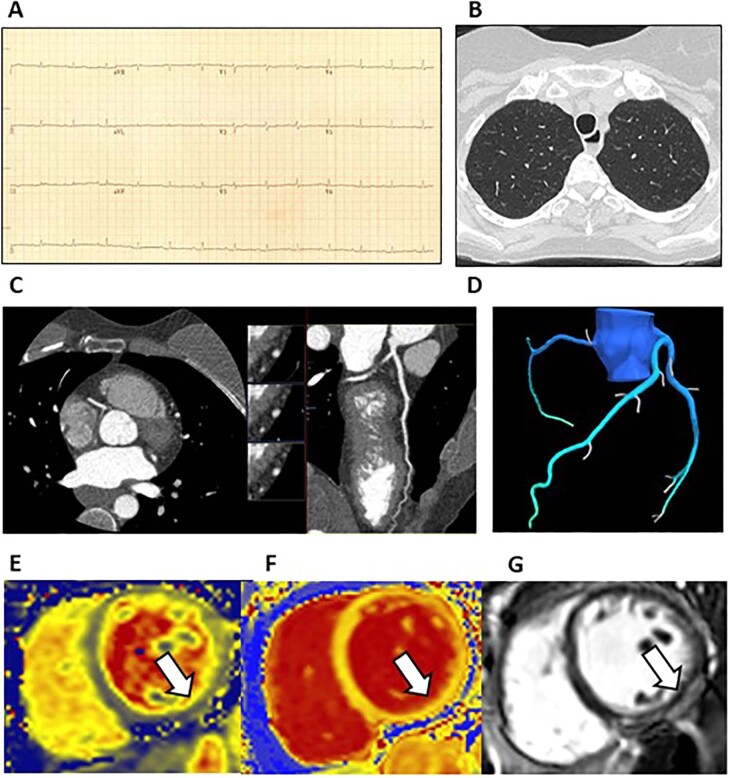
Myocarditis associated with acute COVID-19 infection. A 51-year-old woman with no relevant past medical history presented with chest pain and dyspnoea. She had tested PCR-positive for SARS-CoV-2 in the community 8 days previously. She experienced breathlessness, anosmia, fever, and central chest pain which radiated to her jaw. A 12-lead electrocardiogram revealed T wave flattening laterally (**a**) and the peak concentration of high sensitivity troponin-1 was 56 ng/L. No further episodes of chest pain occurred. Research-indicated computed tomography (CT) (**b, c**) and cardio-renal magnetic resonance imaging (MRI) (**e, f, g**) were acquired in line with the protocol 27 days after discharge from hospital. There was no evidence of pulmonary embolism or COVID-19 pneumonitis (B). On coronary CT angiography, there was no angiographic evidence of atherosclerosis and the FFRct values were normal (**d**). In the inferior wall of the left ventricle (white arrow), localized, mid-wall elevations in myocardial native T2 (E, 54 ms) and T1 (F, 1313 ms) relaxation times, indicative of acute myocardial inflammation, co-localized with sub epicardial myocardial late gadolinium enhancement (**g**). These imaging features are diagnostic of myocarditis. The cardiac diagnosis adjudicated by the clinical event committee was myocarditis secondary to COVID-19.

**Extended Data Fig. 5:**
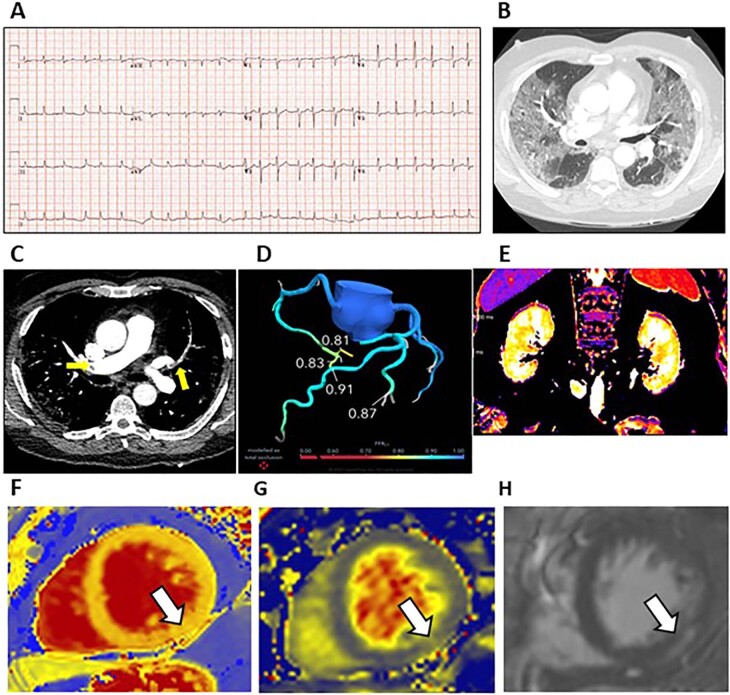
Persisting cardio-renal abnormalities due to COVID-19 infection. A 66-year-old man with history of hypertension was admitted with increasing breathlessness and transient syncope. There was no history of chest pain. The PCR test confirmed SARS-CoV-2 infection and the D-Dimer concentration was 35,656 ng/L. The admission electrocardiogram revealed atrial fibrillation with a rapid ventricular rate requiring electrocardioversion due to haemodynamic compromise (**a**). The computed tomography (CT) pulmonary angiogram revealed COVID-19 pneumonitis (**b**) and bilateral pulmonary arterial thrombus involving both central and peripheral branches (**c**). Treatment dose low molecular weight heparin was initially prescribed followed by directoral anticoagulation. Despite additional treatment with tocilizumab and dexamethasone for covid pneumonitis, intubation and mechanical ventilation were necessary during a 4-day admission to the intensive care unit (ICU). The patient recovered, supported by treatment with inhaled oxygen and a reducing dose of dexamethasone. At visit 2, one month later, the research coronary CT angiogram and FFRct excluded obstructive coronary artery disease (**d**). Protocoldirected MRI revealed tissue inflammation in the kidney (increased renal T1 (**e**, cortex 1666ms, medulla 2060 ms), and myocardium (native T1 (**f**, 1303 ms), and native T2 (**g**, 49 ms) associated with late gadolinium enhancement localized to the inferior left ventricular wall with a sub-epicardial distribution (**h**) in keeping with myocardial inflammation. The CT chest scan revealed substantially improvement in the lung parenchyma, and the previously identified pulmonary emboli were no longer evident.

**Extended Data Fig. 6:**
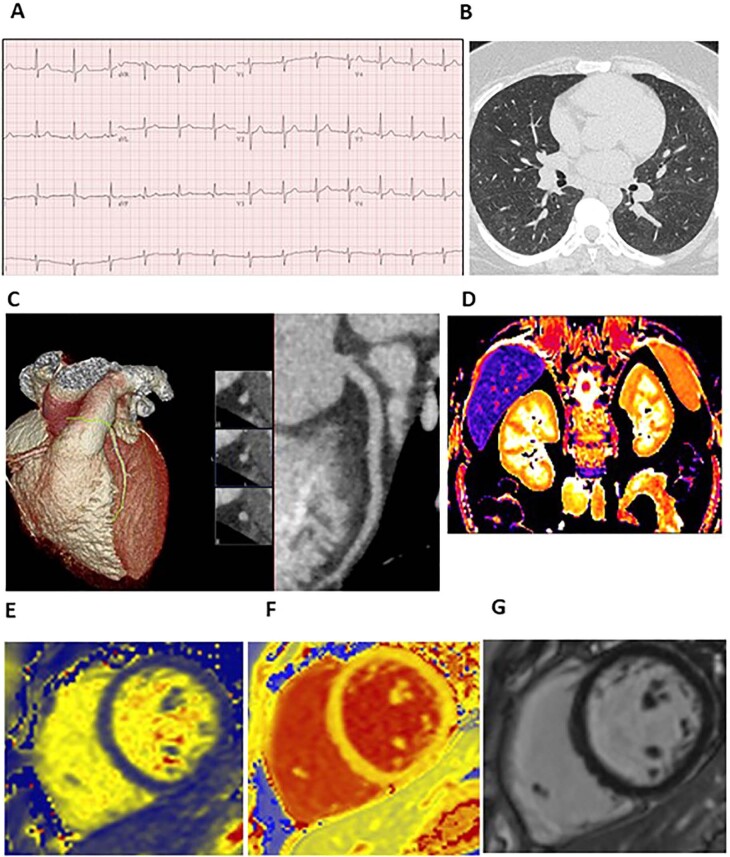
COVID-19 without cardio-renal involvement: no abnormalities identified. A 25-year-old woman presented with breathlessness, lightheadedness and anosmia 9 days after a nasopharyngeal swab tested PCR-positive for SARS-COV-2 infection. The admission electrocardiogram (**a**) revealed normal sinus rhythm. She was treated with dexamethasone. Research-indicated chest computed tomography (CT) (**b**, **c**) and cardio-renal magnetic resonance imaging (MRI) (**d, e, f, g**) were acquired in line with the protocol 23 days after discharge from hospital. High resolution lung CT revealed faint peribronchovascular ground glass opacification in keeping with recovering pneumonitis. The coronary CT angiogram was normal (C). Renal MRI imaging revealed normal T1 values (D: cortex 1481 ms, medulla 1922ms). There was no evidence of raised T2 (E: 38 ms) or T1 (1218 ms) values on parametric mapping, and no myocardial late gadolinium enhancement. The cardiac diagnosis adjudicated by the dinical event committee was no evidence of myocardial injury.

In total, 1,306 patients were screened between 22 May 2020 and 16 March 2021, and 267 patients provided written informed consent. The flow diagram is shown in Fig. 1, and example clinical cases are provided in Extended Data Figs. 1–6.

In total, 159 patients were evaluated at 28–60 days after the last episode of hospital care. Their average age was 55 years, 139 (87%) were White, 14 (9%) were Asian, four (3%) were Arab, two (1%) were Black, 69 (43%) were female, 74 (46%) had a history of cardiovascular disease or treatment, 61 (40%) were in the highest quintile of social deprivation and 36 (23%) were healthcare workers (Table 1 and Supplementary Tables 1 and 2). Clinical disease severity scores are described in Table 1. Twenty-two (15%) patients had normal chest radiology during the index hospitalization. Two (1.2%) patients had received a single dose of SARS-CoV-2 vaccine before hospitalization (Supplementary Table 3). Regarding COVID-19 therapy, 109 (69%) received oxygen, 89 (56%) received steroids, 42 (26%) received antiviral drug therapy, 31 (20%) received non-invasive respiratory support and 14 (9%) received invasive ventilation.

**Table 1:** Clinical characteristics of the study population by likelihood of adjudicated myocarditis post-COVID-19

	COVID-19	Controls		Myocarditis				
			*P* value	Not present	Unlikely	Probable	Very likely	*P* value^a^
	*n* = 159	*n* = 29		*n* = 17 (11%)	*n* = 56 (35%)	*n* = 65 (41%)	*n* = 21 (13%)	
Demographic								
Age ± s.d., years	54.5 ± 11.9	57.3 ± 9.6	0.373	56.9 ± 11.4	55.1 ± 13.3	53.1 ± 11.4	54.9 ± 10.1	0.525
Male sex, *n* (%)	90 (56.6)	18 (62.1)	0.685	13 (76.5)	35 (62.5)	33 (50.8)	9 (42.9)	0.115
Female sex, *n* (%)	69 (43.4)	11 (37.9)		4 (23.5)	21 (37.5)	32 (49.2)	12 (57.1)	
**Most deprived SIMD quintile (Q1),** ***n*** **(%)**	61 (40.4)	5 (17.9)	**0.032**	4 (25.0)	20 (37.0)	25 (41.0)	12 (60.0)	0.178
Healthcare worker, *n* (%)	36 (22.6)	5 (17.9)	0.804	1 (5.9)	10 (17.9)	18 (27.7)	7 (33.3)	0.121
Ethnicity, *n* (%)								
White	139 (87.4%)	26 (89.7%)	0.694	16 (94.1%)	51 (91.1%)	54 (83.1%)	18 (85.7%)	0.848
Asian	14 (8.8%)	3 (10.3%)		1 (5.9%)	3 (5.4%)	8 (12.3%)	2 (9.5%)	
Other	6 (3.8%)	0 (0.0%)		0 (0.0%)	2 (3.6%)	3 (4.6%)	1 (4.8%)	
Presenting characteristics, mean (s.d.)								
Body mass index, kg m^−^^2^	30.5 (7.1)	30.7 (5.0)	0.554	30.9 (5.6)	29.6 (5.8)	31.1 (8.7)	30.6 (6.4)	0.829
**Heart rate, bpm**	95 (19)	69 (15)	**<0.001**	98 (19)	94 (20)	95 (16)	94 (25)	0.586
**Systolic blood pressure, mmHg**	129 (20)	142 (19)	**0.003**	122 (24)	135 (18)	126 (20)	124 (17)	0.139
Diastolic blood pressure, mmHg	77 (13)	82 (16)	0.058	74 (13)	79 (12)	77 (13)	74 (12)	0.458
**Peripheral oxygen saturation, %**	93 (7)	97 (2)	**<0.001**	91 (10)	94 (5)	94 (6)	94 (9)	0.758
**Respiratory rate, minutes**	24 (12)	14 (4)	**<0.001**	23 (5)	23 (11)	25 (16)	21 (6)	0.312
WHO clinical severity score, *n* (%)								
**No evidence of infection**	0 (0.0)	29 (100.0)	**<0.001**	0 (0.0)	0 (0.0)	0 (0.0)	0 (0.0)	0.101
**Hospitalized, no oxygen therapy**	50 (31.4)	0 (0.0)		3 (17.6)	17 (30.4)	24 (36.9)	6 (28.6)	
**Oxygen therapy by mask or nasal prongs**	74 (46.5)	0 (0.0)		8 (47.1)	30 (53.6)	28 (43.1)	8 (38.1)	
**Non-invasive ventilation**	20 (12.6)	0 (0.0)		4 (23.5)	7 (12.5)	8 (12.3)	1 (4.8)	
**Mechanical ventilation**	5 (3.1)	0 (0.0)		0 (0.0)	0 (0.0)	3 (4.6)	2 (9.5)	
**Ventilation with organ support**	10 (6.3)	0 (0.0)		2 (11.8)	2 (3.6)	2 (3.1)	4 (19.0)	
COVID-19 diagnosis, *n* (%)								
**PCR test**	159 (100)	0 (0.0)	**<0.001**	17 (100.0)	56 (100.0)	65 (100)	21 (100)	1.000
Nosocomial	7 (4.4)	0 (0.0)	0.598	0 (0.0)	4 (7.1)	3 (4.6)	0 (0.0)	0.666
Antibody test^δ^	0 (0.0)	29 (100)	**<0.001**					
Radiology, chest radiograph or CT scan, *n* (%)								
**Typical features of COVID-19**	109 (74.7)	–		12 (75.0)	40 (78.4)	41 (68.3)	16 (84.2)	**0.024**
**Atypical features of COVID-19**	11 (7.5)	–		2 (12.5)	3 (5.9)	4 (6.7)	2 (10.5)	
**Unlikely**	4 (2.7)	–		2 (12.5)	0 (0.0)	1 (1.7)	1 (5.3)	
**Normal**	22 (15.1)	–		0 (0.0)	8 (15.7)	14 (23.3)	0 (0.0)	
Acute COVID-19 therapy, *n* (%)								
Oxygen	109 (68.6)	–		14 (82.4)	39 (69.6)	41 (63.1)	15 (71.4)	0.509
Steroid	89 (56.0)	–		12 (70.6)	31 (55.4)	36 (55.4)	10 (47.6)	0.557
Antiviral	42 (26.4)	–		9 (52.9)	15 (26.8)	14 (21.5)	4 (19.0)	0.075
Non-invasive respiratory support	31 (19.5)	–		5 (29)	9 (16.1)	11 (16.9)	6 (28.6)	0.386
**Intensive care**	24 (15.1)	–		5 (29.4)	5 (8.9)	8 (12.3)	6 (28.6)	**0.048**
**Invasive ventilation**	14 (8.8)	–		2 (11.8)	1 (1.8)	5 (7.7)	6 (28.6)	**0.004**
Intravenous inotrope	7 (4.4)	–		1 (5.9)	2 (3.6)	1 (1.5)	3 (14.3%)	0.092
Cardiovascular history, *n* (%)								
Cardiovascular disease and/or treatment	74 (46.5)	14 (48.3)	1.000	8 (47.1)	29 (51.8)	26 (40.0)	11 (52.4)	0.560
Risk scores, mean (s.d.)								
**ISARIC4C in-hospital mortality risk, %**	12.1 (10.6)	6.9 (8.4)	**0.003**	14.0 (10.7)	13.2 (11.4)	10.4 (9.4)	12.8 (11.7)	0.426
Q-Risk 3, 10-year cardiovascular risk, %	13.5 (11.1)	13.1 (10.0)	0.984	12.5 (7.9)	15.5 (12.8)	12.0 (9.7)	14.3 (13.1)	0.724
Charlson Comorbidity Index	1.9 (1.8)	1.5 (1.2)	0.412	1.7 (1.9)	2.1 (2.0)	1.9 (1.8)	1.6 (1.2)	0.793
Laboratory results, index admission								
Initial hemoglobin, mean (s.d.), g/L	141 (16)	143 (12)	0.655	142 (15)	140 (17)	140 (15)	143 (16)	0.624
**Initial platelet count, mean (s.d.), ×10**^**9**^**/L**	237 (94)	259 (58)	**0.006**	264 (137)	217 (75)	244 (93)	248 (95)	0.344
**Initial lymphocyte count, mean (s.d.), ×10**^**9**^**/L**	1.5 (4.7)	1.9 (0.6)	**<0.001**	1.0 (0.5)	1.1 (0.5)	2.1 (7.3)	1.4 (0.6)	0.215
**Peak D-dimer, mean (s.d.), ng/ml**	1,740 (5,439)	311 (303)	**0.026**	2,022 (4,159)	916 (2,132)	1,754 (6,648)	3,127 (7,431)	0.881
Minimum eGFR, ml/min/1.73 m^2^	82 (27)	78 (29)	0.799	80 (27)	85 (23)	84 (26)	69 (37)	0.454
**AKI,** ***n*** **(%)**	20 (14)	0 (0.0)	1.000	3 (19)	2 (4)	9(16)	6 (33)	**0.008**
Peak high-sensitivity troponin I, median (IQR), ng/L	4.0 (3.0, 13.0)	4.0 (4.0, 4.0)	0.358	6.0 (4.0, 10.0)	4.0 (3.0, 10.0)	4.0 (3.0, 9.0)	30.0 (3.5, 83.8)	0.158
**Peak ferritin, mean (s.d.), mg/L**	360 (182, 864)	118 (69, 166)	**<0.001**	454 (184, 835)	359 (212, 1,082)	332 (159, 692)	562 (198, 1,860)	0.441
**Peak CRP, median (IQR), mg/L**	104 (37, 181)	2 (1, 5)	**<0.001**	130 (77, 180)	107 (45, 164)	91 (35, 181)	121 (17, 350)	0.584
**HbA1c, mean mmol/mol Hb, %**	48 (18)	44 (22)	**0.020**	57 (32)	50 (18)	45 (14)	45 (19)	0.100
**Initial albumin, mean, g/L**	34 (5)	40 (5)	**<0.001**	32 (5)	35 (4)	34 (6)	35 (5)	0.273
Timelines								
Hospitalized, *n* (%)	143 (90)	3 (10)	**<0.001**	16 (94)	53 (95)	54 (83)	20 (95)	0.162
Duration of admission, median (IQR), days	5 (3, 11)	–	–	5 (4, 12)	5 (2, 10)	6 (3, 10)	4 (2, 29)	0.822
Symptom onset to primary outcome, median (IQR), days	65 (20)	–	–	66 (13)	62 (15)	65 (18)	73 (38)	0.850

An expanded version is provided in the Supplementary Table 1.

Ethnicity: Indian (0), Pakistani (0), Bangladeshi (0), Other Asian *n* = 14 (8.8%), Black Caribbean (0), Black African *n* = 2 (1.2%), Chinese *n* = 1 (0.6%), Other *n* = 1 (0.9%), White, *n* = 139 (87.4%). Missing data in the COVID-19 group COVID-19: D-dimer, *n* = 62; HbA1c, *n* = 23; ferritin, *n* = 18; troponin I, *n* = 21. Missing data in control patients: D-dimer, *n* = 15; HbA1c, *n* = 5; ferritin, *n* = 5; troponin I, *n* = 4. CCS, Canadian Cardiovascular Society; estimated glomerular filtration rate (eGFR) was estimated using the Chronic Kidney Disease Epidemiology equation^39^; TIA, transient ischemic attack. In the control group, the Abbott Architect CMIA SARS-CoV-2 IgG assay^δ^ was used to confirm absence of prior infection with COVID-19. The primary outcome evaluation (visit 2) was scheduled 28–60 days post-discharge.

^a^Categorical data are summarized as frequency and percentage and compared between groups using Fisher’s exact tests. Continuous data are summarized as mean and standard deviation or median and IQR (defined as the upper and lower quartiles) and compared between groups using Kruskal–Wallis tests. All *P* values are two-sided. No adjustments were made for multiple comparisons.

### Comparison with controls

Twenty-nine control patients with similar age, sex, ethnicity and cardiovascular risk factors underwent the same research procedures during a single visit between 13 April and 2 July 2021. Their characteristics are described in Table 1. Compared to controls, patients with COVID-19 had multisystem differences in keeping with acute illness at enrollment.

### Healthcare workers

Thirty-six (23%) individuals were healthcare workers. Compared to non-healthcare workers, healthcare workers were younger (mean age (s.d.), 51 (9) years versus 55 (13) years; *P* = 0.013) and were more often female (26 (72.2%) versus 43 (35.0%); *P* < 0.001) and from non-White ethnic backgrounds (8 (22.2%) versus 12 (9.8%); *P* = 0.043). They had a lower 10-year percentage cardiovascular risk (%) (8.1 (7.9) versus 14.9 (11.4); *P* = 0.004) and a lower Charlson Comorbidity Index (1.4 (1.6) versus 2.0 (1.9); *P* = 0.030).

### Multisystem investigations: comparisons with controls

In patients hospitalized with COVID-19, compared to controls, the heart, lung and kidney imaging, electrocardiography and multisystem biomarkers revealed several persisting abnormalities (Table 2).

**Table 2:** Multisystem phenotypying: serial electrocardiography, biomarkers of inflammation, metabolism, renal function and hemostasis and heart, lung, and kidney imaging at 28–60 days post-discharge

				Myocarditis				
	COVID-19 *n* = 159	Controls *n* = 29	*P* value	Not present *n* = 17 (11%)	Unlikely *n* = 56 (35%)	Probable *n* = 65 (41%)	Very likely *n* = 21 (13%)	*P* value^a^
ECG *n* (%), admission (*n* = 150)								
**Myopericarditis criteria**	**31 (20.7)**	**0 (0)**	**0.003**	3 (17.6)	9 (16.7)	13 (22.4)	6 (28.6)	0.646
**Premature ventricular contraction**	3 (1.9)	0 (0)	1.000	**1 (5.9)**	**0 (0.0)**	**0 (0.0)**	**2 (9.5)**	**0.013**
ECG *n* (%), enrollment (*n* = 147)								
**Myopericarditis criteria**	**47 (32.0)**	**0 (0)**	**<0.001**	3 (21.4)	16 (30.2)	20 (32.3)	8 (44.4)	0.586
Premature ventricular contraction	1 (0.6)	0 (0)	1.000	0 (0.0)	1 (1.8)	0 (0.0)	0 (0.0)	0.591
ECG *n* (%), 28–60 days post-discharge (*n* = 143)								
**Myopericarditis criteria**	**33 (23.1)**	**0 (0)**	**0.002**	2 (14.3)	10 (20.4)	14 (23.3)	7 (35.0)	0.546
Premature ventricular contraction	2 (1.3)	0 (0)	1.000	1 (5.9)	0 (0.0)	1 (1.5)	0 (0.0)	0.220
CT chest 28–60 days post-discharge								
**Ground glass opacity and/or consolidation,** ***n*** **(%)**	**70 (44.6)**	**1 (4.2)**	**<0.001**	10 (66.7)	26 (46.4)	24 (36.9)	10 (47.6)	0.201
**Reticulation and/or architectural distortion,** ***n*** **(%)**	**47 (29.9)**	**1 (4.2)**	**0.006**	6 (40.0)	15 (26.8)	18 (27.7)	8 (38.1)	0.600
Pulmonary arterial thrombus, *n* (%)	5 (3.3)	0 (0.0)	1.000	0 (0.0)	2 (3.6)	2 (3.1)	1 (5.3)	0.905
**Visual estimate of % of total lung area abnormal, mean (s.d.)**	**14.3 (19.0)**	**0.1 (0.5)**	**<0.001**	19.3 (22.5)	12.7 (17.6)	12.3 (17.5)	21.1 (23.4)	0.286
CT coronary angiogram 28–60 days post-discharge								
FFR_CT,_ patient-level (all coronary arteries)								
**Minimum FFR**_**CT**_**, mean (s.d.)**	**0.80 (0.10)**	**0.84 (0.09)**	**0.006**	0.82 (0.08)	0.79 (0.11)	0.81 (0.09)	0.76 (0.13)	0.541
Cardiovascular MRI 28–60 days post-discharge								
LV end-diastolic volume index, mean (s.d.), ml/m^2^	75.9 (17.0)	73.9 (18.7)	0.326	77.2 (17.9)	74.1 (16.6)	77.4 (17.1)	75.2 (17.7)	0.881
**LV end-systolic volume index, mean (s.d.), ml/m**^**2**^	**35.3 (12.8)**	**30.2 (13.7)**	**0.012**	34.6 (11.1)	33.7 (11.7)	36.3 (14.2)	36.6 (12.4)	0.815
**LV ejection fraction, mean (s.d.), %**	**54.1 (9.7)**	**60.4 (9.3)**	**<0.001**	54.8 (9.8)	55.1 (10.1)	54.0 (8.6)	51.3 (11.5)	0.433
LV ejection fraction reduced, males <48%, *n* (%)	19 (21.3)	2 (12.5)	0.518	2 (15.4)	6 (17.1)	8 (24.2)	3 (33.3)	0.665
**LV ejection fraction reduced, females** < **51%,** ***n*** **(%)**	12 (17.6)	0 (0.0)	0.346	**1 (25.0)**	**0 (0.0)**	**6 (18.8)**	**5 (41.7)**	**0.012**
**LV mass index, mean (s.d.), g/m**^**2**^	**91.8 (25.6)**	**119.4 (26.8)**	**<0.001**	100.9 (18.9)	93.2 (21.5)	89.7 (27.8)	87.6 (31.6)	0.170
**RV end-diastolic volume index, mean (s.d.), ml/m**^**2**^	**73.3 (17.7)**	**79.7 (14.1)**	**0.019**	77.8 (18.7)	72.7 (19.7)	72.6 (17.0)	73.3 (13.9)	0.760
RV end-systolic volume index, mean (s.d.), ml/m^2^	35.9 (11.3)	34.4 (10.0)	0.948	34.6 (12.4)	36.6 (11.9)	35.0 (11.5)	38.1 (8.3)	0.487
**RV ejection fraction, mean (s.d.), %**	**50.9 (10.5)**	**58.5 (9.3)**	**<0.001**	54.6 (15.9)	49.9 (9.5)	51.9 (9.0)	47.5 (11.4)	0.210
Myocardial tissue characterization								
Increased global T1 (>1,233 ms), *n* (%)	55 (34.8)	5 (19.2)	0.174	**2 (12.5)**	**14 (25.0)**	**31 (46.2)**	**9 (42.9)**	**0.016**
Increased global T2 (>44 ms), *n* (%)	10 (6.3)	0 (0.0)	0.312	**0 (0.0)**	**0 (0.0)**	**6 (9.2)**	**4 (19.0)**	**0.007**
T2 ratio (myocardium/serratus anterior muscle)	1.7 (0.2)	1.6 (0.1)	0.180	**1.6 (0.2)**	**1.6 (0.2)**	**1.8 (0.2)**	**1.8 (0.3)**	**<0.001**
**Increased global extracellular volume (>27.4%),** ***n*** **(%)**	**71 (49.7)**	**5 (20.8)**	**0.014**	**1 (7.7)**	**22 (41.5)**	**35 (60.3)**	**13 (68.4)**	**<0.001**
Late gadolinium enhancement								
Myocardial late gadolinium enhancement, *n* (%)	30 (19.0)	2 (7.7)	0.262	4 (25.0)	7 (12.5)	13 (20.0)	6 (28.6)	0.329
Ischemic distribution, *n* (%)	8 (5.5)	0 (0.0)	0.658	0 (0.0)	2 (3.9)	5 (8.1)	1 (5.6)	0.769
**Non-ischemic distribution,** ***n*** **(%)**	24 (16.3)	2 (8.0)	0.606	**4 (28.6)**	**5 (9.8)**	**8 (12.9)**	**7 (35.0)**	**0.033**
Pericardial thickening, *n* (%)	33 (21.2)	0 (0.0)	0.176	1 (5.9)	10 (18.5)	15 (23.4)	7 (33.3)	0.197
Myocardial inflammation (Lake Louise criteria), *n* (%)								
**Probable (1/2)**	**74 (46.8)**	**1 (3.4)**	**<0.001**	**4 (25.0)**	**49 (87.5)**	**21 (32.3)**	**0 (0.0)**	**<0.001**
**Definite (2/2)**	**67 (42.4)**	**1 (3.4)**	**<0.001**	**0 (0)**	**2 (3.6)**	**44 (67.7)**	**21 (100.0)**	**<0.001**
Renal MRI, mean (s.d.)								
Average cortex T1 of right and left kidneys, ms	1,544 (62)	1,519 (70)	0.118	1,548 (66)	1,535 (58)	1,541 (63)	1,585 (60)	0.110
**Average medulla T1 of right and left kidneys, ms**	1,934 (69)	1,953 (59)	0.161	**1,935 (66)**	**1,924 (65)**	**1,925 (66)**	**2,008 (57)**	**0.003**
**Average T1 corticomedullary differentiation of kidneys**	**0.80 (0.03)**	**0.78 (0.03)**	**<0.001**	0.80 (0.03)	0.79 (0.03)	0.80 (0.03)	0.79 (0.02)	0.535
Biomarkers at enrollment, central laboratory								
**eGFR, median (IQR), ml/min/1.73** **m**^**2**^	**96 (85, 106)**	**89 (70, 98)**	**0.025**	95 (88, 103)	94 (84, 107)	97 (83, 105)	98 (94, 104)	0.931
**C-reactive protein, median (IQR), mg/L**	**5.5 (1.6, 22.3)**	**1.5 (0.8, 3.5)**	**<0.001**	6.0 (1.6, 18.2)	5.5 (1.4, 22.8)	4.9 (1.8, 21.6)	14.0 (0.9, 21.5)	0.971
High-sensitivity troponin I, median (IQR), ng/L	3 (2, 6)	4 (4, 6)	0.059	4 (3, 5)	3 (2, 7)	3 (2, 5)	4 (3, 7)	0.609
**NT-proBNP, median (IQR), pg/ml**	**114 (57, 262)**	**58 (38, 99)**	**0.004**	108 (57, 246)	116 (65, 258)	93 (49, 278)	139 (65, 274)	0.546
**Ferritin, median (IQR), µg/L**	**366 (202, 675)**	**186 (96, 254)**	**<0.001**	428 (143, 576)	398 (281, 658)	313 (172, 683)	379 (187, 637)	0.529
**Haptoglobin, median (IQR), g/L**	**2.1 (1.3, 3.1)**	**1.5 (1.2, 1.8)**	**0.001**	2.2 (1.8, 3.2)	2.0 (1.5, 2.8)	1.9 (1.2, 3.1)	2.6 (1.6, 3.2)	0.738
**Prothrombin time, mean (s.d.), s**	12.1 (3.7)	11.2 (0.8)	0.106	**12.1 (2.0)**	**12.7 (5.5)**	**11.7 (2.4)**	**12.0 (1.5)**	**0.042**
**D-dimer, mean (s.d.), ng/ml**	**259 (221)**	**152 (149)**	**<0.001**	290 (195)	263 (178)	246 (168)	260 (192)	0.374
**Fibrinogen, mean (s.d.), g/L**	**4.1 (1.7)**	**3.2 (1.1)**	**0.006**	3.9 (1.5)	4.1 (1.7)	4.0 (1.6)	4.5 (1.9)	0.659
**Factor VIII, mean (s.d.), IU/dL**	**184 (94)**	**99 (39)**	**<0.001**	208 (88)	183 (97)	181 (99)	173 (73)	0.527
**VWF:GP1bR, mean (s.d.), IU/dL**	**236 (127)**	**137 (70)**	**<0.001**	257 (176)	241 (118)	224 (123)	246 (122)	0.755
**VWF:Ag, mean (s.d.), IU/dL**	**243 (145)**	**204 (251)**	**0.002**	310 (235)	233 (118)	228(135)	261 (140)	0.380
Biomarkers at 28–60 days post-discharge, central laboratory								
**eGFR, median (IQR), ml/min/1.73** **m**^**2**^	**95 (83, 106)**	**88 (70, 98)**	**0.047**	91 (79, 103)	95 (82, 106)	95 (87, 105)	98 (79, 105)	0.954
C-reactive protein, median (IQR), mg/L	1.9 (0.9, 3.5)	1.5 (0.8, 3.5)	0.572	1.7 (1.1, 3.5)	2.0 (1.2, 3.4)	1.8 (0.8, 4.4)	1.9 (0.9, 3.1)	0.996
**High-sensitivity troponin I, median (IQR), ng/L**	**2 (1, 4)**	**4 (4, 6)**	**<0.001**	2 (2, 4)	2 (1, 5)	2 (1, 4)	2 (1, 4)	0.941
**NT-proBNP, median (IQR), pg/ml**	**83 (54, 198)**	**58 (38, 99)**	**0.022**	60 (30, 172)	112 (65, 207)	87 (56, 148)	75 (52, 213)	0.290
Ferritin, median (IQR), ug/L	144 (72, 282)	186 (96, 254)	0.529	145 (86, 299)	158 (94, 296)	129 (57, 206)	157 (99, 319)	0.360
**Haptoglobin, median (IQR), g/L**	**1.3 (0.9, 1.6)**	**1.5 (1.2, 1.8)**	**0.031**	1.4 (1.1, 1.6)	1.2 (0.8, 1.6)	1.3 (0.8, 1.6)	1.3 (0.9, 1.8)	0.709
D-dimer, mean (s.d.), ng/ml	205 (252)	152 (149)	0.146	171 (111)	207 (200)	194 (193)	266 (517)	0.965
Fibrinogen, mean (s.d.), g/L	3.4(1.4)	3.2 (1.1)	0.439	3.6 (2.4)	3.2 (0.9)	3.5 (1.3)	3.8 (1.7)	0.468
**Factor VIII, mean (s.d.), IU/dL**	**149 (65)**	**108 (58)**	**<0.001**	151 (96)	141 (54)	151 (70)	160 (50)	0.606
VWF:GP1bR, mean (s.d.), IU/dL	143 (80)	137 (70)	0.912	138 (104)	136 (76)	144 (79)	165 (77)	0.345
VWF:Ag, mean (s.d.), IU/dL	164 (97)	204 (251)	0.479	151 (79)	155 (85)	157 (88)	224 (144)	0.091
Urine biomarkers								
Albumin:creatinine ratio at 28–60 days post-discharge, mean (s.d.)	3.8 (10.9)	6.2 (26.9)	0.257	5.1 (13.4)	5.1 (15.2)	3.4 (5.4)	3.8 (6.4)	0.900

An expanded version is provided in Supplementary Table 2.

Missing data in the COVID-19 (admission, enrollment, 28–60 days) and control groups: myopericarditis criteria: *n* = 9, *n* = 12, *n* = 16, *n* = 0. Missing data in patients after COVID-19 at 28–60 days and controls: CT chest atelectasis, reticulation, ground glass: *n* = 2, *n* = 5; pulmonary arterial thrombus: *n* = 8, *n* = 6; CT coronary angiogram 28–60 days and controls: Agatston score: *n* = 7, *n* = 4; CAD-RADS score: *n* = 5, *n* = 4; FFR_CT_: *n* = 27, *n* = 4; cardiovascular MRI 28–60 days post-discharge: left ventricular end-diastolic volume index, left ventricular end-systolic volume index, left ventricular ejection fraction, left ventricular strain: *n* = 2, *n* = 3; left ventricular mass: *n* = 2, *n* = 3; right ventricular end-diastolic volume index, right ventricular systolic volume index: *n* = 4, *n* = 3; right ventricular ejection fraction: *n* = 3, *n* = 3; global T1: *n* = 1, *n* = 3; global T2: *n* = 1, *n* = 3; global extracellular volume: *n* = 16, *n* = 5; late gadolinium enhancement: *n* = 1, *n* = 3; ischemic distribution: *n* = 14, *n* = 4; non-ischemic distribution: *n* = 12, *n* = 4; mixed distribution: *n* = 14, *n* = 4; pericardial thickening: *n* = 3, *n* = 3; pericardial effusion: *n* = 2, *n* = 3; right and left atrial area: *n* = 1, *n* = 3; myocardial inflammation: *n* = 1, *n* = 0. Missing data for blood biomarkers in the COVID-19 (enrollment and 28–60 days) and control groups: eGFR: *n* = 9, *n* = 10, *n* = 8; C-reactive protein: *n* = 4, *n* = 6, *n* = 2; high-sensitivity troponin I: *n* = 6, *n* = 8, *n* = 2; ΝΤ-proBNP: *n* = 6, *n* = 10, *n* = 2; total cholesterol, triglycerides, HDL cholesterol: *n* = 4, *n* = 6, *n* = 2; fibrinogen: *n* = 5, *n* = 10, *n* = 2; D-dimer: *n* = 5, *n* = 9, *n* = 2; Factor VIII: *n* = 5, *n* = 9, *n* = 2; antithrombin: *n* = 5, *n* = 10, *n* = 3; protein C: *n* = 5, *n* = 10, *n* = 3; protein S: *n* = 3, *n* = 11, *n* = 3; VWF:GP1bR: *n* = 6, *n* = 9, *n* = 2; VWF:Ag: *n* = 5, *n* = 9, *n* = 2. aPTT, activated partial thromboplastin time; CAD-RADS, Coronary Artery Disease-Reporting and Data System; ECV, extracellular volume; eGFR (CKD-EPI), estimated glomerular filtration rate using the Chronic Kidney Disease Epidemiology equation^39^; EF, ejection fraction; EDV, end-diastolic volume; ESV, end-systolic volume; FFR_CT_, fractional flow reserve computed tomography; HbA1c, hemoglobin A1c; HDL, high-density lipoprotein; LV, left ventricle; MESA, Multi-Ethnic Study of Atherosclerosis; NT-proBNP, N-terminal pro B-type natriuretic peptide; PT, prothrombin time; RV, right ventricle; T1, longitudinal relaxation time; T2, transverse relaxation time; TCT, thrombin clotting time; vWF:Ag, von Willebrand factor antigen.

^a^Categorical data are summarized as frequency and percentage and compared between groups using Fisher’s exact tests. Continuous data are summarized as mean and standard deviation or median and IQR (defined as the upper and lower quartiles) and compared between groups using Kruskal–Wallis tests. All *P* values are two-sided. No adjustments were made for multiple comparisons. The Kendall’s tau rank correlation between Lake Louise criteria and the final adjudication (four levels) was 0.75 or, with two levels, was 0.72, representing moderately strong correlations.

At 28–60 days post-discharge (visit 2), computed tomography (CT) chest abnormalities were common. In the post-COVID-19 group, the minimum patient-level fractional flow reserve computed tomography (FFR_CT_) was lower than in the control group, consistent with more flow-limiting coronary artery disease. MRI revealed mild differences in ventricular function, and one in five patients had evidence of myocardial fibrosis revealed by late gadolinium enhancement. Renal MRI findings were similar between the COVID-19 and control groups.

Circulating concentrations of C-reactive protein, ferritin, D-dimers, fibrinogen, Factor VIII and von Willebrand factor were higher in the post-COVID-19 group at enrollment compared to the control group, consistent with hemostatic pathway activation. At 28–60 days post-discharge, Factor VIII concentration remained high. Circulating concentrations of N-terminal pro B-type natriuretic peptide (NT-proBNP) were higher in the COVID-19 group at enrollment and 28–60 days post-discharge.

### Primary outcome

The likelihood of myocarditis was adjudicated by consensus (Methods) as ‘very likely’ in 21 (13%) patients, ‘probable’ in 65 (41%) patients, ‘unlikely’ in 56 (35%) patients and ‘not present’ in 17 (11%) patients. Adjudicated likelihood of myocarditis was associated with typical radiological features of COVID-19 (*P* = 0.024), intensive care admission (*P* = 0.048) and invasive ventilation (*P* = 0.004), but there were no associations with demographic characteristics, cardiovascular history or standard care blood results obtained during the index hospitalization (Table 1).

Assigning an ordinal scale of values from 1 to 4 for the adjudicated likelihood of myocarditis, the total variance across all ratings was 0.885. The variance between adjudicated ratings was 0.725. The ratio of the between-patient variation to the total variation was 0.82, consistent with a high degree of reliability in the median ratings. Each rater re-assessed *n* = 30 cases in a blinded manner to assess intra-observer variability. The average weighted kappa statistic for classifying the likelihood of myocarditis into four levels was 0.69 and, for the binary classification (probable/very likely versus not present/unlikely), was 0.79.

### Multisystem phenotyping and adjudicated myocarditis

#### Electrocardiology

Premature ventricular contractions associated with the likelihood of myocarditis (Table 2).

#### CT chest, coronary and pulmonary angiography

Myocarditis was associated with the distribution of coronary atherosclerosis (Coronary Artery Disease-Reporting and Data System (CADS-RADS) score; *P* = 0.013) (Supplementary Table 2) but no other CT findings at 28–60 days.

#### Cardiovascular MRI

**Extended Data Fig. 7:**
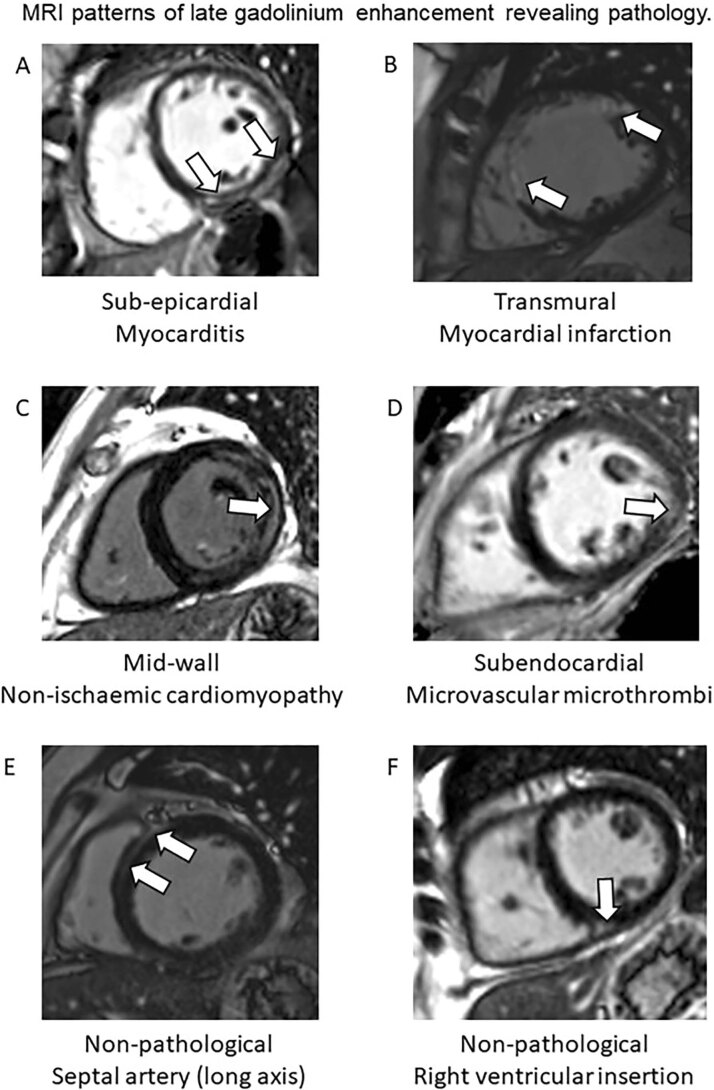
Case examples of predefined patterns of myocardial late gadolinium enhancement. The white arrows indicate late gadolinium enhancement.

The adjudicated likelihood of myocarditis was associated with reduced left ventricular ejection fraction in females (Table 2). Distinct patterns of myocardial pathology were revealed by late gadolinium enhancement imaging illustrated in Extended Data Fig. 7.

#### Renal MRI

The adjudicated likelihood of myocarditis was associated with acute kidney injury (AKI) during the initial admission. At 28–60 days, average renal medulla T1 (ms), an imaging marker of inflammation in the left and right kidneys, was associated with adjudicated myocarditis (*P* = 0.003).

### Etiology of myocarditis

The etiology of myocardial inflammation was also adjudicated. SARS-COV-2 myocarditis was determined as being probable in 14 (66.7%) patients or very likely in seven (33.3%) patients with adjudicated myocarditis (*P* < 0.001) (Supplementary Table 4). Impaired myocardial blood flow as a stressor of inflammation was determined as probable or very likely in six (9.3%) patients with myocarditis adjudicated to be either probable or very likely (*P* < 0.001).

### Multivariable associates of myocarditis

**Extended Data Fig. 8:**
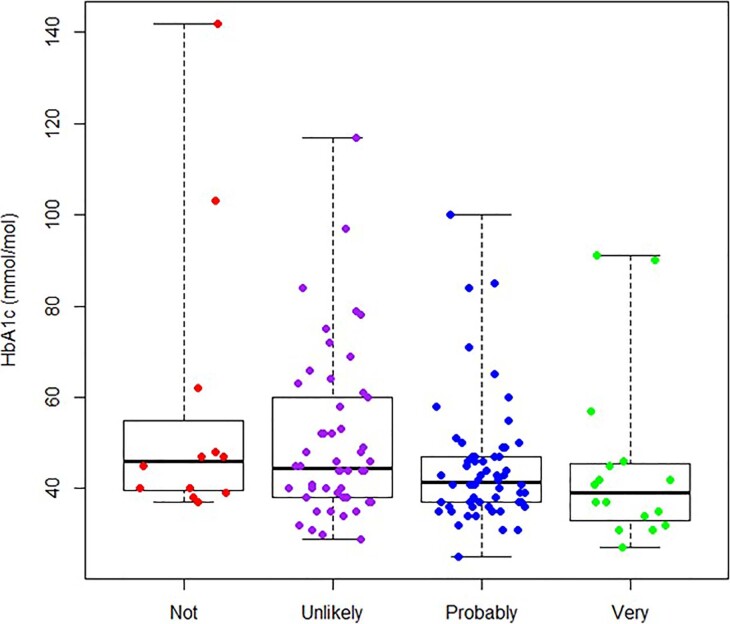
Glycated hemoglobin (HbA1c) (mmol/mol) (n = 136) during the index hospitalization in relation to the adjudicated likelihood of myocarditis (p = 0.10). The boxplot indicates the minimum and maximum values (whiskers), the sample median (middle value), and the first and third quartiles (25th and 75th percentiles, bounds of the box).

**Extended Data Fig. 9:**
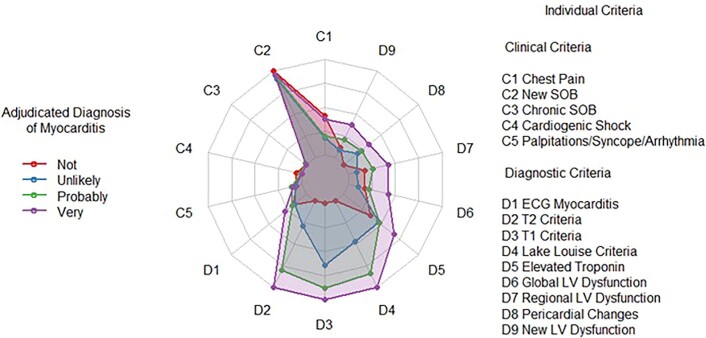
The radar plot illustrates the characteristics of patients in terms of the presence of each individual criterion, separately for those with each adjudicated likelihood of myocarditis. A point in the center of the plot (for example, criteria D2, D3, and D4, for those patients adjudicated as not having myocarditis) indicates that the criterion was absent for all patients in this group. A point on the outer rim of the plot indicates that the criterion was present for all patients in that subgroup. The only exception to this is for Lake Louise criteria (criterion D4), which was coded as 0, 1, or 2, depending on the number of criteria present, so a point on the outer rim indicates all patients in the subgroup had both Lake Louise criteria. The colored regions identify the associations between each likelihood classification and potential diagnostic criteria. The diagnostic test criteria discriminate well between ‘Very likely’ and ‘Probable’, whereas this is not the case for Clinical Criteria, which are very much less specific.

Univariate and multivariable associations between selected demographic and clinical measures at enrollment (visit 1) and an adjudication of myocarditis being probable or very likely were assessed with logistic regression models (Table 3). Healthcare worker status (odds ratio, 95% confidence interval: 2.99 (1.01, 8.89); *P* = 0.048), AKI (3.26 (1.00, 10.64); *P* = 0.050) and HbA1c (per standard deviation increase, on a logarithmic scale) (0.64 (0.42, 0.99); *P* = 0.044) were multivariable associates of adjudicated myocarditis. The inverse association between HbA1c (mmol/mol) and the adjudicated likelihood of myocarditis is illustrated in Extended Data Fig. 8. The concordance between raters for the adjudicated myocarditis diagnosis is shown in Supplementary Table 5. The data illustrate a high level of concordance between the raters for myocarditis not present and good discrimination between probable and very likely. The associations for the clinical and diagnostic test criteria for myocarditis and the adjudicated diagnosis are shown in a radar plot (Extended Data Fig. 9) and in Supplementary Table 6.

**Table 3:** Univariate and multivariable associates of adjudicated myocarditis (primary outcome), including demographic characteristics (A), cardiovascular history (B), severity of COVID-19 (C) and biomarkers (D)

	Univariate		Multivariable	
	Odds ratio (95% CI)	*P* value	Odds ratio (95% CI)	*P* value
Demographics				
Age (per 10 years)	0.87 (0.67, 1.14)	0.304	1.02 (0.72, 1.45)	0.897
**Sex (Female vs. Male)**	**2.01 (1.06, 3.82)**	**0.033**	1.45 (0.64, 3.26)	0.372
Ethnicity (Other vs. White)	2.17 (0.79, 5.98)	0.133		
SIMD (Quintile 2 vs. Most Deprived)	0.49 (0.20, 1.21)	0.120		
SIMD (Quintile 3 vs. Most Deprived)	0.41 (0.14, 1.21)	0.108		
SIMD (Quintile 4 vs. Most Deprived)	0.58 (0.20, 1.70)	0.319		
SIMD (Quintile 5 vs. Most Deprived)	1.10 (0.43, 2.81)	0.838		
**Healthcare worker (Yes vs. No)**	**2.31 (1.05, 5.10)**	**0.038**	**2.99 (1.01, 8.89)**	**0.048**
Body mass index (per 5 kg/m^2^)	1.11 (0.89, 1.39)	0.364		
Cardiovascular history				
Hypertension (Yes vs. No)	0.69 (0.36, 1.33)	0.274		
Chronic kidney disease (Yes vs. No)	2.19 (0.41, 11.65)	0.357		
Diabetes (Yes vs. No)	0.65 (0.31, 1.38)	0.262		
Hypercholesterolemia (Yes vs. No)	0.59 (0.32, 1.11)	0.105		
Smoking (Former vs. Never)	0.98 (0.48, 1.98)	0.950		
Smoking (Current vs. Never)	3.12 (0.62, 15.74)	0.167		
History of cardiovascular disease (Yes vs. No)	0.73 (0.39, 1.37)	0.335		
Q-Risk 3 10-year cardiovascular risk (per 10%)	0.83 (0.60, 1.15)	0.258		
Medical history				
Charlson Comorbidity Index (per point)	0.95 (0.80, 1.12)	0.524		
ISARIC4C in-hospital mortality risk (per 10%)	0.81 (0.60, 1.09)	0.161		
WHO score (oxygen therapy vs. hospitalized, no oxygen)	0.63 (0.31, 1.31)	0.215		
WHO score (non-invasive ventilation vs. hospitalized, no oxygen)	0.55 (0.19, 1.55)	0.256		
WHO score (invasive ventilation vs. hospitalized, no oxygen)	1.83 (0.51, 6.57)	0.352		
**AKI (Yes vs. No)**	**3.26 (1.11, 9.53)**	**0.031**	**3.26 (1.00, 10.64)**	**0.050**
Biomarkers (standard care)				
Hemoglobin (per s.d.)	0.99 (0.73, 1.36)	0.973		
Platelet count (per s.d., log scale)	1.26 (0.92, 1.71)	0.145		
Peak white cell count (per s.d., log scale)	1.15 (0.85, 1.55)	0.369		
Lowest lymphocyte count (per s.d., log scale)	1.38 (0.98, 1.95)	0.063		
Peak D-dimer (per s.d., log scale)	1.01 (0.68, 1.52)	0.947		
Peak fibrinogen (per s.d.)	1.99 (0.72, 5.48)	0.182		
**Peak HbA1c (per s.d., log scale)**	**0.66 (0.46, 0.94)**	**0.023**	**0.64 (0.42, 0.99)**	**0.044**
Peak creatinine (per s.d., log scale)	1.15 (0.87, 1.54)	0.324		
Peak ferritin (per s.d., log scale)	0.86 (0.61, 1.23)	0.416		
Peak high-sensitivity troponin I (per s.d., log scale)	1.23 (0.90, 1.66)	0.190		
Peak C-reactive protein (per s.d., log scale)	0.76 (0.51, 1.14)	0.182		

Odds ratios, 95% confidence intervals and *P* values derived from logistic regression models. Univariate models include one predictor only. Multivariable model was adjusted for age and sex and included any other factors found to have *P* < 0.05 in univariate analysis (that is, healthcare worker status, AKI and HbA1c). For each predictor, the odds ratio relates to the specified between-group difference (categorical predictors) or increase (continuous predictors). CI, confidence interval. All *P* values are two-sided. No adjustments were made for multiple comparisons.

### Health status

Compared to controls, at enrollment and 28–60 days post-discharge, patients who had COVID-19 had lower health-related quality of life, enhanced illness perception, higher levels of anxiety and depression, lower levels of physical activity and lower predicted maximal oxygen utilization (ml/kg/min) (Table 4).

**Table 4:** Health status, illness perception, anxiety and depression and physical function

					Myocarditis				
	Patients, *n*	COVID-19 *n* = 159	Controls n = 29	*P* value	Not present *n* = 17 (11%)	Unlikely *n* = 56 (35%)	Probable *n* = 65 (41%)	Very likely *n* = 21 (13%)	*P* value
Health status, mean (s.d.)									
**Health-related quality of life EQ-5D-5L score at enrollment**	**153**	**0.74 (0.22)**	**0.87 (0.20)**	**0.003**	0.80 (0.19)	0.78 (0.18)	0.72 (0.24)	0.66 (0.25)	0.145
**Health-related quality of life EQ-5D-5L score 28–60** **days post-discharge**	**156**	**0.77 (0.23)**	**0.87 (0.20)**	**0.006**	**0.85 (0.13)**	**0.81 (0.20)**	**0.75 (0.27)**	**0.64 (0.20)**	**0.005**
**Patient-assessed EQ-5D-5L score at enrollment, EQ-5D-5L score**	**153**	**61.5 (21.9)**	**74.9 (19.6)**	**0.001**	71.2 (18.7)	64.2 (19.0)	57.0 (23.0)	59.9 (25.8)	0.094
Patient-assessed EQ-5D-5L score at 28–60 days post-discharge,	157	72.6 (19.6)	74.9 (19.6)	0.429	75.3 (16.6)	74.3 (17.3)	73.0 (21.3)	63.0 (20.9)	0.126
Illness perception, mean (s.d.)									
**Brief Illness Perception Questionnaire score at enrollment**	**152**	**42.4 (12.3)**	**33.9 (14.8)**	**0.003**	37.8 (12.0)	42.1 (11.3)	42.8 (12.7)	45.2 (13.4)	0.464
**Brief Illness Perception Questionnaire score 28–60 days post-discharge**	157	36.5 (14.7)	**33.9 (14.8)**	**0.215**	**33.2 (12.2)**	**35.9 (14.3)**	**35.1 (15.6)**	**45.8 (11.5)**	**0.022**
Anxiety and depression, mean (s.d.)									
**PHQ-4 anxiety score at enrollment**	**152**	**2.13 (2.08)**	**0.79 (1.59)**	**<0.001**	1.53 (1.74)	1.83 (1.83)	2.37 (2.24)	2.70 (2.63)	0.309
**PHQ-4 anxiety score at 28–60 days post-discharge**	**154**	**1.81 (2.00)**	**0.79 (1.59)**	**0.003**	1.20 (1.08)	1.43 (1.73)	2.10 (2.24)	2.45 (2.24)	0.197
**PHQ-4 depression score at enrollment**	**152**	**2.19 (1.95)**	**0.70 (1.51)**	**0.002**	1.59 (1.87)	2.06 (1.79)	2.34 (2.02)	2.60 (2.16)	0.388
**PHQ-4 depression score at 28–60 days post-discharge**	**154**	**1.78(1.90)**	**0.70 (1.51)**	**<0.001**	**1.07 (1.10)**	**1.34 (1.68)**	**2.10 (2.07)**	**2.55 (2.06)**	**0.028**
**PHQ-4 total score at enrollment**	**152**	**4.32 (3.78)**	**1.28 (2.67)**	**<0.001**	3.12 (3.37)	3.89 (3.29)	4.71 (4.03)	5.30 (4.35)	0.313
**PHQ-4 total score at 28–60 days post-discharge**	**154**	**3.59 (3.71)**	**1.28 (2.67)**	**<0.001**	2.27 (2.02)	2.77 (3.11)	4.19 (4.20)	5.00 (3.97)	0.051
Physical function									
**IPAQ category at enrollment,** ***n*** **(%)**	140								
**High**		**12 (8.6)**	**12 (42.9)**	**<0.001**	2 (11.8)	3 (5.9)	4 (7.4)	3 (16.7)	0.448
**Moderate**		**16 (11.4)**	**7 (25.0)**		3 (17.6)	6 (11.8)	7 (13.0)	0 (0.0)	
**Low**		**112 (80.0)**	**9 (32.1)**		12 (70.6)	42 (82.4)	43 (79.6)	15 (83.3)	
**IPAQ category at 28-60 days post-discharge,** ***n*** **(%)**	131								
**High**		**19 (14.5)**	**12 (32.1)**	**<0.001**	4 (33.3)	4 (8.2)	9 (17.6)	2 (10.5)	0.176
**Moderate**		**44 (33.6)**	**7 (25.0)**		5 (41.7)	18 (36.7)	17 (33.3)	4 (21.1)	
**Low**		**68 (51.9)**	**9 (32.1)**		3 (25.0)	27 (55.1)	25 (49.0)	13 (68.4)	
**Duke Activity Status Index at enrollment**	**148**	**19.6 (18.0)**	**46.3 (18.5)**	**<0.001**	25.7 (18.5)	19.9 (17.7)	17.9 (17.7)	19.2 (19.9)	0.246
**Duke Activity Status Index at 28–60 days post-discharge**	**157**	**24.2 (17.6)**	**46.3 (18.5)**	**<0.001**	**33.6 (18.7)**	**25.1 (17.9)**	**23.9 (17.4)**	**14.6 (12.5)**	**0.014**
**Predicted maximal oxygen** **utilization (ml/kg/min) at enrollment**	**148**	**18.0 (7.8)**	**29.5 (8.0)**	**<0.001**	20.6 (8.0)	18.1 (7.6)	17.3 (7.6)	17.8 (8.6)	0.246
**Predicted maximal oxygen utilization (ml/kg/min) at 28–60 days post-discharge**	**157**	**20.0 (7.6)**	**29.5 (8.0)**	**<0.001**	**24.0 (8.0)**	**20.4 (7.7)**	**19.9 (7.5)**	**15.9 (5.4)**	**0.014**

PHQ-4, Patient Health Questionnaire-4; IPAQ, International Physical Activity Questionnaire. Categorical data are summarized as frequency and percentage and compared between groups using Fisher’s exact tests. Continuous data are summarized as mean and standard deviation and compared between groups using Kruskal–Wallis tests. All *P* values are two-sided. No adjustments were made for multiple comparisons.

The adjudicated likelihood of myocarditis associated with patient-reported outcome measures at 28–60 days post-discharge, including lower health-related quality of life (*P* = 0.005), enhanced illness perception (*P* = 0.022), enhanced depression score (*P* = 0.028), lower physical activity (*P* = 0.014) and lower predicted maximal oxygen utilization (ml/kg/min) (*P* = 0.014).

### Serious adverse events

Follow-up was continued to 13 December 2021 for all participants. The mean (s.d., range) duration of follow-up after hospital discharge was 450 (88) days (range, 290–627 days). The serious adverse events (SAEs) occurring during the index admission and the adjudicated causes of death and readmission post-discharge are detailed in Supplementary Tables 7–9.

Four patients died during the study period, including two deaths before visit 2 and two deaths after visit 2. Twenty-four (15.1%) patients post-COVID-19 and two (4.7%) control patients died or were rehospitalized (*P* = 0.356). One hundred eight (67.9%) patients who had COVID-19 and seven (25.9%) controls had an episode of outpatient secondary care (*P* = 0.017), and more patients who had COVID-19 were referred for symptoms consistent with NICE188 guideline criteria^21^ for Long COVID-19 (58 (36.5%) versus 1 (3.7%); *P* = 0.017). The adjudicated likelihood of myocarditis was associated with a diagnosis of pulmonary fibrosis (*P* < 0.001). Prescribed medications during follow-up are described in Supplementary Table 10.

## Discussion

We investigated multisystem pathology coupled with patient-reported health status, aerobic exercise capacity and clinical outcomes during a 14-month period after hospitalization for COVID-19. One in seven patients died or were readmitted to hospital, and two-thirds had an episode of outpatient secondary care.

Our results bridge a knowledge gap between post-COVID-19 syndromes and objective evidence of disease. We found evidence of persisting multisystem cardio-renal injury, including increased circulating concentrations of NT-proBNP, a biomarker of impaired cardiac function and prognosis^22^, and Factor VIII, reflecting hemostasis pathway activation. These abnormalities partly explain the lingering impairments in patient-reported health-related quality of life, physical function and psychological well-being. Taken together, our findings implicate multisystem injury pathways as mediators of post-COVID-19 syndrome.

Incident myocarditis persisting 28–60 days post-COVID-19 affected approximately one in eight (13%) patients, which is lower than reports from previous studies (27–60%)^19,20^. The etiology of myocarditis was predominately SARS-CoV-2 infection and, less commonly, myocardial ischemia due to coronary artery disease (Supplementary Table 4). The clinical significance of myocarditis complicating COVID-19 is highlighted by associations with pulmonary fibrosis diagnosed during follow-up.

Myocardial scar tissue reduces heart pump function, and, in the general population, myocardial scar tissue confers an adverse prognosis.^23^ In our post-COVID-19 cohort, distinct from controls, myocardial scar was a surprisingly common finding, affecting one in five patients. Radiological features of myocardial scar patterns are discriminatory and indicative of etiology and acuteness. In our cohort, the fibrosis distribution revealed distinct etiologies of acute myocardial injury, including myocarditis, microvascular thrombosis and myocardial infarction. The imaging features also identified pre-existing fibrosis with a non-ischemic pattern (Extended Data Fig. 7). The prognostic implications of these findings should be clarified through longitudinal follow-up studies.

**Extended Data Fig. 10:**
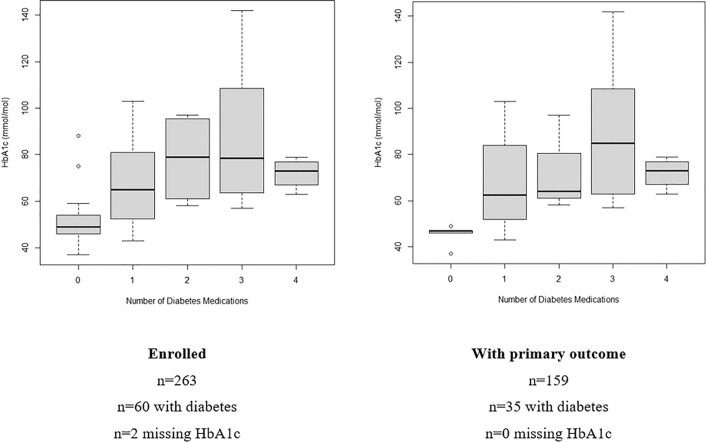
Glycated hemoglobin (HbA1c) (mmol/mol) during the index hospitalization in relation to the number of prescribed anti-diabetic medications. Anti-diabetic medications prescribed (n, patients): biguanide n = 36; sodium-glucose transport protein 2 (SGLT2) inhibitor n = 21; insulin n = 14; sulfonylurea n = 12; glucagon-like peptide-1 (GLP-1) receptor agonist n = 6; dipeptidyl peptidase-4 (DPP-4) inhibitors n = 6; thiazolidinedione n = 3. Boxplots show median, quartiles, and range of non-outlying data (outliers defined as those more than 1.5 times the interquartile range above the upper quartile or below the lower quartile). The boxplot indicates the minimum and maximum values (whiskers), the sample median (middle value), and the first and third quartiles (25th and 75th percentiles, bounds of the box).

Hemoglobin A1c (%) was associated with adjudicated myocarditis but in the opposite direction to what may be expected and so requiring validation in other cohorts. The mechanism may involve systemic inflammation leading to microangiopathic hemolytic anemia and reduced red cell survival^24^, although the lack of association with haptoglobin (Table 2) and other hematological parameters does not support this possibility in our population. Hemoglobin A1c (%) was positively associated with the number of anti-diabetic medications (Extended Data Fig. 10), implying more intensive medical therapy. Reverse causality may also be relevant. For example, if ‘fit’ individuals with a low HbA1c are eventually admitted to hospital, then they have pronounced COVID-19 illness and so a greater myocardial ‘hit’, whereas individuals with cardiovascular risk factors and pre-existing cardiovascular morbidity (and a higher HbA1c) have less reserve (or buffering capacity) to tolerate illness and are hospitalized with relatively milder COVID-19 illness.

AKI portends mortality in COVID-19 (refs. ^11,25^). Adjudicated myocarditis was associated with AKI during admission and the imaging evidence of inflammation in the kidney medulla 28–60 days post-discharge. These associations might be explained by systemic injury pathways—that is, inflammation, hemostasis activation, microvascular dysfunction and persisting COVID-19 infection—or a combination of these pathologies^11^. Considering clinical translation, the results support a stratified management approach for patients who had post-COVID-19 early during convalescence. Biomarkers, such as NT-proBNP, could be used by clinicians to guide risk stratification of patients for more intensive medical management and rehabilitation during convalescence.

Almost one-quarter of the patients who had COVID-19 were healthcare workers, and this employment status was a multivariable associate of the adjudicated likelihood of myocarditis with a three-fold-higher odds ratio. Healthcare workers were younger, more often female and of non-White ethnicity and had fewer cardiovascular risk factors and comorbid conditions. Reverse causality may be relevant in that individuals with reasonably good background health have greater reserve to withstand COVID-19, and, in those who eventually need hospital care, the illness is more severe, including complications such as myocarditis. A second factor could be enhanced exposure to SARS-CoV-2 in that some healthcare workers are repeatedly exposed to sources of infection in their workplace, potentially leading to a greater viral load on exposure. This hypothesis merits further investigation.

Post-COVID-19 syndrome (‘Long COVID’) predominately affects women^1,6,13,26^. The proportion of women increased with the likelihood of myocarditis, and female sex was a univariable associate of adjudicated myocarditis, which, in turn, was associated with lower mental and physical well-being. Adjudicated myocarditis was associated with left ventricular systolic dysfunction in women. Our findings provide a pathophysiological basis for physical limitation in some female patients after COVID-19 (ref. ^26^).

Troponin elevation represents a diagnostic criterion for myocarditis^27^. However, circulating troponin concentrations may increase due to hypoxia, hypotension, ischemia and renal failure as well as from localized myocardial injury. Troponin elevation lacks diagnostic specificity, and this leads to uncertainties in clinical practice. The clinical assessment of patients presenting with COVID-19 and chest symptoms should include a detailed history, examination (heart rate, rhythm, blood pressure and auscultation) and a 12-lead electrocardiogram (ECG)^28^. If there are cardiac findings, then blood biomarkers—for example, high-sensitivity troponin assay—should be measured, and, if abnormal, imaging—for, example, echocardiography—becomes warranted if the findings would lead to a change in management. Cardiac MRI should be considered when positively discriminating cardiac findings—for example, pericardial chest pain, saddle-shaped ST elevation on the ECG and ventricular arrhythmias—support the likelihood of myocarditis^28^ Our findings support cardiac screening in patients who have experienced AKI after COVID-19. Referral for diagnostic procedures should be balanced against the risk of infection transmission to staff. Our study should inform clinical guideline updates for the integrated care of patients with persisting symptoms after COVID-19 (refs. ^21,29^).

Although there are no evidence-based treatments for post-COVID-19 syndromes, acute treatments, such as dexamethasone^30^, should reduce the likelihood of myocarditis occurring. Our findings identify cardio-renal involvement as a candidate endpoint in clinical trials aimed at preventing post-COVID-19 syndrome. Evidence of hemostasis pathway activation provides a pathophysiological correlate for the beneficial effects of antithrombotic therapy in hospitalized populations^31,32^. The RECOVERY trial is investigating the effects of immunomodulatory therapies, such as baricitinib, and the sodium–glucose co-transporter-2 inhibitor empagliflozin, which has beneficial effects in patients with type 2 diabetes at high cardiovascular risk^33^. Data from the UK Office for National Statistics indicate that individuals who have had two doses of vaccine have ~41.1% lower odds of self-reported Long-COVID symptoms^34^. The effect of vaccination on illness trajectory in the longer term merits investigation.

To our knowledge, the multisystem protocol involving simultaneous heart, lung and renal imaging has not been implemented previously. Coronary angiography with FFR_CT_ provided a high level of certainty for identifying flow-limiting coronary artery disease. This is relevant because pre-existing coronary artery disease giving rise to ischemia is a confounding cause of myocardial inflammation.

Our study was designed to minimize selection bias. Use of hospital-level electronic health records in real time facilitated an unbiased, prospective screening approach. Troponin elevation was not an eligibility criterion, and renal dysfunction was not an exclusion criterion. Our study design stands apart from previous studies that involved selected populations (COVID-HEART^35^ and COVIDsortium^36^), retrospective case selection^19,20^ or a sample size limiting generalizable conclusions^12^. In a cardiac screening study of 789 North American professional athletes who had tested positive for COVID-19, cardiac abnormalities were uncommon (3.8%) and myo-pericarditis was identified in 0.6% of individuals, without adverse cardiac events^37^. For community-dwelling, healthy, young individuals post-COVID-19, these results are reassuring.

Our study minimized ascertainment bias, which may have affected previous studies of myocarditis. The diagnosis of each patient was independently adjudicated by a committee of cardiologists, and the statistical analysis was undertaken by biostatisticians independent of the research team. Given that the study involved a central laboratory approach, measurement variations were minimized. Electronic health records were used for follow-up assessments, and there were no missing data.

To minimize COVID-19 transmission to our staff during the study, imaging was scheduled from 28 days post-discharge. This approach aligns with the International Severe Acute Respiratory and Emerging Infection Coronavirus Clinical Characterisation Consortium (ISARIC4C) study^38^. Because acute imaging was not performed, some pathologies may have resolved by 28 days. Most of the patients in our cohort were unvaccinated during enrollment. The definition of AKI was based on in-hospital blood tests. Endomyocardial biopsy was not performed. Selection and ascertainment bias were minimized but not eliminated, and patients who were deemed too frail to comply with the study procedures were not enrolled.

## Conclusions

The illness trajectory of COVID-19 includes persisting cardio-renal inflammation, lung involvement, hemostatic pathway activation and impairments in physical and psychological function. One in seven patients died or were rehospitalized, and two in three patients had additional outpatient episodes of secondary care, considerably higher than controls. Preventive therapy for post-COVID-19 syndromes and longer-term studies of prognosis are warranted.

## Methods

### Design

This study involved a prospective, observational, multicenter, longitudinal, secondary care cohort design to assess the time course of multi-organ injury in survivors of COVID-19 during convalescence (ClinicalTrials.gov ID NCT04403607)^40^. Clinical information, a 12-lead digital ECG, blood and urine biomarkers and patient-reported outcome measures were acquired at enrollment (visit 1) and again during convalescence, 28–60 days post-discharge (visit 2). Chest CT, including pulmonary and coronary angiography, and cardio-renal MRI were acquired at the second visit.

The Scottish Index of Multiple Deprivation (SIMD) is a small-area measure of social deprivation based on seven factors (income, employment, education, health, access to services, crime and housing) and categorized into general population quintiles. The SIMD was used to measure social deprivation^41^.

### Setting

This study involved three hospitals in the West of Scotland (population, 2.2 million): the Queen Elizabeth University Hospital, the Glasgow Royal Infirmary and the Royal Alexandra Hospital in Paisley.

### Participant identification

Patients who received hospital care for COVID-19, with or without admission, and were alive, were prospectively screened in real time using an electronic healthcare information system (TrakCare, InterSystems) and daily hospital reports identifying inpatients with laboratory-positive results for COVID-19.

### Eligibility criteria

The inclusion criteria were: (1) age ≥18 years; (2) history of an unplanned hospital visit—for example, emergency department or hospitalization >24 hours for COVID-19 confirmed by a laboratory test (for example, polymerase chain reaction (PCR)); (3) ability to comply with study procedures; and (4) ability to provide written informed consent. The imaging results were reported by accredited radiologists according to contemporary national guidelines^42^.

The exclusion criteria were: (1) contraindication to MRI (for example, severe claustrophobia or metallic foreign body) and (2) lack of informed consent.

#### Screening

A screening log was prospectively completed. The reasons for being ineligible, including lack of inclusion criteria and/or presence of exclusion criteria, were recorded.

### Diagnosis of COVID-19

A diagnosis of COVID-19 was based on laboratory evidence of SARS-CoV-2 infection using a PCR test (Roche Cobas 6800 or Seegene SARS-CoV-2 PCR) on a biospecimen^43^. The laboratory tests were either the Roche Cobas 6800 or Seegene SARS-CoV-2 PCR tests.

### Control group

The study design included a contemporary control group of at least 20 individuals who would undergo the same research procedures using the same scanners and core laboratory methods. The control group was designed to closely represent the characteristics of the study population, including recent episodes of secondary care where possible.

In August 2020, an interim analysis of the COVID-19 participants’ characteristics was undertaken to define the enrollment criteria for the control group.

- *n* = 41 patients
- Mean (s.d.) age: 55 (11) years
- Sex: 53% male, 47% female
- Cardiovascular risk factors were prevalent.

#### Eligibility criteria—inclusion

1. Age range, 40–80 years
2. At least one cardiovascular risk factor by ASSIGN criteria: http://www.assign-score.com/estimate-the-risk/risk-factors/#more-info
  Age >65 years
  SIMD Quintiles 1 or 2
  Family history of coronary heart disease or stroke
  Diabetes
  Rheumatoid arthritis
  Cigarette smoker
  Systolic hypertension (ASSIGN criteria) or history of treated hypertension
  Hyperlipidemia (ASSIGN criteria) or history of treated hyperlipidemia
  Body mass index ≥30 kg m^−2^

#### Eligibility criteria—exclusion

Prior history of:

- Myocardial infarction
- Myocarditis
- Heart failure
- Structural heart disease
- Positive serology for COVID-19
- History of COVID-19

#### Screening approach for controls

The medical research staff screened the electronic health records of patients under their care, or personal contacts, with known episodes of care in primary or secondary care. The screening approach excluded patients with a prior history of COVID-19 infection. Before the research visit, a blood test for COVID-19 serology (Abbott Architect CMIA SARS-CoV-2 IgG assay) was used to confirm the absence of prior infection with COVID-19. A negative result was required to proceed with the research visit. All of the controls had negative serology tests for COVID-19.

### Diagnosis of myocardial injury

The diagnosis of myocardial injury aligned with the Fourth Universal Definition of Myocardial Infarction^44^. Troponin I was measured in hospitalized patients using the Abbott Architect STAT TnI assay (sex-specific >99th percentile upper reference limit: female: >16 ng L^−1^, male: >34 ng L^−1^). Serial blood sampling was undertaken to detect temporal changes in the circulating concentration of cardiac troponin to classify acute versus chronic myocardial injury.

### Diagnosis of AKI

AKI was defined as any stage of AKI (1–3) during COVID-19 hospitalization using categorization with the Kidney Disease: Improving Global Outcomes (KDIGO) criteria^45^.

### Research schedule

The protocol involved two visits. The first visit involved informed consent and assessments during the initial hospitalization or as soon as possible after discharge. The second visit occurred 28–60 days post-discharge. This window was positioned to reflect the convalescent phase and give sufficient scope to schedule the patients.

The procedures involved prospective collection of clinical data and a time course of research investigations. Clinical data included demographics, medical and cardiovascular history, findings from clinical examinations, laboratory and radiological tests, cardiology tests (including an ECG and an echocardiogram if available) and treatment. The research investigations at both visits included blood and urine samples, a 12-lead digital ECG (BeneHeart R3, Mindray) and health status questionnaires. Heart, lung and kidney imaging were acquired at the second visit.

### Electrocardiology

SARS-CoV-2 infection and treatment may cause alterations in heart rate and rhythm and ventricular repolarization. The changes may be specific for myocarditis (for example, concave ST elevation) or non-specific (for example, ventricular arrhythmias). Digital ECGs were acquired, de-identified and provided to the University of Glasgow Electrocardiology Core Laboratory for automated analysis and adjudication. The ECG features of myopericarditis were predefined according to contemporary criteria^18^.

Digital ECGs were recorded using a Mindray BeneHeart R3 electrocardiograph, which was supplied to the participating centers. A standard 10-second, 12-lead ECG, sampled at 500 samples per second, was obtained when possible with this device. On occasions, particularly for ECG recording in the emergency department, a standard hospital ECG was acquired. These ECGs were transmitted to a central ECG management system (GE Muse) and, hence, were available for visual review. Up to three ECGs per patient were available, consisting of the ECG soon after admission as well as ECGs obtained at the first and second research visits, as defined earlier.

The ECGs from the R3 electrocardiograph were transferred securely to the study portal at the University of Glasgow and then downloaded to the ECG Core Laboratory at Glasgow Royal Infirmary. These ECGs were interpreted using the University of Glasgow ECG analysis software and visually reviewed. Each ECG was assessed by two reviewers acting together. All interpretative findings were transferred to a spreadsheet for statistical analysis, with particular attention being paid to ST-T changes and serial changes in sequentially acquired ECGs.

An automated interpretation of myocarditis was not available. Hence, this ECG diagnosis was based on a combination of automated ECG analysis, expert review by core laboratory staff (P.M. and R.S.) and predefined features according to contemporary guidelines^18^.

### Biomarkers

To investigate the mechanisms of cardiovascular injury arising from SARS-CoV-2 infection, blood and urine samples were collected at enrollment (visit 1) and 28–60 days post discharge (visit 2).

Blood samples collected into 0.109 M sodium citrate (for hemostasis assays) or EDTA (for other biomarkers) were handled according to a sample handling manual, which was provided to all sites. The blood samples were centrifuged locally, and the plasma was separated and frozen at −80 °C within 2 hours of sampling. Residual samples were transferred to the NHS Glasgow Biorepository for storage at the end of the study.

Circulating biomarkers of cardiac injury (troponin I, NT-proBNP), inflammation (C-reactive protein, ferritin), thrombosis (TCT ratio, D-dimer, fibrinogen, Factor VIII, antithrombin, protein C, protein S), endothelial activation (von Willebrand factor (vWF):GP1bR, VWF:Ag) and renal function (serum creatinine; glomerular filtration rate (GFR), estimated using the Chronic Kidney Disease Epidemiology (CKD-EPI) equation^39^; and urinary albumin:creatinine ratio) and their changes over time were investigated. The measurements were undertaken in central laboratories, blinded to the other clinical data.

EDTA plasma samples were stored at −80 °C in the Glasgow Biorepository until batch analysis at the end of the study. The biochemical analyses were performed in the British Heart Foundation Glasgow Cardiovascular Research Centre. EDTA plasma samples were stored to analyze high-sensitivity cardiac troponin I and NT-proBNP on first thaw. Troponin I (ng ml^−1^) and NT-proBNP (pg ml^−1^) were measured in blood samples collected at visit 1 and visit 2. NT-proBNP (pg ml^−1^) was measured to provide a biochemical measurement of left ventricular remodeling (within-patient change in NT-proBNP at follow-up from baseline)^46^ and troponin I to provide a biochemical measurement of myocardial necrosis.

For measurement of both NT-proBNP and high-sensitivity cardiac troponin I, we used an automated method (i1000SR ARCHITECT, Abbott Diagnostics), calibrated and quality controlled using the manufacturer’s reagents. We also participated in the National External Quality Assurance Scheme (NEQAS). The limit of detection of troponin I is 0.0012 ng ml^−1^, and the 99th percentile value in a healthy subpopulation is 0.0262 ng ml^−1^. The between-assay coefficient of variations were 3.7% and 7.1% for control materials with mean troponin I concentrations of 15.43 ng ml^−1^ and 0.015 ng ml^−1^, respectively.

For NT-proBNP, the coefficient of variation was 3.6% and 5.5% for control materials with mean NT-proBNP level of 5141 pg ml^−1^ and 139 pg ml^−1^, respectively. The troponin I and NT-proBNP results were provided to the Robertson Centre for Biostatistics at the University of Glasgow.

#### Hemostasis markers

##### Sample handling

All sodium citrate plasma samples were processed in a non-standard manner using anonymized barcoded samples by a trained member of staff within the Glasgow Biorepository. Frozen plasma samples were subsequently transported on dry ice for central laboratory analysis in the Department of Haematology at Glasgow Royal Infirmary. This laboratory is accredited by the United Kingdom Accreditation Service. Plasma samples were stored at −80 °C until analysis, with residual samples being transferred to the Glasgow Biorepository for storage at the end of the study.

#### Assays

All hemostasis laboratory assays were carried out using Werfen reagents on Werfen ACL TOP 550/750 or Werfen ACL AcuStar (VWF:GP1bR only) analyzers, in accordance with the manufacturer’s guidelines using a single lot of Werfen reagent. The coagulation screen consisted of a prothrombin time (PT) assay, activated partial prothrombin time (APTT) assay, thrombin clotting time (TCT) assay and fibrinogen Clauss assay with normal reference ranges of 9–13 seconds, 27–36 seconds, 11–15 seconds and 1.7–4 g L^−1^, respectively (all internally derived). The fibrin D-dimer assay (latex immunoassay) had a reference range <230 ng ml^−1^ (manufacturer derived). The one-stage FVIII assay was carried out using SynthASil reagent (Werfen) and had a range of 58–152 IU dl^−1^. The VWF:Ag (latex immunoassay) and VWF:GP1bR activity assay (chemiluminescent immunoassay) had reference ranges of 51–170 IU dl^−1^ and 52–172 IU dl^−1^, respectively (internally derived). Antithrombin activity (chromogenic), free-protein S (latex immunoassay) and protein C activity assay (chromogenic) had reference ranges of 82–123 IU dl^−1^, 75–137 IU dl^−1^ and 71–146 IU dl^−1^, respectively (all internally derived). Hemostasis laboratory assays were completed after the fulfilment of internal quality control checks using control material traceable to International Standards, in accordance with standard laboratory operating procedures. Furthermore, all methodology used for the purposes of this study is regularly subject to external quality control checks through the internationally recognized scheme, UKNEQAS. The laboratory results were provided directly to the Robertson Centre for Biostatistics at the University of Glasgow.

### Multimodality imaging

#### Overview

CT is the reference method for imaging the chest, and CT coronary and pulmonary angiography are the reference techniques for imaging the coronary arteries and pulmonary circulation, respectively. Cardiovascular MRI is recommended for imaging myocardial injury. Cardio-renal MRI was undertaken at a single reference site: the Imaging Centre of Excellence, Queen Elizabeth University Hospital, University of Glasgow. The study was designed to minimize measurement variation that might arise during imaging acquisition and analysis. All patients were imaged on the same research-dedicated MRI and CT scanners rather than on different hospital service scanners. All patients were imaged 28–60 days post-discharge. The rationale for undertaking the MRI at this time point was to assess for persisting evidence of cardio-renal injury in the convalescent phase, when the risk of infection transmission to staff was minimal.

### CT

A 320-detector CT scanner (Aquilion ONE, Canon Medical Systems) provided full heart coverage within a single heartbeat. Intravenous metoprolol was used where required to control the heart rate (target, 60 beats per minute (bpm)), and sublingual glyceryl trinitrate was given to all patients immediately before the scan acquisition. An initial low-radiation-dose helical scan of the thorax was acquired for comprehensive assessment of the lungs. A contrast bolus timing scan was acquired to provide information on cardiopulmonary transit times. Non-contrast and contrast-enhanced angiographic breath-hold ECG-gated volumes were acquired and timed for optimum pulmonary and systemic arterial (coronary) opacification. Patients with severe renal dysfunction underwent non-contrast CT.

Coronary CT angiography provided information on the presence and extent of coronary calcification (calcium score), coronary artery disease and whether any coronary artery disease was obstructive (flow-limiting), including the Coronary Artery Disease-Reporting and Data System (CAD-RADS) score^47^. The functional significance of coronary artery disease was evaluated using fractional flow reserve CT (FFR_CT_; HeartFlow). An FFR_CT_ ≤ 0.80 defined obstructive coronary artery disease, taking the lowest value in the vessel. FFR_CT_ measurements were taken at prespecified points using standard coronary segment definitions as a reference^48^. Median FFR_CT_ values were calculated for the left anterior descending, circumflex and right coronary arteries, respectively, in combination with subsidiary vessels (that is, diagonal arteries and obtuse marginal arteries). Patient-level FFR_CT_ values included all these coronary arteries.

Pulmonary vascular imaging assessed arterial thrombus (embolism)^49^. CT was used to delineate pulmonary features associated with COVID infection—for example, atelectasis, reticulation and/or architectural distortion, ground glass opacity and pre-existing lung damage—for example, emphysema. Cardiac and extra-cardiac incidental findings were reported and managed according to local standards of care.

### Cardiovascular MRI acquisition

Patients were scanned using a clinical research-dedicated 3.0 Tesla (3T) MRI scanner (MAGNETOM Prisma, Siemens Healthineers) with two 18-channel surface coils placed anteriorly and a 32-channel spine coil placed posteriorly in the convalescent phase.

Balanced steady-state free precession (SSFP) sequences were used to acquire ventricular cine imaging in three long axis planes, followed by a short axis stack from the apex to the atrio-ventricular ring, each with 30 phases. Images were obtained using retrospective ECG gating at end-expiration. Typical scan parameters were: field of view (FOV), 340 × 286 mm; slice thickness, 7 mm, with 3-mm gap in short axis stack; repetition time (TR), 41.4 ms; echo time (TE), 1.51 ms; flip angle, 50°; and voxel size, 1.33 × 1.33 × 7 mm.

Three left ventricular short axis (basal, mid and apical) and one orthogonal long axis longitudinal relaxation time (T1, spin–lattice relaxation time constant in milliseconds) motion-corrected, optimized, modified Look-Locker inversion recovery sequences^50^ were acquired with the following typical parameters: FOV, 360 × 306 mm; slice thickness, 8.0 mm; voxel size, 1.9 × 1.9 × 8.0 mm; TR, 264 ms; TE, 1.12 ms; flip angle, 35°; minimum T1, 100 ms; inversion time increment, 80 ms; and bandwidth, 1,085 Hz per pixel.

A short axis stack of transverse relaxation time (T2, spin–spin relaxation time constant in milliseconds) maps and orthogonal long axis views were acquired, followed by an automated exponential fit for each pixel after respiratory motion correction. The imaging used a T2-prepared single-shot SSFP readout with T2 preparation times (TE) = 0, 25 and 55 ms, with a recovery period of three heartbeats between measurements. Typical protocol parameters for T2 mapping were: FOV, 360 × 270 mm; slice thickness, 8 mm; matrix, 192 × 116; spatial resolution, 1.9 × 1.9 mm; TR, 207.39 ms; TE, 1.32 ms; flip angle, 12°; and bandwidth, 1,184 Hz per pixel.

Late gadolinium enhancement images, including three long axis acquisitions and a short axis stack, were acquired 10–15 minutes after intravenous injection of 0.15 mmol kg^−1^ of gadolinium diethyltriaminepenta-acetic acid (Gd-DTPA, Magnevist, Bayer Healthcare) using segmented phase-sensitive inversion recovery turbo fast low-angle shot. Typical imaging parameters were: matrix, 192 × 111; flip angle, 14°; TE, 1.05 ms; bandwidth, 1,085 Hz per pixel; echo spacing, 2.1 ms; and trigger pulse, 1 ms. The voxel size was 1.9 × 1.9 × 7 mm^3^. Inversion times were individually adjusted to optimize nulling of visually normal myocardium (typical values, 250–350 ms).

Three left ventricular short axis (basal, mid and apical) and orthogonal long axis T1 motion-corrected, optimized, modified Look-Locker inversion recovery sequences were acquired 15 minutes after contrast administration with the following typical parameters: FOV, 360 × 306 mm; slice thickness, 8.0 mm; voxel size, 1.9 × 1.9 × 8.0 mm; TR, 341 ms; TE, 1.01 ms; flip angle, 35°; minimum T1, 100 ms; inversion time increment, 80 ms; and bandwidth, 1,085 Hz per pixel.

### Cardiovascular MRI analysis

The cardiovascular MRI scans were reviewed and reported by an accredited radiologist (G.R. with >15 years of image analysis experience). A single image analyst (K.M. with >8 years of image analysis experience) analyzed all data, which were subsequently reviewed by C.B. (with > 15 years of image analysis experience).

#### Reference ranges

Contemporary, local reference ranges were derived using the 3T MRI scanner (MAGNETOM Prisma, Siemens Healthineers) by A.M. and K.M. as part of standard quality assurance in the University of Glasgow Clinical Imaging Research Facility. These scans were acquired during the same period as the current study and analyzed using dedicated software (cvi42 software for cardiovascular MRI, version 5.10, Circle Cardiovascular) to derive mean, upper and lower reference ranges. This software package was also used for the cardiovascular MRI analyses of the study participants.

#### Ventricular function

The imaging analyses were performed using dedicated cardiovascular MRI software (cvi42 software (version 5.10, Circle Cardiovascular)). Routinely reported measures of left ventricular and right ventricular function were carried out according to contemporary guidelines^51^. Ventricular endocardial and epicardial contours were manually drawn at end-diastole and end-systole, which was deemed to be the phase with the smallest blood pool cavity. Papillary muscles were excluded from myocardial mass and included in volumes. Global left ventricular strain (circumferential, longitudinal and radial) and global right ventricular strain (longitudinal) were derived using the software’s tissue tracking module to determine peak values for each parameter. Atrial areas were manually drawn on four-chamber horizontal long axis views at atrial diastole (defined with respect to mitral valve closure).

#### Parametric mapping

Motion-corrected T1 and T2 scans were analyzed using dedicated software (cvi42 software (version 5.10, Circle Cardiovascular)). The individual images were reviewed to ensure that motion correction was successful. Parametric maps were generated, and goodness-of-fit (R^2^) was reviewed. Myocardial segments with artifacts that impaired diagnostic quality and/or measurement accuracy, including pixels/segments with R^2^ < 0.99, were excluded from analysis.

Epicardial and endocardial borders were manually drawn, and care was taken to include only myocardial tissue with a 10% epicardial and endocardial offset applied to avoid partial volume effects. The right ventricular insertion points were used to segment the myocardium as per the American Heart Association’s 16-segment left ventricular model^52^. For blood pool pre-contrast and post-contrast T1, regions of interest were drawn within the left ventricular cavity on the three short axis maps, with care taken to avoid artifact and papillary muscles.

Hematocrit values were acquired the day of the study visit. Additional regions of interest were manually drawn on a representative area of serratus anterior, identified from the T2 stack.

#### Late gadolinium enhancement imaging

The archive of late gadolinium enhancement images for each patient was initially qualitatively reviewed for image quality and artifacts. The imaging set included the short axis stack and three or more orthogonal long axis views.

Myocardial late gadolinium patterns were predefined. They included myocarditis, myocardial infarction, non-ischemic cardiomyopathy and microvascular thrombosis. The location of the late gadolinium enhance was defined as sub-endocardial, mid-wall, sup-epicardial or pericardial. Myocardial hyperenhancement in the basal septum was reviewed in association with the cardiac-gated CT image reconstruction, and, if compatible with a septal perforator artery, this feature was excluded from the late gadolinium enhancement analyses. Hyperenhancement at right ventricular insertion points may be observed in individuals without cardiac disease. Therefore, this feature was not defined as pathological.

The full width at half maximum (FWHM) technique was used to evaluate myocardial late gadolinium enhancement imaging based on a literature review by K.M., as this method is reported to be highly reproducible^53,54^ and less conducive to ‘over-reporting’ the extent of late gadolinium enhancement when compared to other methods^54,55^. The FWHM technique is described as the optimal semi-automated quantification method in risk-stratifying patients with suspected myocarditis, demonstrating the strongest association with major adverse cardiac events^54^. Late gadolinium enhancement was reported according to the pattern (distribution) on a per-segment and per-patient basis. The etiological categories for the pattern of late gadolinium enhancement included non-ischemic, ischemic, mixed, micro-thrombi, other or none. Late gadolinium enhancement was quantified as the percentage of left ventricular mass.

### Renal MRI protocol

Multi-parametric renal MRI included anatomical imaging and mapping native T1 and T2. The volume (ml), native T1 (ms) and T2 (ms) in regions of interest obtained within the cortex and medulla of each kidney were recorded, and the averaged values of these parameters for both kidneys were then determined. Corticomedullary differentiation reflects a difference in tissue contrast on T1-weighted imaging due to a shorter T1 relaxation time of the cortex relative to the medulla—this being attributed to differences in water content between the two tissues’ disease^56,57^. Corticomedullary differentiation, reported here as a ratio of T1 cortex divided by T1 medulla^57^, may diminish in kidney disease^56^.

Transverse volumetric interpolated breath-hold examination (VIBE) images, with and without contrast, were acquired for assessment of kidney volume. For T1 and T2 sequences, single oblique coronal slices positioned through the center of both kidneys were acquired with breath held at expiration. In patients where both kidneys could not clearly be included, the right kidney was prioritized.

T1 maps were acquired using a modified Look-Locker inversion recovery (MOLLI) sequence with single-shot true FISP readout. Images were acquired at eight different inversion times (pattern 5(3)3) with a start TI of 180 ms and a TI increment of 80 ms. Motion correction and fitting of the T1 map was performed using a phase-sensitive inversion recovery reconstruction implemented in the vendor software (VE11C, Myomaps, Siemens). Other imaging parameters were: FOV, 360 × 213 mm; slice thickness, 5 mm; matrix, 240 × 190; spatial resolution, 1.5 × 1.5 mm; TE, 1.2 ms; flip angle, 35°; and bandwidth, 1,096 Hz per pixel. The initial T1 protocol used a TR of 550 ms (producing TIs of 130, 210, 680, 760, 1,230, 1,310, 1,780 and 2,330 ms) in error. The preferred T1 mapping sequence had a TR of 1,000 ms to produce a wider range of TIs (130, 210, 1,130, 1,210, 2,130, 2,210, 3,130 and 4,130 ms). Once corrected, all subsequent participants were scanned using TR 550-ms and TR 1,000-ms protocols. Where available, TR 1,000 ms was used preferentially in analysis, but participants with only TR 550-ms images were not excluded.

T2 maps were acquired using a fast low-angle shot (FLASH) inversion recovery gradient echo sequence, with TR, 389 ms; TE, 1.4 ms; preparation pulses, 0, 30 and 55 ms; slice thickness, 5 mm; FOV, 360 × 213 mm; matrix, 240 × 182; and spatial resolution, 1.5 ×1.5 mm.

T1 VIBE images were acquired using FLASH inversion recovery gradient echo sequence with TR, 3.1 ms; TE, 1.22 ms; spectral attenuated inversion recovery (SPAIR) fat saturation; slice thickness, 1.5 mm; FOV, 380 × 308 mm; matrix, 256 × 192; and spatial resolution 1.5 × 1.5 mm.

### Renal MRI analysis

Imaging analysis was performed using a custom ImageJ macro (ImageJ, National Institutes of Health) by K.J.M., P.H.B. and trained physicist colleagues. A thresholding technique was applied to the renal MRI T1 maps to segment the cortex and medulla, creating two regions of interest for T1 (ms), excluding renal pelvis. These regions of interest were overlaid onto the renal MRI T2 maps allowing measurement of T2 (ms). Renal corticomedullary differentiation was calculated by dividing values for the cortex by those of the medulla. Total kidney volume (ml) was determined by manually tracing the kidney on multiple slices to determine area that the software multiplies by slice thickness to determine volume. Overall, the mean values of the measurements for each kidney were taken to represent left and right values.

### Blinding

Blinding measures were predefined to minimize bias. The patients completed the health status questionnaires before undergoing the scans, and they were unaware of the scan results. The assessors of the central laboratory analyses worked independently. Specifically, the radiologists reporting the MRI and CT scans were unaware of the results from the other research procedures—that is, the ECGs, blood biomarkers and questionnaires. In the same way, the researchers undertaking the ECG, FFR_CT_ and laboratory analyses were unaware of the imaging findings. The cardiologist (K.M.) carried out the core-lab quantitative MRI analyses on pseudo-anonymized scans on a dedicated workstation, without access to clinical or other data. The cardiologist who undertook the secondary read (C.B) of the MRI scans was blinded to the study data. The cardiologists who formed the clinical adjudication panel were unaware of the patient-reported outcome measures. They were also unaware of the adjudications made by the other panel members.

### Outcomes

#### Primary outcome

The predefined primary outcome was a diagnosis of myocarditis (myocardial inflammation), a subgroup of acute myocardial injury.

A diagnosis of myocarditis is susceptible to confounding through ascertainment bias. Recent studies in COVID-19 have not implemented the modified Lake Louise diagnostic criteria^19,20^. Accordingly, to limit the potential for bias, we prespecified an adjudication procedure for the primary outcome, involving a panel of experienced cardiologists. The panel reviews were undertaken according to a prespecified charter.

The diagnostic criteria for myocarditis included relevant clinical and diagnostic test criteria^18^. Positive clinical findings included chest pain, pericarditic or pseudo-ischemic in nature; new onset breathlessness; subacute/chronic breathlessness; palpitations; unexplained arrhythmia; syncope; aborted sudden cardiac death; and unexplained cardiogenic shock. Positive test findings included (1) ECG features; (2) elevated troponin I (sex-specific >99th percentile upper reference limit: female: >16 ng L^−1^, male: >34 ng L^−1^; Abbott Architect STAT TnI assay); (3) functional and structural abnormalities on cardiac imaging (echocardiography, angiography or MRI); and (4) tissue characterization MRI, including myocardial edema and late gadolinium enhancement with a distribution in alignment with the modified Lake Louise diagnostic criteria for myocarditis^58^. Acute and chronic myocardial pathology can be identified, discriminated and quantified using MRI.

Myocarditis was clinically suspected if at least one clinical finding and at least one diagnostic test criterion from different categories were observed, in the absence of (1) angiographically detectable coronary artery disease (coronary stenosis ≥50%) and (2) known pre-existing cardiovascular disease or extra-cardiac causes that could explain the syndrome (for example, valve disease, congenital heart disease or hyperthyroidism). Suspicion increases with a rising number of fulfilled criteria. If the patient was asymptomatic, at least two diagnostic criteria were required.

### Adjudication of the primary outcome

A diagnosis of myocarditis is susceptible to confounding through ascertainment bias. Recent studies in COVID-19 have not implemented the modified Lake Louise diagnostic criteria^19,20^. Accordingly, we prespecified an adjudication procedure for the primary outcome, involving a panel of cardiologists with specialty accreditation. The reviews were undertaken according to a prespecified charter.

Consultant cardiologists (*n* = 14) who were independent of the research team were invited as assessors. They were provided with information on the European Society of Cardiology Working Group on Myocardial and Pericardial Disease position statement on myocarditis^18^, a charter and training cases. The cardiologists were blinded to the identity of the patients and independent of their clinical care. The adjudications were coordinated by a researcher (A.M.) using Teams (Microsoft) software.

Each cardiologist independently assessed the clinical data, including the medical history, biomarkers, ECG and radiology reports for the CT chest, CT pulmonary angiogram, coronary CT angiogram and cardiac MRI. De-identified source clinical data (for example, scan images) were made available upon request. The adjudication criteria were ad verbatim transcribed from the European Society of Cardiology Working Group on Myocardial and Pericardial Disease position statement on myocarditis^18^, with clinically suspected myocarditis being indicated if ≥1 clinical presentation and ≥1 diagnostic criteria from different categories (or ≥2 diagnostic criteria in asymptomatic patients), in the absence of >50% stenosis in epicardial coronary arteries, were observed. The ‘clinical presentation’ criteria scored 1 point for acute chest pain (pseudo-ischemic and pericarditic); new onset or worsening breathlessness or fatigue (at rest and on exertion) with or without signs of right or left heart failure; palpitations/syncope/aborted sudden cardiac death; and unexplained cardiogenic shock. The diagnostic criteria include 1 point each for (1) ECG/Holter features, including atrio-ventricular block, bundle branch block and supraventricular or ventricular tachycardias; (2) elevated myocardiocytolysis markers (for example, troponin); (3) functional or structural imaging abnormalities on echocardiography, cardiac MRI or left ventriculography; and (4) tissue characterization by cardiac MRI (modified Lake Louise criteria)^19,20^.

Each cardiologist independently determined the likelihood (not present/unlikely/probable/very likely) of myocardial inflammation (myocarditis) based on the clinical presentation and the summative diagnostic criteria on an individual patient basis. Specifically, the categorization of the likelihood of myocarditis would be informed by the summative score of these criteria, including 1 point for clinical criteria and up to 4 points for diagnostic criteria: ‘not present’ = 0–1, ‘unlikely’ = 2, ‘probable’ = 3 and ‘very likely’ = 4 or more. The adjudication was, therefore, weighted based on specific diagnostic criteria, in line with the clinical guidelines.

Each case was independently adjudicated by five cardiologists. The adjudications for each participant were brought together, and the final diagnosis on a per-participant basis was based on the median likelihood based on the adjudications of five cardiologists. Their determinations were also categorized in binary form (not present/unlikely = no; probable/very likely = yes).

Control cases were also assessed, and the adjudications were undertaken blinded to COVID-19 status. Each rater re-assessed 30 cases to assess intra-observer variability to assess test–retest reliability.

### Secondary outcomes

Myocardial injury was classified by etiology. The potential endotypes were:

1. SARS-CoV-2 myocarditis
2. Acute stress cardiomyopathy
3. Myocardial ischemia/impaired perfusion as a stressor of inflammation
4. Infective myopericarditis (non-COVID infection)
5. Drug-induced (toxic) myocardial inflammation
6. Idiopathic myocardial with or without pericardial inflammation

#### Definitions

The endotypes of acute myocardial injury, including myocardial infarction type according to the 4th Universal Definition of Myocardial Infarction^44^, and myocarditis (myocardial inflammation, ischemia or stress cardiomyopathy)^18,58^ were secondary outcomes.

### Definitions of the secondary outcomes

The definitions align with the guidance from the Task Force for the management of COVID-19 of the European Society of Cardiology^28^, acute myocardial infarction^59,60^, coronary revascularization^61^ and the position statement of the European Society of Cardiology Working Group on Myocardial and Pericardial Diseases^18^.

#### SARS-CoV-2 myocarditis

Diagnostic criteria for clinically suspected viral myocarditis, including clinical presentations and diagnostic criteria, are provided in Supplementary Table 2. Clinically suspected myocarditis was indicated if ≥1 clinical presentation and ≥1 diagnostic criteria from different categories were observed, in the absence of (1) angiographically detectable coronary artery disease (coronary stenosis ≥50%) and (2) known pre-existing cardiovascular disease or extra-cardiac causes that could explain the syndrome (for example, valve disease, congenital heart disease or hyperthyroidism). Suspicion is higher with higher number of fulfilled criteria. If the patient is asymptomatic, ≥2 diagnostic criteria should be met. This criterion was adopted in the knowledge that endomyocardial biopsy for virology criteria would not be available for logistical reasons related to service provision during the pandemic.

#### Acute stress cardiomyopathy

Takotsubo syndrome: myocardial injury secondary to myocardial disorders without involvement of the coronary arteries. Takotsubo syndrome is characterized by specific left ventricular wall motion abnormalities (for example, apical ballooning) and the absence of relevant late gadolinium enhancement with edema^59^. The diagnostic criteria are provided in the International Expert Consensus Document on Takotsubo Syndrome (Part I): Clinical Characteristics, Diagnostic Criteria, and ^62^Pathophysiology:

1. Patients show transient left ventricular dysfunction (hypokinesia, akinesia or dyskinesia) presenting as apical ballooning or midventricular, basal or focal wall motion abnormalities. Right ventricular involvement can be present. Besides these regional wall motion patterns, transitions between all types can exist. The regional wall motion abnormality usually extends beyond a single epicardial vascular distribution; however, rare cases can exist where the regional wall motion abnormality is present in the subtended myocardial territory of a single coronary artery (focal Takotsubo syndrome).
2. An emotional, physical or combined trigger can precede the Takotsubo syndrome event, but this is not obligatory.
3. Neurologic disorders (for example, subarachnoid hemorrhage, stroke/transient ischemic attack or seizures) as well as pheochromocytoma may serve as triggers for Takotsubo syndrome.
4. New ECG abnormalities are present (ST segment elevation, ST segment depression, T-wave inversion or QTc prolongation); however, rare cases exist without any ECG changes.
5. Levels of cardiac biomarkers (troponin and creatine kinase) are moderately elevated in most cases; substantial elevation of brain natriuretic peptide is common.
6. Obstructive coronary artery disease is not a contradiction in Takotsubo syndrome.
7. Patients have no evidence of infectious myocarditis.
8. Postmenopausal women are predominantly affected.

#### Myocardial ischemia/impaired perfusion as a stressor of inflammation

In this study, we used a FFR_CT_ ≤0.80 (ischemic threshold) in a major coronary artery or an occluded coronary artery revealed by CT coronary angiogram (ESC revascularization guidelines^61^).

#### Infective myopericarditis (non-COVID infection),

See (1) + microbiology diagnosis of a concomitant, non-COVID microbial infection.

#### Drug-induced (toxic) myocardial inflammation,

See (1) + clinical diagnosis of drug toxicity (for example, amphetamine, anthracycline, cocaine or lithium).

#### Idiopathic myocardial with or without pericardial inflammation

See (1) + no obvious precipitant cause.

### Renal outcomes

Renal function was assessed using convalescent eGFR (CKD-EPI^39^) and albuminuria. Multi-parametric renal MRI at 28–60 days provided information on renal parenchymal disease.

### Health status and patient-reported outcome measures

Questionnaires were completed by participants at enrollment (visit 1) and 28–60 days after the last episode of hospital care (visit 2), blinded to the other research data. Self-reported health status was assessed using the generic EuroQOL EQ-5D-5L questionnaire and the Brief Illness Perception Questionnaire (Brief-IPQ)^63,64^. The Patient Health Questionnaire-4 (PHQ–4) was used to assess for anxiety and depressive disorders^65^. The Duke Activity Status Index (DASI) was used to assess predicted maximal oxygen utilization (ml/kg/min), a measure of aerobic capacity, and functional capacity—a higher score reflects greater physical function^66^. The International Physical Activity Questionnaire-Short Form (IPAQ-SF) measures the types and intensity of physical activity and sitting time that people do as part of their daily lives. The score reflects total physical activity in metabolic equivalent minutes per week^67^.

### Longitudinal follow-up for clinical outcomes

Participants were invited to give consent for follow-up assessments of SAEs, including death and rehospitalization and NHS resource use, including procedures, outpatient clinic visits and medication prescriptions. Clinical members of the research team assessed electronic health records without participant contact in line with the protocol and a predefined charter. Cardiovascular and respiratory SAEs were independently reviewed and adjudicated by the clinical event committee. The events were entered into the database coordinated by the clinical trials unit.

### Statistics

The statistical analyses were predefined in a Statistical Analysis Plan. The statistical methods are described in the tables.

### Sample size calculation

The primary outcome was myocarditis (myocardial inflammation), and the primary analysis determined the proportion of patients with the primary outcome by visit 2. The likelihood of myocarditis was determined based on the median likelihood from the clinical adjudication committee. To detect an association between a history of pre-existing cardiovascular disease and incident myocardial inflammation (myocarditis), we assumed a 25% prevalence of prior cardiovascular disease in the study population and the incidence of myocardial inflammation in those with or without prior cardiovascular disease to be 33% and 10%, respectively^68^. To have 80% power to detect this difference, we calculated that 140 participants (35 with cardiac problems, 105 without) with complete data would be required. Anticipating that 10–15% of the participants may have incomplete imaging (for example, artifact or claustrophobia), the target sample size was 160 to complete the imaging visit.

Prespecified subgroup analyses are intended for patients without cardiovascular disease, as defined by the absence of (1) prior cardiovascular disease and (2) obstructive coronary artery disease on CT coronary angiogram. Cardiovascular disease status was prespecified and defined by (1) a prior history of cardiovascular disease and (2) treatment. The associations between the circulating concentrations of mechanistic biomarkers, patient-reported outcome measures and their changes over time and the primary and secondary outcomes were assessed. Missing data are reported. Significance tests with two-sided *P* values are accompanied by confidence intervals for estimated effect sizes and measures of association. The widths of the confidence intervals have not been adjusted for multiplicity. The *P* values for subgroup differences were calculated using Fisher’s exact test and the Kruskal–Wallis test for categorical and continuous data, respectively. *P* values less than 0.05 were considered statistically significant.

### Trial management and timelines

This study was conducted in line with the current Guidelines for Good Clinical Practice in Clinical Trials and STROBE guidelines^69^. A Study Management Group included those individuals responsible for the day-to-day management of the study, including the chief investigator, project manager and representatives from the sponsor and scientific laboratories. The roles of this group included facilitating the progress of the study, ensuring that the protocol was adhered to and taking appropriate action to safeguard participants and the quality of the study itself. Decisions about continuation or termination of the study or substantial amendments to the protocol were the responsibility of the sponsor. The Study Management Group met at weekly intervals from May 2020 to October 2021.

A scientific steering group had overall oversight of the study. This study was designed to be undertaken and reported rapidly in response to the global need for information about COVID-19.

### Ethics

This study was approved by the UK National Research Ethics Service (reference 20/NS/0066).

### Reporting Summary

Further information on research design is available in the Nature Research Reporting Summary linked to this article.

## Online content

Any methods, additional references, Nature Research reporting summaries, source data, extended data, supplementary information, acknowledgements, peer review information; details of author contributions and competing interests; and statements of data and code availability are available at 10.1038/s41591-022-01837-9.

## Supplementary information

Supplementary Information CISCO-19 Investigators and Supplementary Tables

Reporting Summary

## Data Availability

Data requests will be considered by the Steering Group, which includes representatives of the sponsor, the University of Glasgow, senior investigators independent of the research team and the chief investigator. The Steering Group will take account of the scientific rationale, ethics, logistics and resource implications. Data access requests should be initially submitted by email to the chief investigator (C.B., corresponding author). The source data include the de-identified numerical data used for the statistical analyses and de-identified imaging scans (MRI and CT) and ECGs. Data access will be provided through the secure analytical platform of the Robertson Centre for Biostatistics. This secure platform enables access to de-identified data for analytical purposes, without the possibility of removing the data from the server. Requests for transfer of de-identified data (including source imaging scans) will be considered by the Steering Group, and, if approved, a collaboration agreement would be expected. The Steering Group will consider any cost implications, and cost recovery would be expected on a not-for-profit basis.

## Code availability

The statistical code is available online in GitHub: https://github.com/RobertsonCentre/cisco19/blob/main/CISCO19_Paper1_v1.R.
