# Supplementary material for: A multisystem, cardio-renal investigation of post-COVID-19 illness
Source: Nat Med. 2022 May 23;28(6):1303–13. doi: 10.1038/s41591-022-01837-9 (PMC9205780; doi:10.1038/s41591-022-01837-9)
Supplement: Supplementary file 1 — CISCO-19 Investigators and Supplementary Tables [file 41591_2022_1837_MOESM1_ESM.pdf]

nature portfolio

https://doi.org/10.1038/s41591-022-01837-9

Supplementary information

A multisystem, cardio-renal investigation of post-COVID-19 illness

In the format provided by the authors and unedited

Supplement

Title: Post-COVID-19 illness trajectory: a multisystem investigation.

Authors: CISCO-19 investigators.

Reference: NMED-A116731

The CISCO-19 Investigators

Chief Investigator: Colin Berry BSc/MBChB/PhD

Grant applicants

Kenneth Mangion MD/PhD, Catherine Bagot MBBS/MD, Colin Church MBChB/PhD, Christian Delles MD, Antonia Ho MBChB/PhD, David J. Lowe MBChB/MD, Alex McConnachie PhD, Giles Roditi MBChB, Rhian M. Touyz MD/PhD, Naveed Sattar MBChB/PhD, Ryan Wereski MBChB, Sylvia Wright MBChB, Colin Berry BSc/MBChB/PhD.

Site investigators

Queen Elizabeth University Hospital

Kenneth Mangion MBChB/PhD, Pauline Hall Barrientos PhD, Kevin G. Blyth MBChB/PhD, Michael Briscoe MBChB, Colin Church MBChB/PhD, Christian Delles MBChB/PhD, Stephen Dobbin MBChB, Keith Gillis MBChB/PhD, Antonia Ho MBChB/PhD, Anna Kamdar BMedSci, David J. Lowe MBChB/MD, Kaitlin J. Mayne MBChB, Patrick B. Mark MBChB/PhD, Christopher McGinley MBChB, Connor McKee BMedSci/ MBChB, Andrew Morrow MBChB, Oliver Peck MBChB, Alastair J Rankin BSc/MBChB, Giles Roditi MBChB, Claire Rooney MBChB/PhD, Sarah A. Spiers PhD, David Stobo MBChB, Ryan Wereski MBChB, Sylvia Wright MBChB/PhD, Colin Berry BSc/MBChB/PhD.

Royal Alexandra Hospital

Lynn Abel BN, Douglas Grieve MBChB/PhD.

Glasgow Royal Infirmary

Catherine Bagot MBBS/MD, Hannah Bayes MBChB/PhD, Jaclyn Carberry

MBChB/BMedSci, Daniel Doherty MBChB, Ian Fergus BSc, Vivienne B. Gibson PhD, Fraser Goldie MBChB, Laura Knox MBChB, Giles Roditi MBChB, Katherine Scott MBChB, David Stobo MBChB, Varun Sharma MBChB, Robert Sykes MBChB/BMedSci.

Glasgow Clinical Research Facility and Study Management Group

Ammani Brown BN, Andrew Dougherty BN, Kirsty Fallon BN, Lesley Gilmour BN.

Chloe Cowan BN, Lynsey Gillespie PhD, Sharon Kean, Jurgen Van-Melckebeke BSc, Kim Moran-Jones PhD, Debra Stuart PhD, Maureen Travers PhD.

Glasgow Clinical Research Imaging Facility, Institute of Clinical Excellence, Queen Elizabeth University Hospital

Tracey Hopkins DCR/BSc(Hons)/PGd, Laura Dymock BSc(Hons), Evonne McLennan BSc(Hons)/PGc, Rosemary Woodward BSc/PGc, Fiona Savage BSc(Hons)/PGc, Nicola Tynan BSc(Hons)/PGc.

Radiology, NHS Greater Glasgow and Clyde Health Board

Sau Lee Chang MBChB, Mhairi Dupre MBChB, Lindsey Norton MBChB, Liam Peng MBChB, Giles Roditi MBChB, David Stobo MBChB.

Laboratory Medicine and Biorepository, NHS Greater Glasgow and Clyde Health Board

Clare Orange BSc, Rory Gunson PhD.

Medical Physics, NHS Greater Glasgow and Clyde

Sarah Allwood-Spiers PhD, George Bruce BSc, Rosario Gonzalez-Lopez PhD, Pauline Hall

Barrientos PhD, Rebecca Stace BSc.

University of Glasgow

Institute of Cardiovascular and Medical Sciences

Colin Berry MBChB/PhD, Elaine Butler BSc, Christian Delles MBChB/PhD, Jennifer S Lees MBChB/PhD, Kenneth Mangion MD/PhD, Patrick Mark MBChB/PhD, Andrew Morrow MBChB, Naveed Sattar MBChB/PhD, Robert Sykes MBChB/BMedSci, Rhian M. Touyz MD/PhD, Paul Welsh PhD.

Institute of Infection, Immunity and Inflammation

MRC Centre for Virology Research

Antonia Ho MBChB/PhD, Massimo Palmarini PhD.

Institute of Health and Wellbeing

Robertson Centre for Biostatistics

John G.F. Cleland MBChB/PhD, Sharon Kean, Bernard Kelly BSc, Alex McConnachie PhD, Alasdair McIntosh PhD, Dionne Russell BSc, Sarah Weeden PhD.

Electrocardiology Core Laboratory

Peter W. Macfarlane DSc, Louise Inglis BSc(Hons), Jean Watt, Kathryn McLaren, Shahid Latif MAppSci (now deceased).

Departments of Mathematics and Statistics

Nick Hill PhD, Dirk Husmeier PhD, Xiaoyu Luo PhD.

National Institutes of Health

Peter Kellman PhD, Hui Xue PhD.

Heartflow

Amy Collinsworth MS, Sarah Mullen MBT, Campbell Rogers MD.

Clinical Event Committee

Heerajnarain Bulluck MBChB/PhD, David Carrick MBChB/PhD, David Corcoran MBChB/PhD, Iain Findlay MBChB/MD, Ninian N. Lang, MBChB/PhD, Vera Lennie MBChB, Ross McGeoch MD, Sabrina Nordin MBChB/MD, Alexander Payne MBChB, Keith E. Robertson MBChB/PhD, Nicola Ryan MBChB, Gruschen Veldtman MBChB/PhD, Robin P. Weir MBChB/PhD, Stuart Watkins MBChB/MD.

Coordinators – Andrew Morrow MBChB, Robert Sykes MBChB/BMedSci.

Steering Committee

College of Medical, Veterinary and Life Sciences, University of Glasgow - Neil Basu MD/PhD, Iain McInnes MBChB/PhD, Stefan Siebert MD/PhD.

Table S1. Expanded - Clinical characteristics of the population by control vs. COVID-19 and likelihood of adjudicated myocarditis post-COVID-19.

		COVID-19		Control	p-value[(a)]	p-value[(b)]	Adjudicated Myocarditis Diagnosis				p-value
		Without Primary Outcome	With Primary Outcome				Not present	Unlikely	Probable	Very likely	
		n = 104	n = 159	n = 29			n = 17	n = 56	n = 65	n = 21	
Baseline Demographics											
Age, years	Mean (SD)	55.6 (12.3)	54.5 (11.9)	57.3 (9.6)	0.608	0.373	56.9 (11.4)	55.1 (13.3)	53.1 (11.4)	54.9 (10.1)	0.525
Sex	Male	65 (62.5%)	90 (56.6%)	18 (62.1%)	0.371	0.685	13 (76.5%)	35 (62.5%)	33 (50.8%)	9 (42.9%)	0.115
	Female	39 (37.5%)	69 (43.4%)	11 (37.9%)			4 (23.5%)	21 (37.5%)	32 (49.2%)	12 (57.1%)	
Race	Arab	0 (0.0%)	4 (2.5%)	0 (0.0%)	0.315	0.788	0 (0.0%)	2 (3.6%)	1 (1.5%)	1 (4.8%)	0.248
	Black	0 (0.0%)	2 (1.3%)	0 (0.0%)			0 (0.0%)	0 (0.0%)	2 (3.1%)	0 (0.0%)	
	East Asian	1 (1.0%)	4 (2.5%)	0 (0.0%)			0 (0.0%)	2 (3.6%)	2 (3.1%)	0 (0.0%)	
	South Asian	3 (2.9%)	8 (5.0%)	2 (6.9%)			0 (0.0%)	0 (0.0%)	6 (9.2%)	2 (9.5%)	
	West Asian	3 (2.9%)	2 (1.3%)	1 (3.4%)			1 (5.9%)	1 (1.8%)	0 (0.0%)	0 (0.0%)	
	Latin American	0 (0.0%)	0 (0.0%)	0 (0.0%)			0 (0.0%)	0 (0.0%)	0 (0.0%)	0 (0.0%)	
	White	97 (93.3%)	139 (87.4%)	26 (89.7%)			16 (94.1%)	51 (91.1%)	54 (83.1%)	18 (85.7%)	
**Most deprived SIMD quintile**		40 (39.6%)	61 (40.4%)	5 (17.9%)	1.000	**0.032**	4 (25.0%)	20 (37.0%)	25 (41.0%)	12 (60.0%)	0.178
Healthcare Worker		18 (17.3%)	36 (22.6%)	5 (17.9%)	0.350	0.804	1 (5.9%)	10 (17.9%)	18 (27.7%)	7 (33.3%)	0.121
**BMI, kg/m²**	Mean (SD)	32.0 (6.5)	30.5 (7.1)	30.7 (5.0)	**0.020**	0.555	30.9 (5.6)	29.6 (5.8)	31.1 (8.7)	30.6 (6.4)	0.829
Presenting Characteristics											
**Heart Rate, bpm**	Mean (SD)	95.4 (19.7)	94.8 (19.1)	69.0 (15.1)	0.652	**<0.001**	97.7 (18.8)	94.4 (20.2)	94.7 (16.3)	93.6 (25.0)	0.586
**Systolic blood pressure, mmHg**	Mean (SD)	132.7 (18.4)	128.5 (19.8)	142.1 (18.6)	0.085	**0.003**	122.0 (24.5)	134.6 (18.3)	126.3 (19.8)	124.1 (16.6)	0.139
Diastolic blood pressure, mmHg	Mean (SD)	78.8 (14.7)	76.9 (12.5)	82.4 (15.7)	0.257	0.058	74.3 (13.2)	78.6 (11.5)	77.3 (13.4)	73.7 (11.6)	0.458
**Oxygen saturation, %**	Mean (SD)	94.3 (4.4)	93.3 (6.5)	97.4 (1.8)	0.831	**<0.001**	91.1 (9.6)	93.7 (4.7)	93.5 (6.2)	93.6 (8.7)	0.758
**Respiratory rate, /min**	Mean (SD)	22.6 (5.5)	23.5 (12.4)	14.4 (4.0)	0.262	**<0.001**	22.5 (4.6)	22.9 (11.3)	25.1 (15.7)	21.0 (6.2)	0.312
**WHO Clinical severity score (in full)**	No evidence of infection	0 (0.0%)	0 (0.0%)	29 (100.0%)	0.672	**<0.001**	0 (0.0%)	0 (0.0%)	0 (0.0%)	0 (0.0%)	0.100
	No limitation of activities	0 (0.0%)	0 (0.0%)	0 (0.0%)			0 (0.0%)	0 (0.0%)	0 (0.0%)	0 (0.0%)	
	Limitation of activities	1 (1.0%)	0 (0.0%)	0 (0.0%)			0 (0.0%)	0 (0.0%)	0 (0.0%)	0 (0.0%)	
	Hospitalized, no oxygen therapy	28 (26.9%)	50 (31.4%)	0 (0.0%)			3 (17.6%)	17 (30.4%)	24 (36.9%)	6 (28.6%)	
	Oxygen by mask or nasal prongs	51 (49.0%)	74 (46.5%)	0 (0.0%)			8 (47.1%)	30 (53.6%)	28 (43.1%)	8 (38.1%)	
	Non-invasive ventilation	15 (14.4%)	20 (12.6%)	0 (0.0%)			4 (23.5%)	7 (12.5%)	8 (12.3%)	1 (4.8%)	
	Mechanical ventilation	5 (4.8%)	5 (3.1%)	0 (0.0%)			0 (0.0%)	0 (0.0%)	3 (4.6%)	2 (9.5%)	
	Ventilation with organ support	4 (3.8%)	10 (6.3%)	0 (0.0%)			2 (11.8%)	2 (3.6%)	2 (3.1%)	4 (19.0%)	
	Death	0 (0.0%)	0 (0.0%)	0 (0.0%)			0 (0.0%)	0 (0.0%)	0 (0.0%)	0 (0.0%)	
COVID-19 diagnosis											
**PCR test**		104 (100.0%)	159 (100.0%)	0 (0.0%)	1.000	**<0.001**	17 (100.0%)	56 (100.0%)	65 (100.0%)	21 (100.0%)	1.000
Nosocomial		6 (5.8%)	7 (4.4%)	0 (0.0%)	0.772	0.598	0 (0.0%)	4 (7.1%)	3 (4.6%)	0 (0.0%)	0.666
**Antibody test**		0 (0.0%)	0 (0.0%)	27 (93.1%)	1.000	**<0.001**	0 (0.0%)	0 (0.0%)	0 (0.0%)	0 (0.0%)	1.000
Chest x-ray / CT scan											

		COVID-19		Control	p-value[(a)]	p-value[(b)]	Adjudicated Myocarditis Diagnosis				p-value
		Without Primary Outcome	With Primary Outcome				Not present	Unlikely	Probable	Very likely	
		n = 104	n = 159	n = 29			n = 17	n = 56	n = 65	n = 21	
	**Typical of COVID-19**	72 (69.9%)	109 (74.7%)	-			12 (75.0%)	40 (78.4%)	41 (68.3%)	16 (84.2%)	
	**Atypical of COVID-19**	10 (9.7%)	11 (7.5%)	-	0.848	-	2 (12.5%)	3 (5.9%)	4 (6.7%)	2 (10.5%)	**0.024**
	**Unlikely**	3 (2.9%)	4 (2.7%)	-			2 (12.5%)	0 (0.0%)	1 (1.7%)	1 (5.3%)	
	**Normal**	18 (17.5%)	22 (15.1%)	-			0 (0.0%)	8 (15.7%)	14 (23.3%)	0 (0.0%)	
COVID-19 treatment											
Oxygen		75 (72.1%)	109 (68.6%)	-	0.584	-	14 (82.4%)	39 (69.6%)	41 (63.1%)	15 (71.4%)	0.509
Non-invasive respiratory support		18 (17.3%)	31 (19.5%)	-	0.747	-	5 (29.4%)	9 (16.1%)	11 (16.9%)	6 (28.6%)	0.386
**Invasive ventilation**		9 (8.7%)	14 (8.8%)	-	1.000	-	2 (11.8%)	1 (1.8%)	5 (7.7%)	6 (28.6%)	**0.004**
IV inotrope		4 (3.8%)	7 (4.4%)	-	1.000	-	1 (5.9%)	2 (3.6%)	1 (1.5%)	3 (14.3%)	0.092
Antiviral		24 (23.1%)	42 (26.4%)	-	0.564	-	9 (52.9%)	15 (26.8%)	14 (21.5%)	4 (19.0%)	0.075
Steroid		45 (43.3%)	89 (56.0%)	-	0.058	-	12 (70.6%)	31 (55.4%)	36 (55.4%)	10 (47.6%)	0.557
Intensive Care Unit		9 (8.7%)	24 (15.1%)	-	0.133	-	5 (29.4%)	5 (8.9%)	8 (12.3%)	6 (28.6%)	0.048
Cardiovascular History											
Hypertension		30 (28.8%)	56 (35.2%)	10 (34.5%)	0.347	1.000	8 (47.1%)	21 (37.5%)	18 (27.7%)	9 (42.9%)	0.326
Renal impairment		11 (10.6%)	7 (4.4%)	0 (0.0%)	0.078	0.598	1 (5.9%)	1 (1.8%)	4 (6.2%)	1 (4.8%)	0.562
Diabetes mellitus		25 (24.0%)	35 (22.0%)	2 (6.9%)	0.764	0.075	2 (11.8%)	17 (30.4%)	13 (20.0%)	3 (14.3%)	0.294
Hypercholesterolaemia		47 (45.2%)	76 (47.8%)	13 (44.8%)	0.706	0.841	10 (58.8%)	30 (53.6%)	28 (43.1%)	8 (38.1%)	0.399
Smoking	Never	62 (59.6%)	106 (66.7%)	18 (62.1%)			12 (70.6%)	38 (67.9%)	44 (67.7%)	12 (57.1%)	
	Former	36 (34.6%)	44 (27.7%)	9 (31.0%)	0.458	0.737	4 (23.5%)	17 (30.4%)	17 (26.2%)	6 (28.6%)	0.530
	Current	6 (5.8%)	9 (5.7%)	2 (6.9%)			1 (5.9%)	1 (1.8%)	4 (6.2%)	3 (14.3%)	
Percutaneous coronary intervention		1 (1.0%)	10 (6.3%)	0 (0.0%)	0.054	0.365	3 (17.6%)	2 (3.6%)	4 (6.2%)	1 (4.8%)	0.215
Coronary artery bypass graft surgery		2 (1.9%)	2 (1.3%)	0 (0.0%)	0.649	1.000	0 (0.0%)	1 (1.8%)	1 (1.5%)	0 (0.0%)	1.000
CCS Angina Class	No Angina	96 (92.3%)	154 (96.9%)	29 (100.0%)	0.144	1.000	16 (94.1%)	55 (98.2%)	64 (98.5%)	19 (90.5%)	0.180
	Angina Class I-IV	8 (7.7%)	5 (3.1%)	0 (0.0%)			1 (5.9%)	1 (1.8%)	1 (1.5%)	2 (9.5%)	
Heart failure		1 (1.0%)	6 (3.8%)	0 (0.0%)	0.250	0.593	0 (0.0%)	2 (3.6%)	3 (4.6%)	1 (4.8%)	1.000
Myocardial infarction		7 (6.7%)	17 (10.7%)	0 (0.0%)	0.382	0.079	3 (17.6%)	6 (10.7%)	6 (9.2%)	2 (9.5%)	0.758
Stroke or transient ischemic attack		2 (1.9%)	5 (3.1%)	3 (10.3%)	0.707	0.108	1 (5.9%)	1 (1.8%)	3 (4.6%)	0 (0.0%)	0.625
Peripheral vascular disease		2 (1.9%)	1 (0.6%)	0 (0.0%)	0.564	1.000	1 (5.9%)	0 (0.0%)	0 (0.0%)	0 (0.0%)	0.107
Cardiovascular disease and/or treatment		52 (50.0%)	74 (46.5%)	14 (48.3%)	0.615	1.000	8 (47.1%)	29 (51.8%)	26 (40.0%)	11 (52.4%)	0.560
Risk Scores											
**ISARIC-4c Mortality Score**	Mean (SD)	6.76 (3.53)	6.36 (3.33)	4.38 (3.30)	0.368	**0.003**	7.00 (3.26)	6.71 (3.42)	5.88 (3.06)	6.38 (3.94)	0.426
**ISARIC-4c Mortality Risk, in %**	Mean (SD)	13.5 (11.5)	12.1 (10.6)	6.9 (8.4)	0.368	**0.003**	14.0 (10.7)	13.2 (11.4)	10.4 (9.4)	12.8 (11.7)	0.426
QRisk3 10y CVD Risk, in %	Mean (SD)	16.0 (14.0)	13.5 (11.1)	13.1 (10.0)	0.401	0.984	12.5 (7.9)	15.5 (12.8)	12.0 (9.7)	14.3 (13.1)	0.724

		COVID-19		Control	p-value[(a)]	p-value[(b)]	Adjudicated Myocarditis Diagnosis				p-value
		Without Primary Outcome	With Primary Outcome				Not present	Unlikely	Probable	Very likely	
		n = 104	n = 159	n = 29			n = 17	n = 56	n = 65	n = 21	
Charlson Comorbidity Index	Mean (SD)	2.10 (2.21)	1.89 (1.82)	1.45 (1.21)	0.747	0.412	1.71 (1.93)	2.07 (2.01)	1.86 (1.79)	1.62 (1.24)	0.793
Pre-existing maintenance medication											
Aspirin		8 (7.7%)	12 (7.5%)	0 (0.0%)	1.000	0.218	3 (17.6%)	4 (7.1%)	4 (6.2%)	1 (4.8%)	0.419
Statin		25 (24.0%)	46 (28.9%)	10 (34.5%)	0.398	0.659	7 (41.2%)	20 (35.7%)	13 (20.0%)	6 (28.6%)	0.159
Beta-blocker		15 (14.4%)	20 (12.6%)	2 (6.9%)	0.712	0.537	3 (17.6%)	7 (12.5%)	5 (7.7%)	5 (23.8%)	0.208
ACE inhibitor		20 (19.2%)	35 (22.0%)	4 (13.8%)	0.644	0.456	6 (35.3%)	11 (19.6%)	13 (20.0%)	5 (23.8%)	0.542
ARB		9 (8.7%)	10 (6.3%)	2 (6.9%)	0.476	1.000	0 (0.0%)	6 (10.7%)	3 (4.6%)	1 (4.8%)	0.447
Oral anticoagulation		6 (5.8%)	8 (5.0%)	1 (3.4%)	0.786	1.000	1 (5.9%)	3 (5.4%)	3 (4.6%)	1 (4.8%)	1.000
Blood results, index admission											
Haemoglobin, g/l	Mean (SD)	141 (17)	141 (16)	143 (12)	0.970	0.655	142 (15)	140 (17)	140 (15)	143 (16)	0.624
**Platelet count, 10^9/l**	Mean (SD)	238 (84)	237 (94)	259 (58)	0.435	**0.006**	264 (137)	217 (75)	244 (93)	248 (95)	0.344
White cell count, 10^9/l	Mean (SD)	7.56 (3.34)	7.29 (5.52)	7.51 (3.11)	0.055	0.172	7.32 (4.82)	6.68 (2.65)	7.73 (7.76)	7.50 (2.98)	0.676
**Lymphocyte count, 10^9/l**	Mean (SD)	1.21 (0.67)	1.53 (4.66)	1.88 (0.64)	0.823	**<0.001**	1.02 (0.48)	1.08 (0.52)	2.11 (7.26)	1.35 (0.65)	0.215
**Peak D-Dimer, ng/ml**	Mean (SD)	1511 (4463)	1740 (5493)	311 (303)	0.722	**0.026**	2022 (4159)	916 (2132)	1754 (6648)	3127 (7431)	0.881
**Peak HbA1c, mmol/mol**	Mean (SD)	47.6 (16.9)	48.0 (18.4)	44.2 (22.1)	0.864	**0.020**	57.3 (32.3)	50.5 (18.1)	44.8 (13.5)	44.9 (19.2)	0.100
Initial creatinine, mmol/l	Mean (SD)	84.3 (29.6)	81.1 (37.1)	75.9 (24.2)	0.069	0.564	96.9 (55.3)	73.8 (21.9)	78.3 (28.2)	96.6 (62.7)	0.316
**Initial eGFR, ml/min/1.73m^2**	Mean (SD)	83.7 (21.8)	88.5 (23.4)	89.5 (19.5)	**0.034**	0.900	82.5 (27.7)	92.6 (18.7)	89.4 (23.5)	79.8 (28.4)	0.376
Initial eGFR <60 ml/min/1.73m^2		16 (15.4%)	19 (11.9%)	1 (3.4%)	0.460	0.322	2 (11.8%)	4 (7.1%)	8 (12.3%)	5 (23.8%)	0.256
Peak creatinine, µmol/l	Mean (SD)	92 (40)	103 (95)	90 (46)	0.123	0.752	99 (54)	89 (57)	96 (79)	168 (187)	0.506
Minimum eGFR, ml/min/1.73m^2	Mean (SD)	79.2 (22.8)	81.6 (27.4)	77.8 (29.0)	0.124	0.799	79.8 (27.1)	84.8 (23.4)	83.5 (26.5)	68.7 (37.0)	0.454
Minimum eGFR <60 ml/min/1.73m^2		23 (22.3%)	28 (17.6%)	1 (20.0%)	0.425	1.000	2 (11.8%)	7 (12.5%)	12 (18.5%)	7 (33.3%)	0.200
**Peak ferritin, µg/l**	Median (IQR)	348 (107, 919)	360 (180, 864)	118 (69, 166)	0.333	**<0.001**	454 (184, 835)	359 (212, 1082)	332 (154, 692)	562 (198, 1860)	0.441
**Initial high sensitivity troponin I, ng/l**	Median (IQR)	4 (2, 7)	4 (3, 10)	4 (4, 4)	**0.003**	0.519	5 (4, 8)	4 (3, 8)	4 (3, 7)	16 (4, 51)	0.146
**Initial high sensitivity troponin I, males**	<1.2 pg/ml<br>1.2 - 5.0 pg/ml<br>>5 - 34 pg/ml<br>>34 pg/ml	7 (11.5%)<br>33 (54.1%)<br>16 (26.2%)<br>5 (8.2%)	0 (0.0%)<br>44 (55.7%)<br>25 (31.6%)<br>10 (12.7%)	0 (0.0%)<br>15 (88.2%)<br>2 (11.8%)<br>0 (0.0%)	**0.017**	0.052	0 (0.0%)<br>6 (54.5%)<br>3 (27.3%)<br>2 (18.2%)	0 (0.0%)<br>16 (57.1%)<br>9 (32.1%)<br>3 (10.7%)	0 (0.0%)<br>18 (58.1%)<br>8 (25.8%)<br>5 (16.1%)	0 (0.0%)<br>4 (44.4%)<br>5 (55.6%)<br>0 (0.0%)	0.745
**Initial high sensitivity troponin I, females**	<1.2 pg/ml<br>1.2 - 5.0 pg/ml<br>>5 - 16 pg/ml<br>>16 pg/ml	6 (15.8%)<br>23 (60.5%)<br>7 (18.4%)<br>2 (5.3%)	5 (8.5%)<br>37 (62.7%)<br>8 (13.6%)<br>9 (15.3%)	0 (0.0%)<br>7 (77.8%)<br>2 (22.2%)<br>0 (0.0%)	0.320	0.557	1 (25.0%)<br>1 (25.0%)<br>2 (50.0%)<br>0 (0.0%)	2 (10.0%)<br>14 (70.0%)<br>3 (15.0%)<br>1 (5.0%)	1 (3.8%)<br>21 (80.8%)<br>3 (11.5%)<br>1 (3.8%)	1 (11.1%)<br>1 (11.1%)<br>0 (0.0%)<br>7 (77.8%)	**<0.001**

		COVID-19		Control	p-value[(a)]	p-value[(b)]	Adjudicated Myocarditis Diagnosis				p-value
		Without Primary Outcome	With Primary Outcome				Not present	Unlikely	Probable	Very likely	
		n = 104	n = 159	n = 29			n = 17	n = 56	n = 65	n = 21	
**Initial high sensitivity troponin I**	<1.2 pg/ml	13 (13.1%)	5 (3.6%)	0 (0.0%)	**0.031**	0.061	1 (6.7%)	2 (4.2%)	1 (1.8%)	1 (5.6%)	**0.041**
	1.2 - 5.0 pg/ml	56 (56.6%)	81 (58.7%)	22 (84.6%)			7 (46.7%)	30 (62.5%)	39 (68.4%)	5 (27.8%)	
	>5 pg/ml - 99th percentile	23 (23.2%)	33 (23.9%)	4 (15.4%)			5 (33.3%)	12 (25.0%)	11 (19.3%)	5 (27.8%)	
	>99th percentile	7 (7.1%)	19 (13.8%)	0 (0.0%)			2 (13.3%)	4 (8.3%)	6 (10.5%)	7 (38.9%)	
**Peak high sensitivity troponin I, ng/l**	Median (IQR)	4.0 (2.0, 10.5)	4.0 (3.0, 12.8)	4.0 (4.0, 4.0)	**0.009**	0.358	6.0 (4.0, 10.0)	4.0 (3.0, 10.0)	4.0 (3.0, 9.0)	30.0 (3.5, 83.8)	0.158
**Peak high sensitivity troponin I, males**	<1.2 pg/ml	6 (9.8%)	0 (0.0%)	0 (0.0%)	**0.020**	**0.044**	0 (0.0%)	0 (0.0%)	0 (0.0%)	0 (0.0%)	0.906
	1.2 - 5.0 pg/ml	29 (47.5%)	43 (54.4%)	14 (87.5%)			6 (54.5%)	15 (53.6%)	18 (58.1%)	4 (44.4%)	
	>5 - 34 pg/ml	19 (31.1%)	21 (26.6%)	2 (12.5%)			3 (27.3%)	9 (32.1%)	6 (19.4%)	3 (33.3%)	
	>34 pg/ml	7 (11.5%)	15 (19.0%)	0 (0.0%)			2 (18.2%)	4 (14.3%)	7 (22.6%)	2 (22.2%)	
Peak high sensitivity troponin I, females	<1.2 pg/ml	6 (15.8%)	5 (8.5%)	0 (0.0%)	0.559	0.362	1 (25.0%)	2 (10.0%)	1 (3.8%)	1 (11.1%)	**<0.001**
	1.2 - 5.0 pg/ml	21 (55.3%)	35 (59.3%)	7 (77.8%)			0 (0.0%)	14 (70.0%)	20 (76.9%)	1 (11.1%)	
	>5 - 16 pg/ml	6 (15.8%)	7 (11.9%)	2 (22.2%)			2 (50.0%)	3 (15.0%)	2 (7.7%)	0 (0.0%)	
	>16 pg/ml	5 (13.2%)	12 (20.3%)	0 (0.0%)			1 (25.0%)	1 (5.0%)	3 (11.5%)	7 (77.8%)	
**Peak high sensitivity troponin I**	<1.2 pg/ml	12 (12.1%)	5 (3.6%)	0 (0.0%)	**0.034**	**0.021**	1 (6.7%)	2 (4.2%)	1 (1.8%)	1 (5.6%)	**0.013**
	1.2 - 5.0 pg/ml	50 (50.5%)	78 (56.5%)	21 (84.0%)			6 (40.0%)	29 (60.4%)	38 (66.7%)	5 (27.8%)	
	>5 pg/ml - 99th percentile	25 (25.3%)	28 (20.3%)	4 (16.0%)			5 (33.3%)	12 (25.0%)	8 (14.0%)	3 (16.7%)	
	>99th percentile	12 (12.1%)	27 (19.6%)	0 (0.0%)			3 (20.0%)	5 (10.4%)	10 (17.5%)	9 (50.0%)	
**Peak fibrinogen, g/l**	Mean (SD)	7.13 (2.37)	6.04 (2.54)	3.41 (0.48)	0.376	**0.003**	6.70 (2.75)	4.69 (2.70)	5.65 (2.22)	7.83 (1.72)	0.240
**Peak CRP, mg/l**	Median (IQR)	131 (40, 206)	104 (37, 181)	2 (1, 5)	0.385	**<0.001**	130 (77, 180)	107 (45, 164)	91 (35, 181)	121 (17, 350)	0.584
**Peak CRP, mg/l**	<5 mg/l	9 (8.7%)	10 (6.3%)	20 (71.4%)	0.529	**<0.001**	0 (0.0%)	2 (3.6%)	4 (6.2%)	4 (19.0%)	0.274
	≥5, <10 mg/l	2 (1.9%)	6 (3.8%)	4 (14.3%)			0 (0.0%)	3 (5.4%)	3 (4.6%)	0 (0.0%)	
	≥10 mg/l	92 (89.3%)	143 (89.9%)	4 (14.3%)			17 (100.0%)	51 (91.1%)	58 (89.2%)	17 (81.0%)	
Peak sodium, mmol/l	Mean (SD)	140.1 (4.3)	140.3 (4.0)	139.4 (2.5)	0.336	0.157	138.8 (4.2)	139.8 (3.2)	140.8 (3.8)	141.8 (5.6)	0.233
**Albumin, g/l**	Mean (SD)	34.0 (4.9)	34.2 (5.2)	40.0 (5.3)	0.756	**<0.001**	32.1 (5.0)	35.0 (4.4)	33.8 (6.0)	34.8 (4.8)	0.273
Initial MCV, fl	Mean (SD)	- (-)	89.6 (6.1)	91.6 (4.1)	-	0.109	89.0 (4.5)	88.4 (7.2)	90.5 (5.7)	90.5 (5.0)	0.551
Initial RDW, %	Mean (SD)	- (-)	13.1 (1.4)	13.1 (0.5)	-	0.164	13.1 (0.8)	13.3 (1.9)	13.1 (1.0)	13.1 (1.2)	0.825
Peak MCV, fl	Mean (SD)	- (-)	92.8 (6.3)	91.9 (4.2)	-	0.221	92.9 (4.8)	90.9 (6.6)	93.6 (5.8)	95.2 (6.7)	0.094
RDW at peak MCV, %	Mean (SD)	- (-)	13.3 (1.3)	13.2 (0.6)	-	0.602	13.3 (0.9)	13.3 (1.5)	13.3 (1.2)	13.8 (1.6)	0.399

Table S1: [(a)] COVID-19 patients, without primary outcome vs. with primary outcome. [(b)]: COVID-19 patients with primary outcome vs. controls. Ethnicity: Indian (0), Pakistani (0), Bangladeshi (0), Other Asian n=14 (8.8%), Black Caribbean (0), Black African n=2 (1.2%), Chinese n=1 (0.6%), Other (n=1 (0.9%), White, n=139 (87.4%). Missing data in post-COVID-19 patients: D-Dimer, n=62; HbA1c, n=23; ferritin, n=18; troponin I, n=21. Missing data in Control patients: D-Dimer, n=15; HbA1c, n=5; ferritin, n=5; troponin I, n=4. CCS – Canadian Cardiovascular Society; CRP – C-reactive protein; GFR - glomerular filtration rate was estimated using the Chronic Kidney Disease Epidemiology equation[6], ISARIC-4C - Coronavirus Clinical Characterisation Consortium; MCV – mean cell volume; PCR – polymerase chain reaction; SD – standard deviation; RDW – red cell distribution width; SIMD - Scottish Index of Multiple Deprivation; TIA – transient ischaemic attack; WHO – World Health Organization. In the control group, the Abbott Architect CMIA SARS-CoV-2 IgG assay[§] was used to confirm absence of prior infection with COVID-19. The primary outcome evaluation (visit 2) was scheduled 28-60 days post-discharge. *Categorical data are summarized as frequency and percentage, and compared between groups using Fisher's Exact tests. Continuous data are summarized as mean and standard deviation, or median and interquartile range (IQR, defined as the upper and lower quartiles), and compared between groups using Kruskal-Wallis tests. All p-values are two-sided. No adjustments were made for multiple comparisons.

Table S2. Expanded - Multisystem phenotypying: serial electrocardiography, biomarkers of inflammation, metabolism, renal function, and hemostasis, and heart, lung, and kidney imaging at 28-60 days post-discharge.

				Myocarditis				
	COVID-19<br>n = 159	Controls<br>n = 29	P-value	Not present<br>n = 17 (11%)	Unlikely<br>n = 56 (35%)	Probable<br>n = 65 (41%)	Very likely<br>n = 21 (13%)	P-value*
Electrocardiogram, n (%)								
Admission (n = 150)								
**Myopericarditis criteria**	31 (20.7)	0 (0)	**0.003**	3 (17.6)	9 (16.7)	13 (22.4)	6 (28.6)	0.646
Premature atrial contraction	2 (1.3)	0 (0)	1.000	0 (0.0)	0 (0.0)	2 (3.1)	0 (0.0)	0.710
**Premature ventricular contraction**	3 (1.9)	0 (0)	1.000	1 (5.9)	0 (0.0)	0 (0.0)	2 (9.5)	**0.013**
Atrial fibrillation or flutter, n = 9 missing	5 (3.3)	0 (0)	1.000	0 (0.0)	2 (3.6)	2 (3.4)	1 (4.8)	1.000
Enrolment (n = 147)								
**Myopericarditis criteria**	47 (32.0)	0 (0)	**<0.001**	3 (21.4)	16 (30.2)	20 (32.3)	8 (44.4)	0.586
Premature atrial contraction	7 (4.4)	0 (0)	0.598	1 (5.9)	3 (5.4)	2 (3.1)	1 (4.8)	0.840
Premature ventricular contraction	1 (0.6)	0 (0)	1.000	0 (0.0)	1 (1.8)	0 (0.0)	0 (0.0)	0.591
Atrial fibrillation or flutter	3 (2.0)	0 (0)	1.000	0 (0.0)	1 (1.9)	1 (1.6)	1 (5.9)	0.623
28-60 days post-discharge (n = 143)								
**Myopericarditis criteria**	33 (23.1)	0 (0)	**0.002**	2 (14.3)	10 (20.4)	14 (23.3)	7 (35.0)	0.546
Premature atrial contraction	8 (5.0)	0 (0)	0.612	1 (5.9)	3 (5.4)	3 (4.6)	1 (4.8)	1.000
Premature ventricular contraction	2 (1.3)	0 (0)	1.000	1 (5.9)	0 (0.0)	1 (1.5)	0 (0.0)	0.220
Atrial fibrillation or flutter	2 (1.4)	0 (0)	1.000	0 (0.0)	0 (0.0)	1 (1.6)	1 (5.0)	0.535

	COVID-19<br>n = 159	Controls<br>n = 29	P-value	Myocarditis<br>Not present<br>n = 17 (11%)	Unlikely<br>n = 56 (35%)	Probable<br>n = 65 (41%)	Very likely<br>n = 21 (13%)	P-value*
CT chest 28-60 days post-discharge								
**Ground glass opacity and/or consolidation, n (%)**	70 (44.6)	1 (4.2)	**<0.001**	10 (66.7)	26 (46.4)	24 (36.9)	10 (47.6)	0.201
**Reticulation and/or architectural distortion, n (%)**	47 (29.9)	1 (4.2)	**0.006**	6 (40.0)	15 (26.8)	18 (27.7)	8 (38.1)	0.600
Atelectasis, n (%)	13 (8.3)	0 (0.0)	0.222	1 (6.7)	7 (12.5)	4 (6.2)	1 (4.8)	0.600
Pulmonary arterial thrombus, n (%)	5 (3.3)	0 (0.0)	1.000	0 (0.0)	2 (3.6)	2 (3.1)	1 (5.3)	0.905
**Visual estimate of % of total lung area abnormal, mean (SD)**	14.3 (19.0)	0.1 (0.5)	**<0.001**	19.3 (22.5)	12.7 (17.6)	12.3 (17.5)	21.1 (23.4)	0.286
**<20%, n (%)**	107 (68.2)	23 (95.8)	**0.006**	9 (60.0)	38 (67.9)	48 (73.8)	12 (57.1)	0.324
**≥20%, n (%)**	38 (24.2)	0 (0.0)		3 (20.0)	15 (26.8)	14 (21.5)	6 (28.6)	
**≥50%, n (%)**	12 (7.6)	1 (4.2)		3 (20.0)	3 (5.4)	3 (4.6)	3 (14.3)	
CT coronary angiogram 28-60 days post-discharge								
Coronary calcium - Agatston score, mean (SD)	144 (502)	95 (297)	0.238	44 (61)	253 (730)	91 (363)	91 (162)	0.071
CADS-RADS score, n (%)								
Level 0	77 (50.0)	13 (52.0)		3 (21.4)	23 (41.1)	40 (63.5)	11 (52.4)	
Level 1	42 (27.3)	7 (32.0)		7 (50.0)	19 (33.9)	11 (17.5)	5 (23.8)	
Level 2	11 (7.1)	2 (8.0)	0.974	0 (0.0)	5 (8.9)	4 (6.3)	2 (9.5)	**0.013**
Level 3	7 (4.5)	1 (4.0)		2 (14.3)	1 (1.8)	4 (6.3)	0 (0.0)	
Level 4	14 (9.1)	1 (4.0)		1 (7.1)	8 (14.3)	2 (3.2)	3 (14.3)	
Level 5	3 (1.9)	0 (0.0)		1 (7.1)	0 (0.0)	2 (3.2)	0 (0.0)	
Obstructive coronary artery disease, n (%)	21 (13.9)	1 (4.0)	0.325	3 (20.0)	7 (12.7)	7 (11.3)	4 (21.1)	0.555
$FFR_{CT,}$ patient-level (all coronary arteries)								
Median FFR_{CT}, mean (SD)	0.93 (0.03)	0.93 (0.02)	0.109	0.92 (0.03)	0.93 (0.04)	0.93 (0.03)	0.92 (0.04)	0.308
Minimum FFR_{CT}, mean (SD)	0.80 (0.10)	0.84 (0.09)	0.006	0.82 (0.08)	0.79 (0.11)	0.81 (0.09)	0.76 (0.13)	0.541

	COVID-19 n = 159	Controls n = 29	P-value	Myocarditis Not present n = 17 (11%)	Unlikely n = 56 (35%)	Probable n = 65 (41%)	Very likely n = 21 (13%)	P-value*
Minimum FFR_{CT} ≤0.80, n (%)	50 (37.9)	7 (28.0)	0.377	5 (41.7)	16 (34.8)	20 (35.7)	9 (50.0)	0.655
Cardiovascular MRI 28-60 days post-discharge								
LV end-diastolic volume index, mean (SD), mL/m^2	75.9 (17.0)	73.9 (18.7)	0.326	77.2 (17.9)	74.1 (16.6)	77.4 (17.1)	75.2 (17.7)	0.881
**LV end-systolic volume index, mean (SD), mL/m^2**	35.3 (12.8)	30.2 (13.7)	**0.012**	34.6 (11.1)	33.7 (11.7)	36.3 (14.2)	36.6 (12.4)	0.815
**LV ejection fraction, mean (SD), %**	54.1 (9.7)	60.4 (9.3)	**<0.001**	54.8 (9.8)	55.1 (10.1)	54.0 (8.6)	51.3 (11.5)	0.433
LV ejection fraction reduced, males <48%, n (%)	19 (21.3)	2 (12.5)	0.518	2 (15.4)	6 (17.1)	8 (24.2)	3 (33.3)	0.665
**LV ejection fraction reduced, females <51%, n (%)**	12 (17.6)	0 (0.0)	0.346	1 (25.0)	0 (0.0)	6 (18.8)	5 (41.7)	**0.012**
**LV mass index, mean (SD), g/m^2**	91.8 (25.6)	119.4 (26.8)	**<0.001**	100.9 (18.9)	93.2 (21.5)	89.7 (27.8)	87.6 (31.6)	0.170
**LV global longitudinal strain, mean (SD), %**	-14.2 (3.0)	-14.5 (6.9)	**0.031**	-13.7 (3.1)	-13.9 (2.9)	-14.6 (3.0)	-14.1 (3.0)	0.375
**LV global circumferential strain, mean (SD), %**	-17.0 (3.3)	-17.9 (3.0)	0.153	-17.4 (3.6)	-17.0 (3.4)	-17.1 (3.1)	-16.6 (3.4)	0.801
**LV global radial strain, mean (SD), %**	28.3 (7.8)	30.6 (7.7)	0.164	29.3 (8.4)	28.4 (8.3)	28.3 (7.3)	27.1 (7.9)	0.780
**RV end-diastolic volume index, mean (SD), mL/m^2**	73.3 (17.7)	79.7 (14.1)	**0.019**	77.8 (18.7)	72.7 (19.7)	72.6 (17.0)	73.3 (13.9)	0.760
RV end-systolic volume index, mean (SD), mL/m^2	35.9 (11.3)	34.4 (10.0)	0.948	34.6 (12.4)	36.6 (11.9)	35.0 (11.5)	38.1 (8.3)	0.487
**RV ejection fraction, mean (SD), %**	50.9 (10.5)	58.5 (9.3)	**<0.001**	54.6 (15.9)	49.9 (9.5)	51.9 (9.0)	47.5 (11.4)	0.210
RV global longitudinal strain, mean (SD), %	-17.2 (5.9)	-19.0 (5.8)	0.165	-14.4 (9.5)	-17.5 (5.4)	-18.1 (5.3)	-15.6 (4.3)	0.130
Myocardial tissue characterization								
Increased global T1 (>1233 ms), n (%)	55 (34.8)	5 (19.2)	0.174	2 (12.5)	14 (25.0)	31 (46.2)	9 (42.9)	**0.016**
Increased global T2 (>44 ms), n (%)	10 (6.3)	0 (0.0)	0.312	0 (0.0)	0 (0.0)	6 (9.2)	4 (19.0)	**0.007**
T2 ratio (myocardium/ serratus anterior muscle)	1.7 (0.2)	1.6 (0.1)	0.180	1.6 (0.2)	1.6 (0.2)	1.8 (0.2)	1.8 (0.3)	**<0.001**
**Increased global extracellular volume (>27.4%), n (%)**	71 (49.7)	5 (20.8)	**0.014**	1 (7.7)	22 (41.5)	35 (60.3)	13 (68.4)	**<0.001**
Late gadolinium enhancement								
Myocardial late gadolinium enhancement, n (%)	30 (19.0)	2 (7.7)	0.262	4 (25.0)	7 (12.5)	13 (20.0)	6 (28.6)	0.329

	COVID-19 n = 159	Controls n = 29	P-value	Myocarditis Not present n = 17 (11%)	Unlikely n = 56 (35%)	Probable n = 65 (41%)	Very likely n = 21 (13%)	P-value*
Ischemic distribution, n (%)	8 (5.5)	0 (0.0)	0.658	0 (0.0)	2 (3.9)	5 (8.1)	1 (5.6)	0.769
**Non-ischemic distribution, n (%)**	24 (16.3)	2 (8.0)	0.606	4 (28.6)	5 (9.8)	8 (12.9)	7 (35.0)	**0.033**
Pericardial thickening, n (%)	33 (21.2)	0 (0.0)	0.176	1 (5.9)	10 (18.5)	15 (23.4)	7 (33.3)	0.197
Pericardial effusion, n (%)	16 (10.2)	0 (0.0)	0.135	0 (0.0)	5 (9.1)	7 (10.9)	4 (19.0)	0.297
Right atrial area, mean (SD), cm^2	18.7 (4.7)	19.5 (4.2)	0.369	19.2 (3.9)	18.8 (4.9)	18.1 (5.1)	19.9 (3.2)	0.202
Left atrial area, mean (SD), cm^2	20.7 (4.7)	21.8 (4.7)	0.210	22.3 (5.0)	19.9 (4.2)	20.5 (5.1)	21.9 (4.1)	0.204
Myocardial inflammation (Lake Louise criteria), n (%)								
**Probable (1/2)**	74 (46.8)	1 (3.4)	**<0.001**	4 (25.0)	49 (87.5)	21 (32.3)	0 (0.0)	**<0.001**
**Definite (2/2)**	67 (42.4)	1 (3.4)	**<0.001**	0 (0)	2 (3.6)	44 (67.7)	21 (100.0)	**<0.001**
Renal MRI, mean (SD)								
Average volume of right and left kidneys, ml	153 (31)	156 (33)	0.761	158 (37)	154 (25)	150 (33)	153 (38)	0.656
Average cortex T1 of right and left kidneys, ms	1544 (62)	1519 (70)	0.118	1548 (66)	1535 (58)	1541 (63)	1585 (60)	0.110
**Average medulla T1 of right and left kidneys, ms**	1934 (69)	1953 (59)	0.161	1935 (66)	1924 (65)	1925 (66)	2008 (57)	**0.003**
**Average T1 corticomedullary differentiation of kidneys**	0.80 (0.03)	0.78 (0.03)	**<0.001**	0.80 (0.03)	0.79 (0.03)	0.80 (0.03)	0.79 (0.02)	0.535
Biomarkers at enrolment, central laboratory								
**eGFR, median [IQR], ml/min/1.73m^2**	96 (85, 106)	89 (70, 98)	**0.025**	95 (88, 103)	94 (84, 107)	97 (83, 105)	98 (94, 104)	0.931
eGFR <60 ml/min/1.73m^2, n (%)	7 (4.7)	1 (4.8)	1.000	1 (6.2)	1 (1.9)	4 (6.6)	1 (5.3)	0.523
**C-reactive protein, median [IQR], mg/L**	5.5 (1.6, 22.3)	1.5 (0.8, 3.5)	**<0.001**	6.0 (1.6, 18.2)	5.5 (1.4, 22.8)	4.9 (1.8, 21.6)	14.0 (0.9, 21.5)	0.971
**Peak CRP category**								
**<5.0 mg/l**	75 (48.4%)	24 (88.9%)	**<0.001**	7 (41.2%)	27 (49.1%)	33 (52.4%)	8 (40.0%)	0.591
**≥5.0, <10.0 mg/l**	20 (12.9%)	2 (7.4%)		4 (23.5%)	8 (14.5%)	7 (11.1%)	1 (5.0%)	
**≥10.0 mg/l**	60 (38.7%)	1 (3.7%)		6 (35.3%)	20 (36.4%)	23 (36.5%)	11 (55.0%)	

	COVID-19 n = 159	Controls n = 29	P-value	Myocarditis Not present n = 17 (11%)	Unlikely n = 56 (35%)	Probable n = 65 (41%)	Very likely n = 21 (13%)	P-value*
High sensitivity troponin I, median [IQR], ng/L	3 (2, 6)	4 (4, 6)	0.059	4 (3, 5)	3 (2, 7)	3 (2, 5)	4 (3, 7)	0.609
**NT-proBNP, median [IQR], pg/mL**	114 (57, 262)	58 (38, 99)	**0.004**	108 (57, 246)	116 (65, 258)	93 (49, 278)	139 (65, 274)	0.546
**Ferritin, median [IQR], µg/L**	366 (202, 675)	186 (96, 254)	**<0.001**	428 (143, 576)	398 (281, 658)	313 (172, 683)	379 (187, 637)	0.529
**Haptoglobin, median [IQR], g/L**	2.1 (1.3, 3.1)	1.5 (1.2, 1.8)	**0.001**	2.2 (1.8, 3.2)	2.0 (1.5, 2.8)	1.9 (1.2, 3.1)	2.6 (1.6, 3.2)	0.738
Total cholesterol, mean (SD), mmol/L	4.8 (1.4)	4.9 (1.0)	0.530	4.8 (1.4)	4.6 (1.3)	5.0 (1.5)	5.0 (1.1)	0.384
**Triglycerides, mean (SD), mmol/L**	2.3 (1.3)	1.8 (1.3)	**0.016**	2.3 (1.3)	2.4 (1.4)	2.2 (1.2)	2.1 (0.8)	0.868
**HDL cholesterol, mean (SD), mmol/L**	1.1 (0.3)	1.2 (0.3)	**0.014**	1.0 (0.3)	1.0 (0.3)	1.1 (0.4)	1.1 (0.4)	0.432
**Prothrombin time, mean (SD), s**	12.1 (3.7)	11.2 (0.8)	0.106	12.1 (2.0)	12.7 (5.5)	11.7 (2.4)	12.0 (1.5)	**0.042**
**D-Dimer, mean (SD), ng/mL**	259 (221)	152 (149)	**<0.001**	290 (195)	263 (178)	246 (168)	260 (192)	0.374
**Fibrinogen, mean (SD), g/L**	4.1 (1.7)	3.2 (1.1)	**0.006**	3.9 (1.5)	4.1 (1.7)	4.0 (1.6)	4.5 (1.9)	0.659
**Factor VIII, mean (SD), IU/dL**	184 (94)	99 (39)	**<0.001**	208 (88)	183 (97)	181 (99)	173 (73)	0.527
Antithrombin, mean (SD), IU/dL	111 (18)	108 (14)	0.570	108 (18)	112 (18)	112 (17)	109 (20)	0.687
Protein C, mean (SD), IU/dL	123 (30)	116 (21)	0.194	121 (30)	120 (30)	127 (32)	123 (21)	0.574
Protein S, mean (SD), IU/dL	85 (28)	96 (26)	0.116	94 (33)	87 (32)	85 (25)	75 (23)	0.252
**VWF:GP1bR, mean (SD), IU/dL**	236 (127)	137 (70)	**<0.001**	257 (176)	241 (118)	224 (123)	246 (122)	0.755
**VWF:Ag, mean (SD), IU/dL**	243 (145)	204 (251)	**0.002**	310 (235)	233 (118)	228(135)	261 (140)	0.380
Biomarkers at 28-60 days post-discharge, central laboratory								
**eGFR, median [IQR], ml/min/1.73m^2**	95 (83, 106)	88 (70, 98)	**0.047**	91 (79, 103)	95 (82, 106)	95 (87, 105)	98 (79, 105)	0.954
eGFR <60 ml/min/1.73m^2, n (%)	7 (4.7)	1 (4.8)	1.000	1 (6.7)	1 (1.9)	4 (6.5)	1 (5.3)	0.514
C-reactive protein, median [IQR], mg/L	1.9 (0.9, 3.5)	1.5 (0.8, 3.5)	0.572	1.7 (1.1, 3.5)	2.0 (1.2, 3.4)	1.8 (0.8, 4.4)	1.9 (0.9, 3.1)	0.996
**Peak CRP category**								
**<5.0 mg/l**	124 (81.0%)	24 (88.9%)	0.770	14 (87.5%)	45 (81.8%)	2 (3.6%)	16 (80.0%)	0.562
**≥5.0, <10.0 mg/l**	16 (10.5%)	2 (7.4%)		1 (6.2%)	8 (14.5%)	6 (9.7%)	1 (5.0%)	

				Myocarditis				
	COVID-19<br>n = 159	Controls<br>n = 29	P-value	Not present<br>n = 17 (11%)	Unlikely<br>n = 56 (35%)	Probable<br>n = 65 (41%)	Very likely<br>n = 21 (13%)	P-value*
**≥10.0 mg/l**	13 (8.5%)	1 (3.7%)		1 (6.2%)	2 (3.6%)	7 (11.3%)	3 (15.0%)	
**High sensitivity troponin I, median [IQR], ng/L**	2 (1, 4)	4 (4, 6)	**<0.001**	2 (2, 4)	2 (1, 5)	2 (1, 4)	2 (1, 4)	0.941
**NT-proBNP, median [IQR], pg/mL**	83 (54, 198)	58 (38, 99)	**0.022**	60 (30, 172)	112 (65, 207)	87 (56, 148)	75 (52, 213)	0.290
Ferritin, median [IQR], ug/L	144 (72, 282)	186 (96, 254)	0.529	145 (86, 299)	158 (94, 296)	129 (57, 206)	157 (99, 319)	0.360
**Haptoglobin, median [IQR], g/L**	1.3 (0.9, 1.6)	1.5 (1.2, 1.8)	**0.031**	1.4 (1.1, 1.6)	1.2 (0.8, 1.6)	1.3 (0.8, 1.6)	1.3 (0.9, 1.8)	0.709
D-Dimer, mean (SD), ng/mL	205 (252)	152 (149)	0.146	171 (111)	207 (200)	194 (193)	266 (517)	0.965
Fibrinogen, mean (SD), g/L	3.4(1.4)	3.2 (1.1)	0.439	3.6 (2.4)	3.2 (0.9)	3.5 (1.3)	3.8 (1.7)	0.468
**Factor VIII, mean (SD), IU/dL**	149 (65)	108 (58)	**<0.001**	151 (96)	141 (54)	151 (70)	160 (50)	0.606
Protein S, mean (SD), IU/dL	100 (22)	100 (26)	0.260	107 (21)	105 (21)	96 (22)	92 (17)	0.062
VWF:GP1bR, mean (SD), IU/dL	143 (80)	137 (70)	0.912	138 (104)	136 (76)	144 (79)	165 (77)	0.345
VWF:Ag, mean (SD), IU/dL	164 (97)	204 (251)	0.479	151 (79)	155 (85)	157 (88)	224 (144)	0.091
Urine Biomarkers								
Albumin: creatinine ratio at enrolment, mean (SD)	3.2 (8.0)	6.2 (26.9)	0.173	1.6 (3.1)	4.5 (12.0)	2.3 (4.3)	4.1 (6.5)	0.758
Albumin: creatinine ratio at 28-60 days post-discharge, mean (SD)	3.8 (10.9)	6.2 (26.9)	0.257	5.1 (13.4)	5.1 (15.2)	3.4 (5.4)	3.8 (6.4)	0.900

Table S2: Missing data in post-COVID-19 patients (admission, enrolment, 28-60 days) and controls – myopericarditis criteria – n=9, n=12, n=16, n=0; Missing data in post-COVID-19 patients at 28-60 days and controls – CT chest atelectasis, reticulation, ground glass – n=2, n=5; pulmonary arterial thrombus – n=8, n=6; CT coronary angiogram 28-60 days and controls: Agatston score – n=7, n=4; CAD-RADS score – n=5, n=4; FFR_{CT} – n=27, n=4; Cardiovascular magnetic resonance imaging 28-60 days post-discharge: left ventricular end-diastolic volume index, left ventricular end-systolic volume index, left ventricular ejection fraction, left ventricular strain – n=2, n=3; left ventricular mass – n=2, n=3; right ventricular end-diastolic volume index, right ventricular systolic volume index, n=4, n=3; right ventricular ejection fraction, n=3, n=3; global T1 – n=1, n=3; global T2 – n=1, n=3; global extracellular volume – n=16, n=5; late gadolinium enhancement – n=1, n=3; ischemic distribution – n=14, n=4; non-ischemic distribution – n=12, n=4; mixed distribution – n=14, n=4; pericardial thickening – n=3, n=3; pericardial effusion – n=2, n=3; right and left atrial area – n=1, n=3; myocardial inflammation – n=1, n=0. Missing data in blood biomarkers, post-COVID-19 patients (enrolment and 28-60 days) and controls – eGFR – n=9, n=10, n= 8; C-reactive protein – n=4, n=6, n=2; High sensitivity troponin I – n=6, n=8, n=2; NT-proBNP – n=6, n=10, n=2; Total cholesterol, triglycerides, HDL cholesterol – n=4, n=6, n=2; Fibrinogen – n=5, n=10, n=2; D-Dimer – n=5, n=9, n=2; Factor VIII – n=5, n=9, n=2; Antithrombin – n=5, n=10, n=3; Protein C – n=5, n=10, n=3; Protein S – n=3, n=11, n=3; VWF:GP1bR – n=6, n=9, n=2; VWF:Ag – n=5, n=9, n=2. Abbreviations – aPTT - activated partial thromboplastin time; CAD-RADS - Coronary Artery Disease - Reporting and Data System; ECV - extracellular volume; eGFR (CKD-EPI) – estimated glomerular filtration rate using the Chronic Kidney Disease Epidemiology (CKD-EPI) equation)[6]; EF – ejection fraction; EDV – end-diastolic volume; ESV – end-systolic volume; FFR_{CT} – fractional flow reserve computed tomography; HbA1c - hemoglobin A1c; HDL – high density lipoprotein; LV – left ventricle; MESA - Multi-ethnic study of atherosclerosis; NT-proBNP - N-terminal pro B-type natriuretic peptide; PT – prothrombin time; RV - right ventricle; T1 –

longitudinal relaxation time; T2 – transverse relaxation time; TCT - thrombin clotting time; vWF:Ag - von Willebrand factor antigen.

* Categorical data are summarized as frequency and percentage and compared between groups using Fisher's Exact tests. Continuous data are summarized as mean and standard deviation, or median and interquartile range (IQR, defined as the upper and lower quartiles), and compared between groups using Kruskal-Wallis tests. All p-values are two-sided. No adjustments were made for multiple comparisons.The Kendall's tau rank correlation between Lake Louise criteria, and the final adjudication (4 levels) was 0.75, or with 2 levels, was 0.72, representing moderately strong correlations.

Table S3. SARS-CoV-2 vaccination status.

	COVID-19		Controls
	Without Primary Outcome	With Primary outcome	
	(n = 104)	(n = 159)	(n = 29)
SARS-CoV-2 vaccination status, n (%)			
First dose prior to admission	1 (1.0)	2 (1.3)	-
Second dose prior to admission	0 (0.0)	0 (0.0)	-
First dose prior to imaging	-	11 (6.9)	23 (79.3)
Second dose prior to imaging	-	0 (0.0)	15 (51.7)
Third dose prior to imaging	-	0 (0.0)	0 (0.0)

Table S3 shows numbers and percentages. SARS-CoV-2 vaccines used in the NHS Scotland vaccination programme are the messenger RNA vaccines, BNT162b2 and mRNA-1273 manufactured by Biotech GmbH (Mainz, Germany) and Moderna (Cambridge, MA), respectively, and the AZD1222 (ChAdOx1 nCoV-19) recombinant SARS-CoV-2 vaccine manufactured by AstraZeneca (Cambridge, UK).

Table S4. Primary and secondary outcomes.

	Myocarditis					
	All cases<br>n=159	Not present<br>n=17 (11%)	Unlikely<br>n=56 (35%)	Probable<br>n=65 (41%)	Very likely<br>n=21 (13%)	P-value
	Cause of myocardial inflammation (endotype)					
SARS-COV-2 Myocarditis, n (%)						
Not present	16 (10.1)	16 (94.1)	0 (0.0)	0 (0.0)	0 (0.0)	**<0.001**
Unlikely	59 (37.1)	1 (5.9)	56 (100.0)	2 (3.1)	0 (0.0)	
Probable	76 (47.8)	0 (0.0)	0 (0.0)	62 (95.4)	14 (66.7)	
Very Likely	8 (5.0)	0 (0.0)	0 (0.0)	1 (1.5)	7 (33.3)	
Acute Stress Cardiomyopathy, n (%)						
Not present	89 (56.0)	15 (88.2)	37 (66.1)	26 (40.0)	11 (52.4)	**<0.001**
Unlikely	69 (43.4)	1 (5.9)	19 (33.9)	39 (60.0)	10 (47.6)	
Probable	1 (0.6)	1 (5.9)	0 (0.0)	0 (0.0)	0 (0.0)	
Very Likely	0 (0.0)	0 (0.0)	0 (0.0)	0 (0.0)	0 (0.0)	
Ischemia/ Impaired Perfusion as a Stressor of Inflammation, n (%)						
Not present	75 (47.2)	14 (82.4)	35 (62.5)	21 (32.3)	5 (23.8)	**<0.001**
Unlikely	73 (45.9)	2 (11.8)	17 (30.4)	39 (60.0)	15 (71.4)	
Probable	9 (5.7)	0 (0.0)	3 (5.4)	5 (7.7)	1 (4.8)	
Very Likely	2 (1.3)	1 (5.9)	1 (1.8)	0 (0.0)	0 (0.0)	
Infective Myopericarditis (non-Covid infection), n (%)						
Not present	42 (26.4)	17 (100.0)	12 (21.4)	11 (16.9)	2 (9.5)	**<0.001**
Unlikely	117 (73.6)	0 (0.0)	44 (78.6)	54 (83.1)	19 (90.5)	
Probable	0 (0.0)	0 (0.0)	0 (0.0)	0 (0.0)	0 (0.0)	
Very Likely	0 (0.0)	0 (0.0)	0 (0.0)	0 (0.0)	0 (0.0)	

	Myocarditis					
	All cases n=159	Not present n=17 (11%)	Unlikely n=56 (35%)	Probable n=65 (41%)	Very likely n=21 (13%)	P-value
Drug Induced (Toxic) Myocardial Inflammation, n (%)						
Not present	71 (44.7)	17 (100.0)	29 (51.8)	20 (30.8)	5 (23.8)	**<0.001**
Unlikely	88 (55.3)	0 (0.0)	27 (48.2)	45 (69.2)	16 (76.2)	
Probable	0 (0.0)	0 (0.0)	0 (0.0)	0 (0.0)	0 (0.0)	
Very Likely	0 (0.0)	0 (0.0)	0 (0.0)	0 (0.0)	0 (0.0)	
Idiopathic Myocardial and/or Pericardial Inflammation, n (%)						
Not present	29 (18.2)	17 (100.0)	6 (10.7)	4 (6.2)	2 (9.5)	**<0.001**
Unlikely	130 (81.8)	0 (0.0)	50 (89.3)	61 (93.8)	19 (90.5)	
Probable	0 (0.0)	0 (0.0)	0 (0.0)	0 (0.0)	0 (0.0)	
Very Likely	0 (0.0)	0 (0.0)	0 (0.0)	0 (0.0)	0 (0.0)	

Table S4 shows numbers and percentages, with p-values from Fisher's Exact tests. All p-values are two-sided. No adjustments were made for multiple comparisons. The exact p-values are : SARS-COV-2 Myocarditis, p=0.0001; Acute Stress Cardiomyopathy, p=0.0001; Ischemia/ Impaired Perfusion as a Stressor of Inflammation, p=0.0001; Infective Myopericarditis (non-Covid infection), p=0.0001; Drug Induced (Toxic) Myocardial Inflammation, p=0.0001; Idiopathic Myocardial and/or Pericardial Inflammation, p=0.0001.

Table S5. Diagnoses given by all raters in relation to the adjudicated diagnosis.

		All COVID-19	Adjudicated Myocarditis Diagnosis				p-value
			Not present	Unlikely	Probable	Very likely	
		(n=795)	(n=85)	(n=280)	(n=335)	(n=105)	
Individual rater assessment of the likelihood of myocarditis	Not Present	111 (14.0%)	74 (87.1%)	36 (12.9%)	1 (0.3%)	0 (0.0%)	
	Unlikely	223 (28.1%)	9 (10.6%)	171 (61.1%)	41 (12.6%)	2 (1.9%)	
	Probable	314 (39.5%)	2 (2.4%)	68 (24.3%)	214 (65.8%)	30 (28.6%)	**P<0.001**
	Very Likely	147 (18.5%)	0 (0.0%)	5 (1.8%)	69 (21.2%)	73 (69.5%)	

Table S5 shows numbers and percentages, with p-value from Fisher's Exact tests (two-sided). The denominators at the top of the table are five times the numbers of patients with each adjudicated diagnosis. E.g. with 21 patients adjudicated as being highly likely to have myocarditis, there were 105 individual assessment of this likelihood for these patients. The data illustrate a high level of concordance between the raters for myocarditis not present, and good discrimination between probably and very likely.

Table S6. Clinical and Diagnostic criteria for myocarditis, split by adjudicated likelihood of myocarditis diagnosis.

		COVID-19	Adjudicated Myocarditis Diagnosis				p-value
			Not present	Unlikely	Probable	Very likely	
		n=159	n=17	n=56	n=65	n=21	
Clinical criteria							
Chest pain		38 (23.9%)	7 (41.2%)	10 (17.9%)	13 (20.0%)	8 (38.1%)	0.081
New symptoms of breathlessness		149 (93.7%)	17 (100.0%)	51 (91.1%)	61 (93.8%)	20 (95.2%)	0.804
Chronic symptoms of breathlessness		2 (1.3%)	0 (0.0%)	1 (1.8%)	1 (1.5%)	0 (0.0%)	1.000
Cardiogenic shock		2 (1.3%)	1 (5.9%)	0 (0.0%)	1 (1.5%)	0 (0.0%)	0.220
Palpitations / Syncope / Arrhythmia		14 (8.8%)	1 (5.9%)	4 (7.1%)	7 (10.8%)	2 (9.5%)	0.905
Number of clinical criteria	0	7 (4.4%)	0 (0.0%)	4 (7.1%)	3 (4.6%)	0 (0.0%)	0.206
	1	104 (65.4%)	8 (47.1%)	40 (71.4%)	44 (67.7%)	12 (57.1%)	
	2	44 (27.7%)	9 (52.9%)	10 (17.9%)	16 (24.6%)	9 (42.9%)	
	3	3 (1.9%)	0 (0.0%)	2 (3.6%)	1 (1.5%)	0 (0.0%)	
	4	1 (0.6%)	0 (0.0%)	0 (0.0%)	1 (1.5%)	0 (0.0%)	
Diagnostic criteria							
ECG Myocarditis		30 (18.9%)	3 (17.6%)	9 (16.1%)	12 (18.5%)	6 (28.6%)	0.651
**T2 Criteria**		89 (56.0%)	0 (0.0%)	16 (28.6%)	52 (80.0%)	21 (100.0%)	**<0.001**
**T1 Criteria**		114 (71.7%)	0 (0.0%)	36 (64.3%)	57 (87.7%)	21 (100.0%)	**<0.001**
**Lake Louise Criteria**	**1 of 2**	73 (45.9%)	0 (0.0%)	52 (92.9%)	21 (32.3%)	0 (0.0%)	**<0.001**
	**2 of 2**	65 (40.9%)	0 (0.0%)	0 (0.0%)	44 (67.7%)	21 (100.0%)	
Elevated high sensitivity troponin		77 (48.4%)	6 (35.3%)	26 (46.4%)	31 (47.7%)	14 (66.7%)	0.267
**Global left ventricular dysfunction**		32 (20.1%)	3 (17.6%)	6 (10.7%)	14 (21.5%)	9 (42.9%)	**0.022**
**Regional left ventricular dysfunction**		34 (21.4%)	3 (17.6%)	5 (8.9%)	17 (26.2%)	9 (42.9%)	**0.006**

**Pericardial changes**		32 (20.1%)	0 (0.0%)	10 (17.9%)	15 (23.1%)	7 (33.3%)	**0.046**
**New left ventricular dysfunction**		29 (18.2%)	2 (11.8%)	5 (8.9%)	14 (21.5%)	8 (38.1%)	**0.022**
**Number of diagnostic criteria**	**0**	10 (6.8%)	10 (58.8%)	0 (0.0%)	0 (0.0%)	0 (0.0%)	**<0.001**
	**1-2**	28 (19.0%)	4 (23.5%)	20 (35.7%)	4 (6.7%)	0 (0.0%)	
	**3-4**	62 (42.2%)	3 (17.6%)	32 (57.1%)	24 (40.0%)	3 (21.4%)	
	**5-6**	37 (25.2%)	0 (0.0%)	3 (5.4%)	26 (43.3%)	8 (57.1%)	
	**7+**	10 (6.8%)	0 (0.0%)	1 (1.8%)	6 (10.0%)	3 (21.4%)	

Table S6 shows numbers and percentages, with p-values from Fisher's Exact tests. All p-values are two-sided. No adjustments were made for multiple comparisons.

Table S7. Serious adverse events occurring during the index admission.

	COVID-19 population		
	Without Primary Outcome (n = 104)	With Primary outcome (n = 159)	Fisher Test p-value
Index admission, n (%)			
Death	1 (1.0)	0 (0.0)	0.40
Myocardial infarction	1 (1.0)	7 (4.4)	0.16
Stroke	1 (1.0)	0 (0.0)	0.40
Heart failure	2 (1.9)	1 (0.6)	0.57
Pulmonary embolism	5 (4.8)	5 (3.1)	0.53
Deep vein thrombosis	0 (0.0)	1 (0.6)	1.00
Atrial fibrillation	6 (5.8)	5 (3.1)	0.36
Ventricular arrhythmia	0 (0.0)	0 (0.0)	-
Non-cardiovascular serious adverse event	0 (0.0)	0 (0.0)	-

Table S7 shows numbers and percentages, with p-values from Fisher's Exact tests. All p-values are two-sided. No adjustments were made for multiple comparisons.

Table S8. Clinical outcomes in the primary analysis population.

				Myocarditis				
	With Primary Outcome	Control	p-value[(a)]	Not present	Unlikely	Probable	Very likely	p-value [(b)]
	n = 159	n = 27*		n = 17 (11%)	n = 56 (35%)	n = 65 (41%)	n = 21 (13%)	
Duration of follow-up								
Days to Visit 3 or death, (median IQR)	419 (369, 451)	218 (208, 236)	<0.001	429 (416, 545)	414 (369, 437)	417 (369, 444)	454 (365, 546)	0.185
Outcomes, n (%)								
**Death or Hospitalization (Any Cause)**	24 (15.1)	2 (7.4)	0.356	3 (17.6)	10 (17.9)	9 (13.8)	2 (9.5)	0.845
Death (Any Cause)	2 (1.3)	0 (0.0)	0.663	0 (0.0)	0 (0.0)	2 (3.1)	0 (0.0)	0.413
Cardiovascular Death	1 (0.6)	0 (0.0)	0.888	0 (0.0)	0 (0.0)	1 (1.5)	0 (0.0)	0.701
Renal Death	0 (0.0)	0 (0.0)	-	0 (0.0)	0 (0.0)	0 (0.0)	0 (0.0)	-
Respiratory Death	0 (0.0)	0 (0.0)	-	0 (0.0)	0 (0.0)	0 (0.0)	0 (0.0)	-
**Hospitalization (Any Cause)**	24 (15.1)	2 (7.4)	0.356	3 (17.6)	10 (17.9)	9 (13.8)	2 (9.5)	0.845
Cardiovascular Hospitalization	7 (4.4)	2 (7.4)	0.340	1 (5.9)	5 (8.9)	1 (1.5)	0 (0.0)	0.177
Renal Hospitalization	2 (1.3)	0 (0.0)	0.680	1 (5.9)	1 (1.8)	0 (0.0)	0 (0.0)	0.526
Respiratory Hospitalization	8 (5.0)	0 (0.0)	0.242	2 (11.8)	2 (3.6)	2 (3.1)	2 (9.5)	0.335
Cardiovascular outcomes, n (%)								
**Myocardial infarction**	1 (0.6)	0 (0.0)	0.679	1 (5.9)	0 (0.0)	0 (0.0)	0 (0.0)	0.040
Percutaneous Coronary Intervention	3 (1.9)	0 (0.0)	0.481	1 (5.9)	1 (1.8)	0 (0.0)	1 (4.8)	0.300
Coronary Artery Bypass Grafting	0 (0.0)	0 (0.0)	-	0 (0.0)	0 (0.0)	0 (0.0)	0 (0.0)	-
**Cerebrovascular accident**	0 (0.0)	1 (3.7)	**0.016**	0 (0.0)	0 (0.0)	0 (0.0)	0 (0.0)	-
Heart Failure	2 (1.3)	1 (3.7)	0.232	0 (0.0)	1 (1.8)	1 (1.5)	0 (0.0)	0.886
Deep vein thrombosis	0 (0.0)	0 (0.0)	-	0 (0.0)	0 (0.0)	0 (0.0)	0 (0.0)	-
New atrial fibrillation	3 (1.9)	1 (3.7)	0.204	0 (0.0)	1 (1.8)	2 (3.1)	0 (0.0)	0.739

Ventricular tachycardia or fibrillation	0 (0.0)	0 (0.0)	-	0 (0.0)	0 (0.0)	0 (0.0)	0 (0.0)	-
Respiratory outcomes, n (%)								
**Pulmonary fibrosis**	9 (5.7)	0 (0.0)	0.336	0 (0.0)	0 (0.0)	4 (6.2)	5 (23.8)	**<0.001**
New diagnosis asthma	1 (0.6)	0 (0.0)	0.680	1 (5.9)	0 (0.0)	0 (0.0)	0 (0.0)	0.039
Pulmonary embolism	4 (2.5)	0 (0.0)	0.407	0 (0.0)	1 (1.8)	2 (3.1)	1 (4.8)	0.778
Long-term oxygen therapy	2 (1.3)	0 (0.0)	0.559	0 (0.0)	0 (0.0)	1 (1.5)	1 (4.8)	0.391
Secondary care (outpatients)								
**Any outpatient care**	108 (67.9)	7 (25.9)	**0.017**	12 (70.6)	35 (62.5)	44 (67.7)	17 (81.0)	0.184
Acute COVID-19 (<28 days)	15 (9.4)	0 (0.0)	0.102	2 (11.8)	3 (5.4)	9 (13.8)	1 (4.8)	0.340
Ongoing COVID-19 (28-84 days)	20 (12.6)	1 (3.7)	0.194	1 (5.9)	5 (8.9)	9 (13.8)	5 (23.8)	0.225
**Long COVID-19 (>84 days)**	58 (36.5)	1 (3.7)	**0.017**	9 (52.9)	13 (23.2)	27 (41.5)	9 (42.9)	0.122
Cardiology	22 (13.8)	1 (3.7)	0.320	3 (17.6)	9 (16.1)	6 (9.2)	4 (19.0)	0.650
**Respiratory**	55 (34.6)	1 (3.7)	**0.013**	8 (47.1)	12 (21.4)	27 (41.5)	8 (38.1)	0.102
Physiotherapy	22 (13.8)	1 (3.7)	0.232	2 (11.8)	4 (7.1)	12 (18.5)	4 (19.0)	0.264

Table S8: For clinical outcomes, data are the number and percentage of participants with at least one event during follow-up. P-values are from log rank tests of the time to first event. All p-values are two-sided. No adjustments were made for multiple comparisons. (a): COVID-19 patients with primary outcome vs. controls. (b): Comparing subgroups defined by likelihood of myocarditis. *Two controls developed COVID-19 after the imaging visit, hence they were excluded from this group.

Table S9. Deaths and adjudicated causes of death.

Participant ID	Date of consent	Date of death	Death during index hospitalisation, Y/N	Visit 2, Y/N	Myocarditis adjudicated very likely, Y/N	Acute kidney injury, Y/N	Adjudicated cause of death
1074	21/09/2020	16/12/2020	N	Y	N	N	Urosepsis
1104	08/10/2020	02/11/2020	N	N	N/A	N	Metastatic prostate cancer
1117	20/10/2020	15/08/2021	N	Y	N	N	Diffuse hypoxic brain injury secondary to out of hospital cardiac arrest and lower respiratory tract infection
2025	23/10/2020	16/11/2020	Y	N	N/A	N	COVID-19 pneumonitis

Table S9: The reporting period extends from the date of the first patient in (22 May 2020) to the last day of follow-up (13 December 2021). Participant details: Patient 1074 had chronic kidney disease stage 3B and the adjudicated likelihood of myocarditis for this patient was 'probably' (median score = 3; adjudicator scores: 2,3,3,3,3). The adjudicated likelihood of myocarditis for patient 1117 was 'probably' (median score = 3; adjudicator scores: 3,3,3,4,4).

Table S10. Medication prescription at follow-up.

	COVID-19	Control		Myocarditis				
				Not present	Unlikely	Probable	Very likely	
	N=159	N=27*	p-value[(a)]	n = 17 (11%)	n = 56 (35%)	n = 65 (41%)	n = 21 (13%)	p-value [(b)]
Medication, n (%)								
**Aspirin**	22 (13.8%)	0 (0.0%)	**0.048**	4 (23.5)	7 (12.5)	6 (9.2)	5 (23.8)	0.204
P2Y12 inhibitor	8 (5.0%)	3 (11.1%)	0.202	2 (11.8)	3 (5.4)	2 (3.1)	1 (4.8)	0.413
**Calcium channel blocker**	31 (19.5%)	6 (22.2%)	0.795	**6 (35.3)**	**13 (23.2)**	**6 (9.2)**	**6 (28.6)**	**0.021**
Angiotensin converting enzyme inhibitor	37 (23.3%)	5 (18.5%)	0.804	5 (29.4)	13 (23.2)	12 (18.5)	7 (33.3)	0.463
Angiotensin receptor blocker	15 (9.4%)	2 (7.4%)	1.000	1 (5.9)	7 (12.5)	6 (9.2)	1 (4.8)	0.802
Sacubitril/Valsartan	5 (3.1%)	0 (0.0%)	1.000	1 (5.9)	1 (1.8)	2 (3.1)	1 (4.8)	0.625
Statin	76 (47.8%)	15 (55.6%)	0.534	11 (64.7)	32 (57.1)	25 (38.5)	8 (38.1)	0.074
Mineralocorticoid receptor antagonist	2 (1.3%)	0 (0.0%)	1.000	0 (0.0)	0 (0.0)	2 (3.1)	0 (0.0)	0.710
Beta Blocker	33 (20.8%)	3 (11.1%)	0.301	4 (23.5)	9 (16.1)	12 (18.5)	8 (38.1)	0.190
**Thiazide**	14 (8.8%)	2 (7.4%)	1.000	**2 (11.8)**	**10 (17.9)**	**1 (1.5)**	**1 (4.8)**	**0.008**
Warfarin	1 (0.6%)	0 (0.0%)	1.000	0 (0.0)	1 (1.8)	0 (0.0)	0 (0.0)	0.591
Novel oral anticoagulant therapy	12 (7.5%)	3 (11.1%)	0.461	1 (5.9)	4 (7.1)	5 (7.7)	2 (9.5)	0.968
Inhaled Steroid	24 (15.1%)	2 (7.4%)	0.379	2 (11.8)	8 (14.3)	9 (13.8)	5 (23.8)	0.691
Inhaled Bronchodilator	31 (19.5%)	3 (11.1%)	0.422	2 (11.8)	11 (19.6)	11 (16.9)	7 (33.3)	0.349
Inhaled Anti-muscarinics	15 (9.4%)	1 (3.7%)	0.475	0 (0.0)	5 (8.9)	6 (9.2)	4 (19.0)	0.285
Sodium-glucose co-transporter-2 inhibitor	17 (10.7%)	1 (3.7%)	0.479	0 (0.0)	8 (14.3)	7 (10.8)	2 (9.5)	0.456
Glucagon-like peptide-1 agonist	4 (2.5%)	1 (3.7%)	0.548	1 (5.9)	1 (1.8)	1 (1.5)	1 (4.8)	0.388
Insulin	8 (5.0%)	0 (0.0%)	0.605	1 (5.9)	5 (8.9)	1 (1.5)	1 (4.8)	0.222
Other anti-diabetic	24 (15.1%)	1 (3.7%)	0.135	2 (11.8)	12 (21.4)	7 (10.8)	3 (14.3)	0.436
Loop diuretic	5 (3.1%)	1 (3.7%)	1.000	1 (5.9)	1 (1.8)	1 (1.5)	2 (9.5)	0.180

Oral Steroid	1 (0.6%)	1 (3.7%)	0.270	0 (0.0)	0 (0.0)	0 (0.0)	1 (4.8)	0.239
Antidepressant	28 (17.6%)	5 (18.5%)	1.000	2 (11.8)	5 (8.9)	15 (23.1)	6 (28.6)	0.087

Table S10: n (%) of participants who were taking the medication at follow-up. *Two controls developed COVID-19 after the imaging visit, hence they were excluded from this group. P-values are from Fisher's Exact tests. All p-values are two-sided. No adjustments were made for multiple comparisons. (a): COVID-19 patients with primary outcome vs. controls. (b): Comparing subgroups defined by likelihood of myocarditis.
