# Supplementary material for: A multisystem, cardio-renal investigation of post-COVID-19 illness
Source: Nat Med. 2022 May 23;28(6):1303–13. doi: 10.1038/s41591-022-01837-9 (PMC9205780; doi:10.1038/s41591-022-01837-9)
Supplement: Supplementary file 2 — Reporting Summary [file 41591_2022_1837_MOESM2_ESM.pdf]

nature research

Corresponding author(s): Colin Berry BSc MBChB PhD

Last updated by author(s): 14/4/22

Reporting Summary

Nature Research wishes to improve the reproducibility of the work that we publish. This form provides structure for consistency and transparency in reporting. For further information on Nature Research policies, see our Editorial Policies and the Editorial Policy Checklist.

Statistics

For all statistical analyses, confirm that the following items are present in the figure legend, table legend, main text, or Methods section.

n/a	Confirmed	
☐	☒	The exact sample size (*n*) for each experimental group/condition, given as a discrete number and unit of measurement
☐	☒	A statement on whether measurements were taken from distinct samples or whether the same sample was measured repeatedly
☐	☒	The statistical test(s) used AND whether they are one- or two-sided<br>*Only common tests should be described solely by name; describe more complex techniques in the Methods section.*
☐	☒	A description of all covariates tested
☐	☒	A description of any assumptions or corrections, such as tests of normality and adjustment for multiple comparisons
☐	☒	A full description of the statistical parameters including central tendency (e.g. means) or other basic estimates (e.g. regression coefficient) AND variation (e.g. standard deviation) or associated estimates of uncertainty (e.g. confidence intervals)
☐	☒	For null hypothesis testing, the test statistic (e.g. F, t, r) with confidence intervals, effect sizes, degrees of freedom and P value noted<br>*Give P values as exact values whenever suitable.*
☒	☐	For Bayesian analysis, information on the choice of priors and Markov chain Monte Carlo settings
☒	☐	For hierarchical and complex designs, identification of the appropriate level for tests and full reporting of outcomes
☒	☐	Estimates of effect sizes (e.g. Cohen's d, Pearson's r), indicating how they were calculated

Our web collection on statistics for biologists contains articles on many of the points above.

Software and code

Policy information about availability of computer code

Data collection	electronic case report form (CRF) developed by programmers in the Robertson Centre for Biostatistics. The eCRF served as a central information repository with restricted access based on centrally administered user rights determined by the chief investigator and coordinated by the Project Management team. The eCRF was developed in line with the protocol. Any changes to the eCRF required sponsor approval. Core laboratory analyses of source data (digital ECGs, biomarkers, CT, MR) were submitted by site research staff and uploaded directly to the Clinical Trials Unit (CTU) central server. These files were subject to quality assurance procedures administered by data management staff in the CTU.

Data analysis	R for Windows v4.0.4 (R Core Team (2021). R: A language and environment for statistical computing. R Foundation for Statistical Computing, Vienna, Austria. URL https://www.R-project.org/.)<br><br>Packages<br>- RODBC (Brian Ripley and Michael Lapsley (2021). RODBC: ODBC Database Access. R package version 1.3-19. https://CRAN.R-project.org/package=RODBC)<br>- chron (David James and Kurt Hornik (2020). chron: Chronological Objects which Can Handle Dates and Times. R package version 2.3-56.)<br>- QRISK3 (Yan Li, Matthew Sperrin, ClinRisk Ltd. and Tjeerd Pieter van Staa (2019). QRISK3: 10-Year Cardiovascular Disease Risk Calculator (QRISK3 2017). R package version 0.3.0. https://CRAN.R-project.org/package=QRISK3)<br>- eq5d (Fraser Morton and Jagtar Singh Nijjar (2021). eq5d: Methods for Analysing 'EQ-5D' Data and Calculating 'EQ-5D' Index Scores. R package version 0.10.0. https://CRAN.R-project.org/package=eq5d)<br>- score (Jaejoon Song (2015). score: A Package to Score Behavioral Questionnaires. R package version 1.0.2. https://CRAN.R-project.org/package=score)

For manuscripts utilizing custom algorithms or software that are central to the research but not yet described in published literature, software must be made available to editors and reviewers. We strongly encourage code deposition in a community repository (e.g. GitHub). See the Nature Research guidelines for submitting code & software for further information.

Data

Policy information about availability of data

All manuscripts must include a data availability statement. This statement should provide the following information, where applicable:
- Accession codes, unique identifiers, or web links for publicly available datasets
- A list of figures that have associated raw data
- A description of any restrictions on data availability

Code Availability
The statistical code is available online in Github: https://github.com/RobertsonCentre/cisco19/blob/main/CISCO19_Paper1_v1.R

Anonymised study data will be available on reasonable request by contacting the corresponding author(CB) via the Robertson Centre of Biostatistics. Please allow up to 1 week for a response. Professor Colin Berry, British Heart Foundation Glasgow Cardiovascular Research Centre, Institute of Cardiovascular and Medical Sciences, 126 University Place, University of Glasgow, Glasgow, G12 8TA, Scotland, UK. Telephone: +44 (0) 141 330 1671 or +44 (0) 141 951 5180. Fax +44 (0) 141 330 6794. Email: colin.berry@glasgow.ac.uk

Field-specific reporting

Please select the one below that is the best fit for your research. If you are not sure, read the appropriate sections before making your selection.

☒ Life sciences ☐ Behavioural & social sciences ☐ Ecological, evolutionary & environmental sciences

For a reference copy of the document with all sections, see nature.com/documents/nr-reporting-summary-flat.pdf

Life sciences study design

All studies must disclose on these points even when the disclosure is negative.

Sample size	A pre determined sample size calculation was devised by bio statistician co-authors. "To detect an association between pre-existing cardiac problems and the incidence of myocarditis (myocardial inflammation), an endotype of myocardial injury, at 2-4 weeks we have assumed 25% of patients with prior cardiovascular disease and the incidence of myocarditis in those with/without prior cardiac problems will be 33% and 10%, respectively. To have 80% power to detect this difference will require 140 participants (35 with cardiac problems, 105 without) to be scanned. We aim for 160 patients to attend the imaging visit, anticipating that 10-15% of the participants may have incomplete imaging data due to technical reasons e.g. imaging artefact or claustrophobia.”
Data exclusions	The exclusion criteria were: (1) contra-indication to magnetic resonance (MR) imaging (e.g., severe claustrophobia, metallic foreign body); and (2) lack of informed consent.
Replication	Intra-observer variability was assessed on a subset (10%) of clinical cases.
Randomization	This was a non-randomised, cohort study.
Blinding	The patients and the outcome assessors were blinded. Outcome assessments, including laboratory, MRI and CT analyses, and endpoint adjudication were undertaken in blinded researchers. The patients completed the questionnaires before undergoing the scans and they were unaware of the test results. For intra observer variability, 10% of the dataset was pseudo-anonymised and re-adjudicated.

Reporting for specific materials, systems and methods

We require information from authors about some types of materials, experimental systems and methods used in many studies. Here, indicate whether each material, system or method listed is relevant to your study. If you are not sure if a list item applies to your research, read the appropriate section before selecting a response.

Materials & experimental systems

n/a	Involved in the study
☒	☐ Antibodies
☒	☐ Eukaryotic cell lines
☒	☐ Palaeontology and archaeology
☒	☐ Animals and other organisms
☐	☒ Human research participants
☐	☒ Clinical data
☒	☐ Dual use research of concern

Methods

n/a	Involved in the study
☒	☐ ChIP-seq
☒	☐ Flow cytometry
☒	☐ MRI-based neuroimaging

Human research participants

Policy information about studies involving human research participants

Population characteristics

Patients who received hospital care for COVID-19, with or without admission, and were alive, were prospectively screened in real time using an electronic healthcare information system (TrakCare®, InterSystems®, USA) and daily hospital reports identifying inpatients with laboratory-positive results for COVID-19.

Recruitment

This study involved a prospective, observational, multicenter, longitudinal, care cohort design to assess for persisting multiorgan injury in survivors of COVID-19 during convalescence.
The inclusion criteria were: (1) age >18 years old; (2) history of unplanned hospital visit e.g., emergency department, or hospitalisation >24 hours for COVID-19, confirmed by a laboratory test e.g., PCR for SARS-CoV-2; (3) ability to comply with study procedures; (4) ability to provide written informed consent. The radiology results were reported by accredited radiologists according to contemporary, national guidelines 1.
The exclusion criteria were: (1) contra-indication to cardiovascular magnetic resonance imaging e.g., severe claustrophobia, metallic foreign body; and (2) lack of written informed consent.
A screening log was prospectively completed. The reasons for being ineligible, including lack of inclusion criteria and/or presence of exclusion criteria, were recorded.

Ethics oversight

The study was approved by the UK National Research Ethics Service (Reference 20/NS/0066).

Note that full information on the approval of the study protocol must also be provided in the manuscript.

Clinical data

Policy information about clinical studies

All manuscripts should comply with the ICMJE guidelines for publication of clinical research and a completed CONSORT checklist must be included with all submissions.

Clinical trial registration

ClinicalTrials.gov: NCT04403607.

Study protocol

The study protocol was peer reviewed and published.
14. Mangion K, Morrow A, Bagot C, Bayes H, Blyth KG, Church C, Corcoran D, Delles C, Gillespie L, Grieve D, Ho A, Kean S, Lang NN, Lennie V, Lowe DJ, Kellman P, Macfarlane PW, McConnachie A, Roditi G, Sykes R, Touyz RM, Sattar N, Wereski R, Wright S, Berry C. The Chief Scientist Office Cardiovascular and Pulmonary Imaging in SARS Coronavirus disease-19 (CISCO-19) study. Cardiovasc Res. 2020 Dec 1;116(14):2185-2196. doi: 10.1093/cvr/cvaa209.
This can be downloaded as an Open-access article from:
https://academic.oup.com/cardiovascres/article/116/14/2185/5875604

Data collection

The study involved three hospitals in the West of Scotland (population 2.2 million) - the Queen Elizabeth University Hospital and Glasgow Royal Infirmary and the Royal Alexandra Hospital in Paisley. One thousand three hundred and six patients were screened between 22 May 2020 and 16 March 2021 and 267 patients provided written informed consent. One hundred and fifty-nine patients were evaluated at 28-60 days after the last episode of hospital care.

Outcomes

The predefined primary outcome was a diagnosis of myocarditis (myocardial inflammation), an endotype of acute myocardial injury. The diagnostic criteria for myocarditis included relevant clinical findings and test results . Positive clinical findings included chest pain, pericarditic or pseudo-ischemic in nature, new onset breathlessness, subacute/chronic breathlessness, palpitations, unexplained arrhythmia, syncope, aborted sudden cardiac death, or unexplained cardiogenic shock. Positive test findings included 1) ECG features, 2) elevated troponin I, 3) functional and structural abnormalities on cardiac imaging (echocardiography, angiography, or MRI), and 4) tissue characterization MRI, including myocardial edema and late gadolinium enhancement with a distribution in alignment with the modified Lake Louise diagnostic criteria for myocarditis. Acute and chronic myocardial pathology can be identified, discriminated, and quantified using MRI.
Myocarditis was clinically suspected if at least 1 clinical finding and at least 1 diagnostic test criterion from different categories, in the absence of: (1) angiographically detectable coronary artery disease (coronary stenosis ≥ 50%); (2) known pre-existing cardiovascular disease or extra-cardiac causes that could explain the syndrome (e.g., valve disease, congenital heart disease, hyperthyroidism, etc.). Suspicion increases with a rising number of fulfilled criteria. If the patient was asymptomatic, at least 2 diagnostic criteria were required.

A diagnosis of myocarditis is susceptible to confounding through ascertainment bias. Recent studies in COVID-19 have not implemented the modified Lake Louise diagnostic criteria. Accordingly, in order to limit the potential for bias, we pre-specified an adjudication procedure for the primary outcome, involving a panel of experienced cardiologists. The panel reviews were undertaken according to a prespecified charter.
Consultant (board-certified) cardiologists (n=14) who were independent of the research team were invited as assessors. They were initially provided with information on the European Society of Cardiology Working Group on Myocardial and Pericardial Disease position statement on myocarditis4, a charter, and training cases. The cardiologists were blind to the identity of the patients and independent of their clinical care. The adjudications were coordinated by a researcher (A.M.) using Teams (Microsoft, Seattle, USA) software.
Each cardiologist independently assessed the clinical data, including the medical history, biomarkers, ECG, and radiology reports for the CMR, CT chest scans, including the pulmonary and coronary angiograms. Deidentified source clinical data e.g., scan images, were made available on request. The cardiologists determined the likelihood (not likely / unlikely / probable / very likely) of myocardial inflammation (myocarditis). The final diagnosis was based on the median likelihood based on the adjudications of 5 cardiologists. Their determinations were also categorized in binary form (not/unlikely = no; probable/very = yes).
